# Senescent Fibroblasts Drive Melanoma Progression Through GCP‐2 Induced CREB Phosphorylation Enhancing Glycolysis

**DOI:** 10.1111/acel.70239

**Published:** 2025-11-19

**Authors:** Abhijit Basu, Vida Farsam, Karmveer Singh, Diana Crisan, Nicolai Treiber, Lars Alexander Schneider, Margit Huber, Jennifer I. Engelmeyer, Björn Schumacher, Pallab Maity, Daniel Brandt, Martin Jastroch, Cornelia Mauch, Hartmut Geiger, Dimitris Kletsas, Karin Scharffetter‐Kochanek

**Affiliations:** ^1^ Department of Dermatology and Allergic Diseases University of Ulm Ulm Germany; ^2^ Aging Research Center (ARC) University of Ulm Ulm Germany; ^3^ Institute for Genome Stability in Ageing and Disease, Medical Faculty and Cologne Excellence Cluster for Cellular Stress Responses in Ageing‐Associated Diseases (CECAD) Research University of Cologne Cologne Germany; ^4^ Helmholtz Diabetes Center & German Diabetes Center (DZD), Helmholtz Zentrum München, Division of Metabolic Diseases Technische Universität München Neuherberg Germany; ^5^ Department of Molecular Biosciences The Wenner‐Gren Institute, Stockholm University Stockholm Sweden; ^6^ Department of Dermatology and Venereology University of Cologne Cologne Germany; ^7^ Department of Dermatology Ruhr University Bochum Bochum Germany; ^8^ Institute of Molecular Medicine and Stem Cell Aging University of Ulm Ulm Germany; ^9^ Laboratory of Cell Proliferation & Ageing Institute of Biosciences and Applications, NCSR “Demokritos” Athens Greece

**Keywords:** CREB, GCP‐2, melanoma metabolism, melanoma progression, SASP, senescent fibroblast

## Abstract

Aging constitutes the largest risk factor for melanoma progression. While a contribution of factors secreted from senescent skin fibroblasts to the progression of melanoma has been proposed, the nature of such factors and subsequent underlying mechanisms remains elusive. Here we show that the chemokine GCP‐2 is excessively released by senescent fibroblasts in vitro and the skin of old melanoma patients. GCP‐2 regulates, via phosphorylation of the transcription factor CREB at serine 133, defense‐, cell cycle control‐, and glycolysis‐enhancing genes in melanoma cell lines. GCP‐2 promotes oncogenic properties in vitro and in vivo in murine melanoma models. Inhibition of CREB phosphorylation in melanoma cells represses glycolytic target genes and induces a switch from glycolysis to oxidative phosphorylation that translates into a significant decline in tumor size in vivo in murine melanoma models. This study identifies a senescent fibroblast to chemokine to CREB to metabolic axis that drives melanoma progression. Targeting this axis may hold promise for novel therapeutic approaches in difficult‐to‐treat melanoma in older adults.

## Graphical abstract

Aging constitutes the largest risk factor for melanoma progression; the underlying reason is poorly understood. Here we show that the chemokine GCP‐2 is excessively released by senescent fibroblasts in vitro and in the skin of old melanoma patients. GCP‐2, via phosphorylation of the transcription factor CREB at serine 133, regulates cell cycle control and glycolysis‐enhancing genes in melanoma cell lines, thereby promoting oncogenic properties in vitro and in vivo in murine melanoma models. Inhibition of CREB phosphorylation in melanoma cells represses glycolytic target genes and induces a switch from glycolysis to oxidative phosphorylation that translates into a significant decline in tumor size in vivo in murine melanoma models. This study identifies a senescent fibroblast to chemokine to CREB to metabolic axis that drives melanoma progression. Targeting this axis may hold promise for novel therapeutic approaches in difficult‐to‐treat melanoma in old adults.

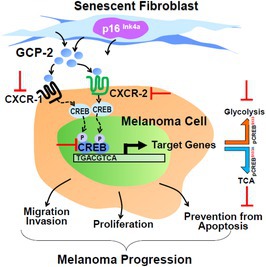

## 1 Introduction

Cutaneous malignant melanoma is the leading cause of death from skin cancer in Europe and the United States. Its high incidence and propensity to metastasize account for the vast morbidity and mortality of malignant melanoma. In addition, the development of resistance to new targeted and current immune therapies and combinations thereof (Fedorenko et al. 2011; Kim et al. 2013; Larkin et al. 2015; Nazarian et al. 2010; Prado et al. 2019; Ravnan and Matalka 2012) constitutes a major challenge. Malignant transformation of melanocytes into melanoma cells occurs in a multistep process with the gradual acquisition of several genetic mutations. The earliest and the most frequent of those is a point mutation (T1799A) in the proto‐oncogene which encodes BRAF^V600E^, a constitutively active serine kinase that activates the BRAF‐MEK1/2‐ERK1/2 MAP kinase pathway (Gray‐Schopfer et al. 2005). However, oncogenic activation of BRAF in melanocytes, the benign counterpart of melanoma cells, does not convert melanocytes into malignant melanoma. The ultimate transformation into malignant melanoma occurs in the presence of accompanying mutations of additional proto‐oncogenes or tumor suppressor genes (Dankort et al. 2009; Gray‐Schopfer et al. 2005). Even though the mutations responsible for the subsequent switch from radial growth to vertical growth and invasion phase of malignant melanoma are not fully elucidated (Greene et al. 2009), melanoma invasion strongly correlates with anchorage‐independent growth, increased motility, and poor prognosis. In fact, the depth of invasion, referred to as Breslow's index (tumor thickness in mm), constitutes the major prognostic factor reliably predicting the clinical outcome of melanomas (Breslow 1970; Clark Jr. et al. 1969; Cohen et al. 1987; Fernandez and Helm 2004). A striking link exists between advanced age and a marked increase in primary melanoma thickness (Fernandez and Helm 2004; Kruijff et al. 2012). This marked increase in thickness—in addition to more aggressive mutations and epigenetic changes accumulating with age—might also be fostered by a permissive tumor microenvironment in aged skin and other tissues. Upon aging, senescent fibroblasts accumulate in aged skin (Ressler et al. 2006) or other organs (Krishnamurthy et al. 2004). Senescent fibroblasts show changes in chromatin organization and gene expression and present with a release of a large variety of pro‐inflammatory chemokines, growth factors, and proteases. This is referred to as the senescence‐associated secretory phenotype (SASP) (Coppe et al. 2008; Davalos et al. 2010; Krtolica et al. 2001; Parrinello et al. 2005). Even though a profound impact of SASP factors released from senescent skin fibroblasts on the progression of melanoma has been supposed (Kim et al. 2013; Kuilman and Peeper 2009; Liu et al. 2023; Dong et al. 2024), the nature of such factors and subsequent underlying mechanisms remain elusive.

Based on an initial in vitro screen of SASP components, we here identified the chemokines granulocyte chemotactic protein‐2 (GCP‐2/CXCL6) and epithelial‐derived neutrophil attractant‐78 (ENA‐78/CXCL5), physiologically involved in neutrophil attraction to sites of tissue infection, to be upregulated in the skin of healthy old individuals and old but not young melanoma patients. Importantly, we here found that GCP‐2 promoted melanoma growth and prevented apoptosis by activation of the transcription factor CREB with subsequent expression of target genes enhancing glycolysis in melanoma cell lines in vitro and in vivo xenograft murine melanoma models in vivo. CREB activation was also observed in melanomas of aged but not in young human patients. Inhibition of CREB switches glycolysis to oxidative phosphorylation and, consequently, abrogates melanoma progression. In aggregate, these data uncover a previously unreported role of GCP‐2 released from peritumoral senescent fibroblasts, which activate signature pathways and twisted metabolism in melanoma cells, contributing to melanoma progression. Targeting GCP‐2, its signaling, or senescent fibroblasts secreting GCP‐2 holds substantial promise for successfully counteracting melanoma progression in older adults.

## 2 Results

### 2.1 Accumulation of Senescent Skin Fibroblasts Correlates With Increasing Thickness of Primary Melanoma

**FIGURE 1:**
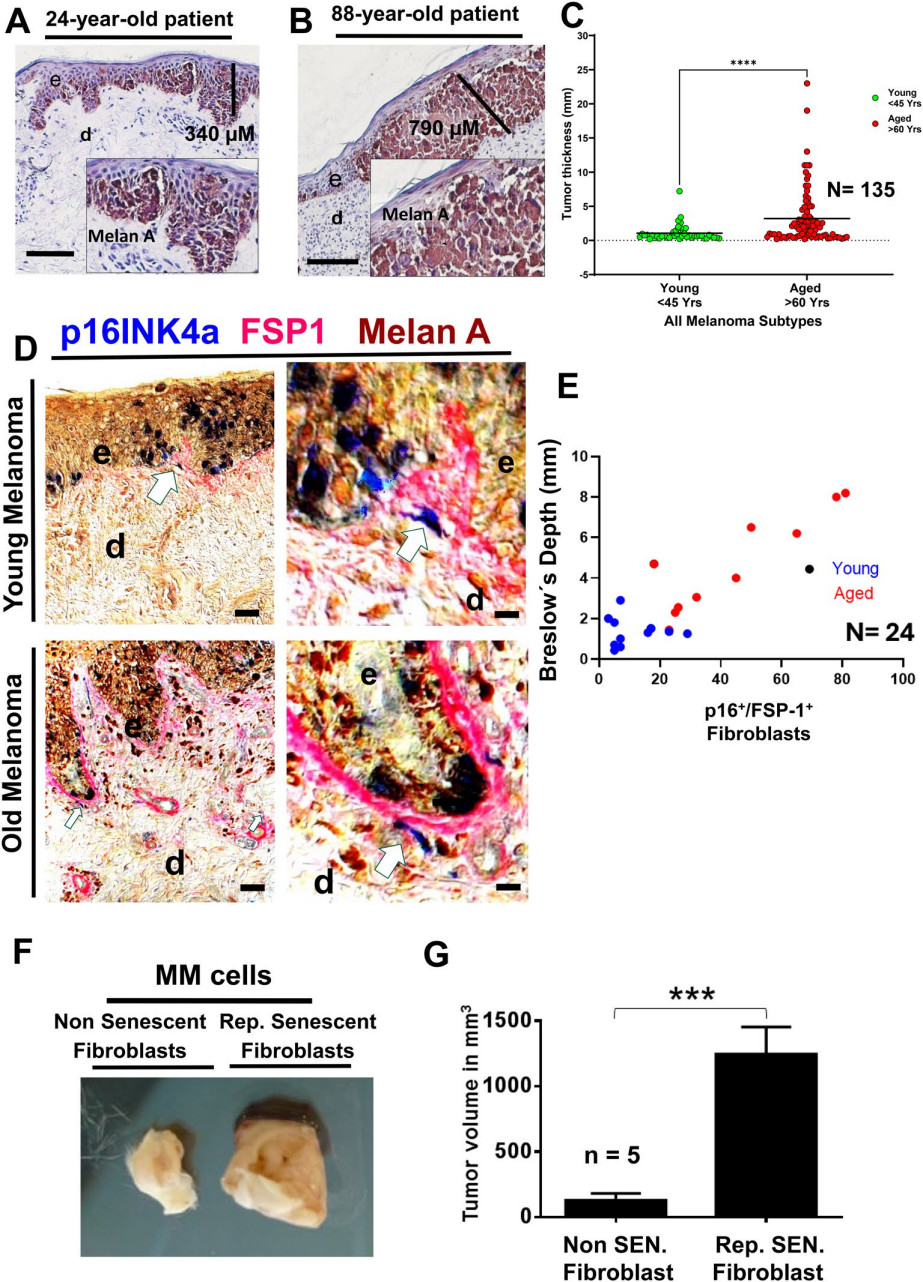
Accumulation of Senescent Fibroblasts Correlates with Melanoma Thickness (A) Representative photomicrographs of sections from a thin melanoma of a 24‐year‐old patient and (B) from a thick melanoma of an 88‐year‐old patient. Double staining was performed for Melan A, indicative of melanoma cells (brown) and hematoxylin for nuclei (blue). The vertical line indicates tumor depth, insets show cytological characteristics of melanoma cells with hyperchromatic, polymorphic nuclei at upper layers of the epidermis (e) and invading the dermis (d). Scale bars, 200 and 400 μm.(C) The correlation of melanoma thickness and patients´ age is shown, *n* = 135. ****p < 0.0004 by Mann–Whitney *U* test. (D) Representative photomicrographs of primary melanomas from a 24‐year‐old patient (top panel) and from an 88‐year‐old patient (bottom). Immunostaining of melanoma sections for the aging marker p16INK4a (blue) and Melan A (brown) depicting Melan A/p16INK4a‐positive melanoma cells (blue and brown overlay) and p16INK4A‐positive spindle‐shaped FSP‐1‐positive fibroblasts (blue and red overlay, as indicated by arrows) in the dermis. The antibodies against FSP‐1 (fibroblast‐specific antigen) detect fibroblasts and in part extracellular matrix secreted by fibroblasts. e, epidermis; d, dermis. The dashed line indicates the epidermal dermal junction. Scale bars, top and bottom panels 200 and 400 μm. (E) Biopsies of 24 young (< 45 years) and old adults (> 60 years) with melanoma of different subtypes and localizations were matched and sections of melanoma biopsies were stained for p16INK4a, a robust senescent marker, and for FSP‐1, a stromal fibroblast marker. Only double‐stained cells were counted indicative for senescent fibroblasts. p16+ fibroblasts and Breslow depth comparison depicts an almost linear correlation of *r* = 0.74 (upper right, panel). (F) 1 × 10^6^ human wild‐type A375 melanoma cells (were co injected with 1 × 10^6^ replicative senescent fibroblasts (REP SEN) and non‐senescent (NON SEN) fibroblasts into the skin of SCID mice. Replicative senescent (REP SEN) fibroblasts are devoid of any proliferative activity at a CPD of > 60. By contrast, non‐senescent (NON SEN) fibroblasts depict high proliferation activity at a CPD < 14. Tumors were explanted after 4 weeks of growth and documented by standardized photography. (G) The outer right diagram depicts tumor volumes of five tumors of each experimental group from (F), expressed as mean ± S.E.M. ***p < 0.0001 by Student's *t*‐test assuming that the data are unpaired and nonparametric and Mann–Whitney *U* test.

The overall thickness of primary malignant melanoma significantly increased with age in samples from 135 patients (Figure 1A–C and Table S1 and S2). If specified for subtypes, this correlation between age and melanoma thickness was also found in superficial spreading melanoma (Figure S1). This is of prime clinical relevance as the thickness of melanoma constitutes the most important intrinsic predictive factor for melanoma progression and subsequent morbidity and mortality. Of note, a significant increase in the number of p16^Ink4a+^/FSP‐1^+^ senescent fibroblasts (Strutz et al. 1995) was detected in close vicinity to malignant melanomas of old patients (> 60 years), but not of younger patients (< 45 years) (Figure 1D,E). Our data indicate that increased thickness of melanomas in older adults is associated with high numbers of p16^Ink4a+^/FSP‐1^+^ senescent fibroblasts adjacent to melanomas (Figure 1E). The previously reported p16^Ink4a^ expression in nevi, the benign counterpart of melanoma, and in malignant melanoma (Figure 1D) represents a tumor suppressor mechanism that, at later stages of melanoma progression, is bypassed by activating mutations (Michaloglou et al. 2005; Mooi and Peeper 2006). Consequently, among other factors, senescent fibroblasts in aged skin may critically contribute to the increased thickness and thus to the poor prognosis in older adults. This notion is further supported by our finding that injection of A375 melanoma cells together with replicative senescent fibroblasts into the skin of SCID mice produced significantly larger tumors after 4 weeks compared to A375 melanoma cells injected together with young, non‐senescent fibroblasts (Figure 1F,G).

### 2.2 GCP‐2 and ENA‐78 From Senescent Fibroblasts Enhance the Oncogenic Properties of Melanoma Cells In Vitro

We identified soluble factors, based on a multi‐pronged experimental approach, released from well‐characterized replicative senescent fibroblasts, which enforce melanoma progression (Figures S2–S5). In this regard, conditioned medium (CM) from p16^Ink4a+^ and BP53^+^ replicative senescent fibroblasts of two different fibroblast strains (for further characterization, see Figure S3) was studied. We found that CM from replicative senescent fibroblasts enhances key steps in melanoma progression (directed migration towards a gradient of chemokines and anchorage‐independent growth) of different primary melanoma as well as metastatic melanoma cells (Table S3). Among others, we detected a significant increase in granulocyte chemotactic protein‐2 (CXCL6/GCP‐2) and in the epithelial‐derived neutrophil activity peptide 78 (CXCL5/ENA‐78) in CM from replicative senescent fibroblasts (Figure S5A–D). Human GCP‐2 (Proost et al. 1993) exerts neutrophil chemotactic and angiogenic properties that are comparable to the action of interleukin‐8 (IL‐8)/CXCL8, growth‐regulating oncogene (Gro)‐α, β, у/CXCL1, 2, 3, and epithelial neutrophil‐activating peptide‐78 (ENA‐78/CXCL5) in neutrophil recruitment (Mei et al. 2010). The increase of distinct factors was further confirmed by specific ELISAs (Figure S5D) and immunostaining of young non‐senescent and replicative senescent cultured fibroblasts (Figure S6A). We also found that MACS isolated p16‐positive fibroblasts from the skin of old human adults (> 80 years old), when subjected to RNA seq analysis, revealed profoundly higher levels of GCP‐2 and ENA‐78 coding mRNA in senescent, but not in fibroblasts isolated from the skin of young adults (Figure S5E). These data indicate that replicative senescent fibroblasts closely mimic the release of the SASP factors like GCP‐2 and ENA‐78 of fibroblasts in vivo in the skin of old human adults. The finding that GCP‐2 and ENA‐78 are strongly expressed in vimentin‐positive spindle‐shaped fibroblasts (Figure S6B,C), but not in endothelial cells (Figure S6D, upper panel), and only to a non‐significant extent in leukocytes within the skin of older healthy individuals (Figure S6E, lower panel).

**FIGURE 2:**
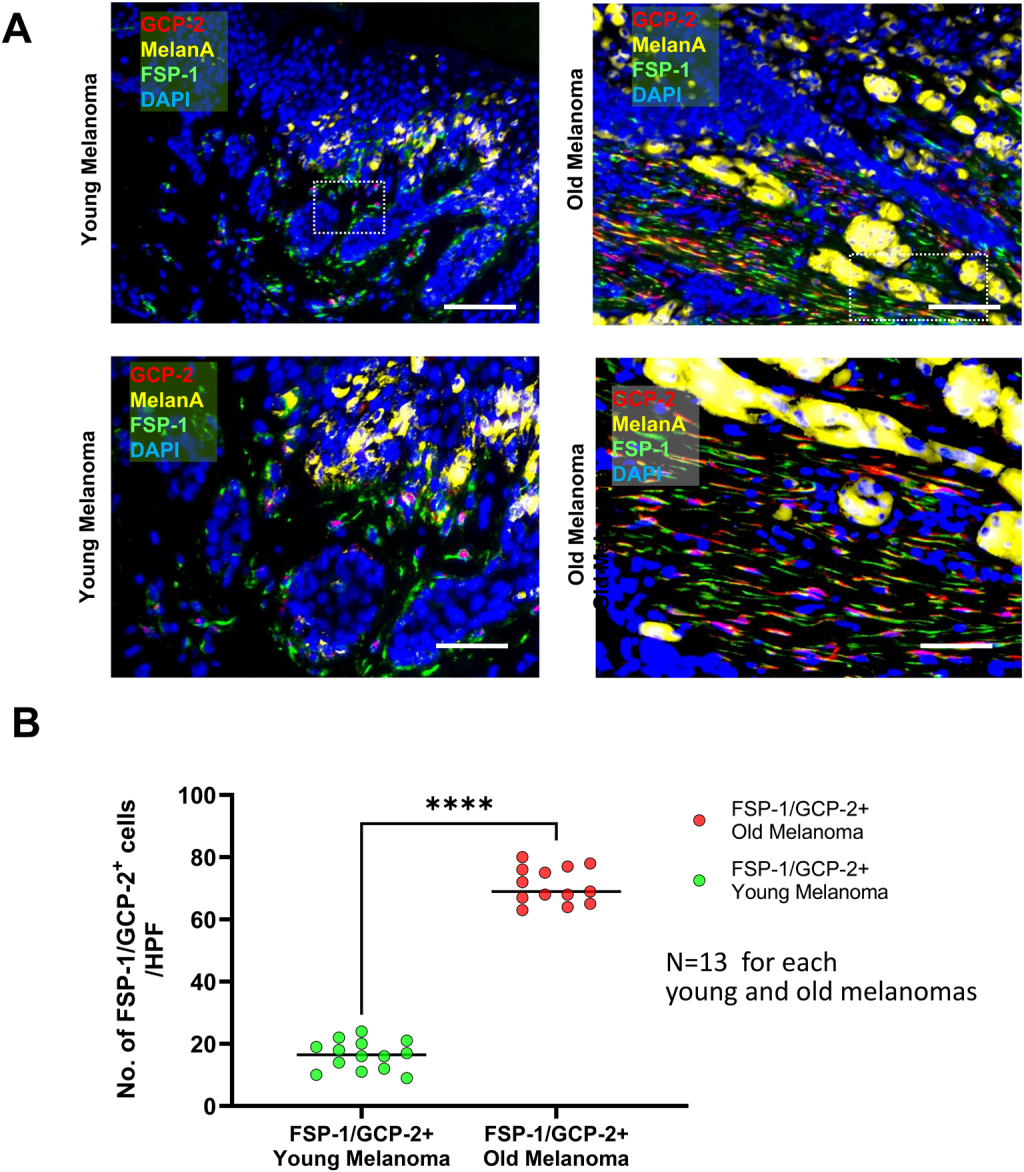
GCP‐2/FSP‐1 Double Staining Occurs in Peritumoral Fibroblasts of Melanoma from Old but not from Young Melanoma Patients. (A) Immunostaining of GCP‐2 (red), FSP‐1 (green), Melan A (yellow), and DAPI (blue) in melanoma sections of a young patients (left) and an old patient (right) from the cohort in (B). Spindle‐shaped FSP‐1‐positive fibroblasts (green) in old melanoma patients reveal enhanced GCP‐2 expression (red) compared to spindle FSP‐1‐positive cells (overlay (yellow to orange) compared to spindle‐shaped FSP‐1 positive fibroblasts without GCP‐2 expression of young melanoma patients (green). Scale bars, 10 and 100 μm. (B) Quantitative assessment of immunostainings of GCP‐2 expression in FSP‐1‐positive fibroblasts in melanomas from sections of 13 young melanoma patients (< 45 years) and 13 old melanoma patients (> 60 years). GCP‐2/FSP‐1 double‐positive cells were counted and presented as numbers/20× high power field). Data are mean ± S.E.M., *n* = 60 ****p < 0.0001 by Student's *t*‐test.

We found a high number of spindle‐shaped FSP‐1 positive fibroblasts in sections from a melanoma of a 82‐year‐old patient, while less numbers of FSP‐1 positive fibroblasts express GCP‐2 in sections of a 38‐year old young patient (Figure 2A). The sections from melanomas of these patients originate from a cohort of 13 young (< 45 years) and a cohort of 13 old melanoma patients (> 60 years) whose melanomas all immunostained for FSP‐1/GCP‐2 positive fibroblasts in these primary melanomas of old patients, but profoundly less in young patients (Figure 2B). These data highlight that GCP‐2 qualifies as SASP factor in replicative senescent fibroblasts, in fibroblasts in the skin of old adults (Figures S5E and S6B) and in in peritumoral fibroblasts of melanomas of old patients.

Directed migration of melanoma cells is a prerequisite for invasion into the host tissue and later melanoma cell dissemination. Recombinant human GCP‐2 and ENA‐78 resulted in a robust chemotactic response with enhanced directed migration of melanoma cells towards GCP‐2 and ENA‐78 (Figure S7A,B). Immunodepletion of either GCP‐2 or ENA‐78 from CM of senescent fibroblasts with neutralizing antibodies against GCP‐2 or ENA‐78 showed a significant concentration‐dependent inhibition of the migratory response of melanoma cells, with a maximum of 75% inhibition in the presence of GCP‐2 neutralizing antibodies (10 and 100 μg) (Figure S7C) and a maximum of 50% inhibition in the presence of ENA‐78 neutralizing antibodies (10 and 100 μg) (Figure S7D). By contrast, the isotype control antibody did not show any effect on CM‐induced migration (Figure S7E). The CXCR1 receptor binds ligands like GCP‐2, IL‐8, and NAP‐2 but not ENA‐78 and other chemokines, while the CXCR2 receptor binds to and confers the activities of both GCP‐2 and ENA‐78. Both receptors were expressed on melanoma cell lines (Varney et al. 2011) and our own data. As expected, SB225002, an inhibitor of CXCR1 and 2 receptors, or CM of senescent fibroblasts silenced for GCP‐2, significantly reduced the migratory response of A375 melanoma cells (Figures S7F). The migration of melanoma cells to CM from senescent fibroblasts which were silenced for GCP‐2 was almost complete, suppression while supplementation of CM from non‐senescent fibroblasts with rhGCP‐2 at concentrations released from replicative senescent fibroblasts enhanced directed melanoma migration (Figure S8A,B). We also studied anchorage‐independent growth. Physiologically, if cells are exposed to conditions where they are not able to attach to extracellular matrix proteins, they undergo anoikis, a specific form of apoptosis. Anchorage independence is a hallmark of cancer or melanoma cells and is distinctly required for invasion, dissemination, and metastasis. Interestingly, both recombinant GCP‐2 and, to a lesser extent, ENA‐78 stimulated anchorage‐independent growth of melanoma cells (Figure S9A,B), which was inhibited following either immunodepletion of GCP‐2, ENA‐78 (Figure S9C,D), or treatment with the CXCR1/2 inhibitor SB225005 (Figure S9E). These data suggest that GCP‐2 and, to a lesser extent, ENA‐78 constitute critical factors in CM from replicative senescent fibroblasts that, via CXCR1 and 2 signaling, promote melanoma cell migration and anchorage‐independent growth.

### 2.3 GCP‐2 but Not ENA‐78 Stimulates Melanoma Cell Growth In Vivo

**FIGURE 3:**
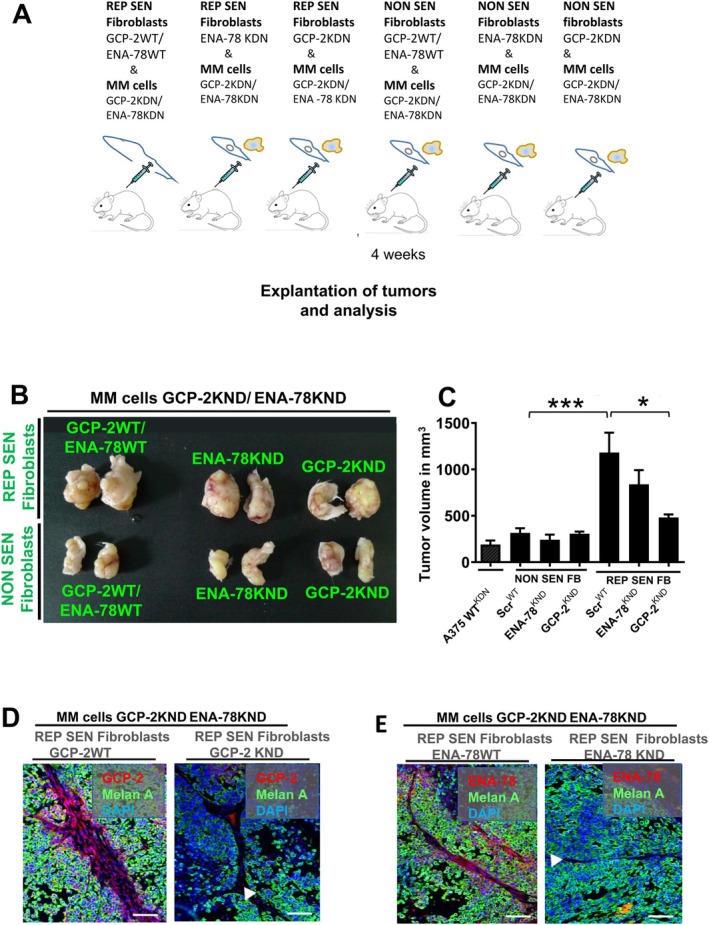
GCP‐2 and to a minor extent ENA‐78 Released by Replicative Senescent Fibroblasts Stimulates Melanoma Cell Growth in vivo. (A) Scheme depicting the experimental procedure. A375 melanoma cells silenced for GCP‐2 and ENA‐78 (MM cells GCP‐2KDN/ENA‐78KDN) were co‐injected subcutaneously either with replicative senescent (REP SEN) fibroblasts without any proliferative activity at a CPD of > 60, or young, non‐senescent (NON SEN) fibroblasts with high proliferation activity at a CPD < 14 of the indicated genotypes in SCID mice tumors for each experimental group were explanted after 4 weeks (left panel) and tumor volumes were assessed. (B) GCP‐2 and ENA‐78 expression was silenced in A375 melanoma cells (MM cells GCP‐2KDN/ENA‐78KDN). Melanomas grown to representative tumor volumes after previous co‐injection with replicative senescent (REP SEN) fibroblasts (upper left panel), with young, non‐senescent (NON SEN) fibroblasts (lower left panel), with replicative senescent fibroblasts (REP SEN) silenced for ENA‐78 (ENA‐78KDN, upper middle panel), with young fibroblasts (NON SEN) silenced for ENA‐78 (ENA‐78KDN, lower middle panel), with replicative senescent fibroblasts (REP SEN) silenced for GCP‐2 (GCP‐2KDN, upper right panel), and with young fibroblasts (NON SEN) silenced for GCP‐2 (GCP‐2KDN, lower right panel). The genotypes of co‐injected fibroblasts are depicted in green letters. (C) The outer right diagram depicts tumor volumes of five tumors of each experimental group from (B). Note that A 375 melanoma cells with silenced for GCP‐2/ENA‐78 (A375 WTKDN) served as control. Data are expressed as mean ± S.E.M. *p < 0.01, ***p < 0.0001 by one‐way ANOVA and Tukey's post hoc test. (D, E) Representative photomicrographs of tumors of the indicated genotypes stained for GCP‐2 (red) (D), ENA‐78 (red) (E), Melan A (green), and DAPI for nuclei (blue). GCP‐2 (D) and ENA‐78 (E) staining is exclusively detected in the connective tissue sheats containing senescent fibroblasts (Δ), but not in Melan A‐positive melanoma cells (green). Tumors developed from GCP‐2‐silenced replicative senescent (REP SEN) fibroblasts (D, right panel) or from ENA‐78‐silenced replicative senescent (REP SEN) fibroblasts (E, right panel) did neither show ENA‐78 nor GCP‐2 staining in the connective tissue sheats (Δ).

We next assessed the role of GCP‐2 and ENA‐78 on melanoma cell growth in vivo. GCP‐2‐silenced replicative senescent fibroblasts (Rep Sen FB, GCP‐2KDN), following co‐injection with A375 melanoma cells (which were silenced for GCP‐2 and ENA‐78), resulted in significantly smaller tumors, whereas ENA‐78‐silenced senescent fibroblasts (Rep Sen FB, ENA‐78KDN) only showed a tendency towards smaller tumors (Figure 3A–C). We have used A375 melanoma cells silenced for GCP‐2 and ENA‐78 to exclude any role of these autocrine chemokines released from melanoma cells on the overall melanoma size. Replicative senescent fibroblasts (GCP‐2WT/ENA‐78WT, green letters) in combination with such A375 melanoma cells (MM cells GCP‐2KDN/ENA‐78KDN) (Figure 3B,C), or WM‐266‐4 melanoma cells (Figure S10), resulted in an increased tumor size, like that of GCP‐2 and ENA‐78 competent wild type A375 melanoma cells (MM cells) (Figure 1F,G). No significant increase in tumor size was observed after co‐injection of A375 melanoma cells with replicative senescent fibroblasts silenced for ENA‐78. These data imply that ENA‐78 released from replicative senescent fibroblasts does not play any major role in enhancing melanoma size. Therefore, we focused in subsequent experiments on GCP‐2. Our data also demonstrate that GCP‐2 released from replicative senescent fibroblasts, but neither GCP‐2 nor ENA‐78 released from A375 melanoma cells, exerts a major effect on melanoma volume. To further confirm that GCP‐2 and ENA‐78 silencing in replicative senescent fibroblasts is reliably maintained in melanomas originating from the injection of replicative senescent fibroblasts together with A375^KDN^ melanoma cells into SCID mice, immunostaining for GCP‐2 or ENA‐78 staining was performed on sections of explanted melanomas. Interestingly, the connective tissue sheaths (**∆**) in these melanomas that contain GCP‐2‐silenced replicative senescent fibroblasts depicted a complete loss of GCP‐2 staining (red) (Figure 3D, right panel). A375 melanoma cells were stained green, indicative of melanoma‐specific Melan A. The lack of GCP‐2 staining in connective tissue streaks between melanoma cells contrasted with marked GCP‐2 staining (red) in explanted melanomas derived from the injection of non‐silenced replicative senescent fibroblasts (GCP‐2 WT) together with A375 melanoma cells (Figure 3D, left panel). Similar results were found for ENA‐78 staining (Figure 3E). These data indicate that silencing of GCP‐2 and ENA‐78 in replicative senescent fibroblasts was successful and maintained in vivo in the developing melanomas in SCID mice. GCP‐2 silencing in senescent and non‐senescent fibroblasts did not impact fibroblast proliferation or apoptosis. Importantly, co‐injecting A375 melanoma cells with non‐senescent fibroblasts, which were generated to overexpress GCP‐2, resulted in enhanced tumor volumes like those grown after injection with replicative senescent fibroblasts (Figure S11). In aggregate, these findings imply that GCP‐2 released from senescent fibroblasts is predominantly responsible for the significant increase in melanoma size.

### 2.4 GCP‐2 Induced CREB^S133^ Phosphorylation Drives Melanoma Progression

**FIGURE 4:**
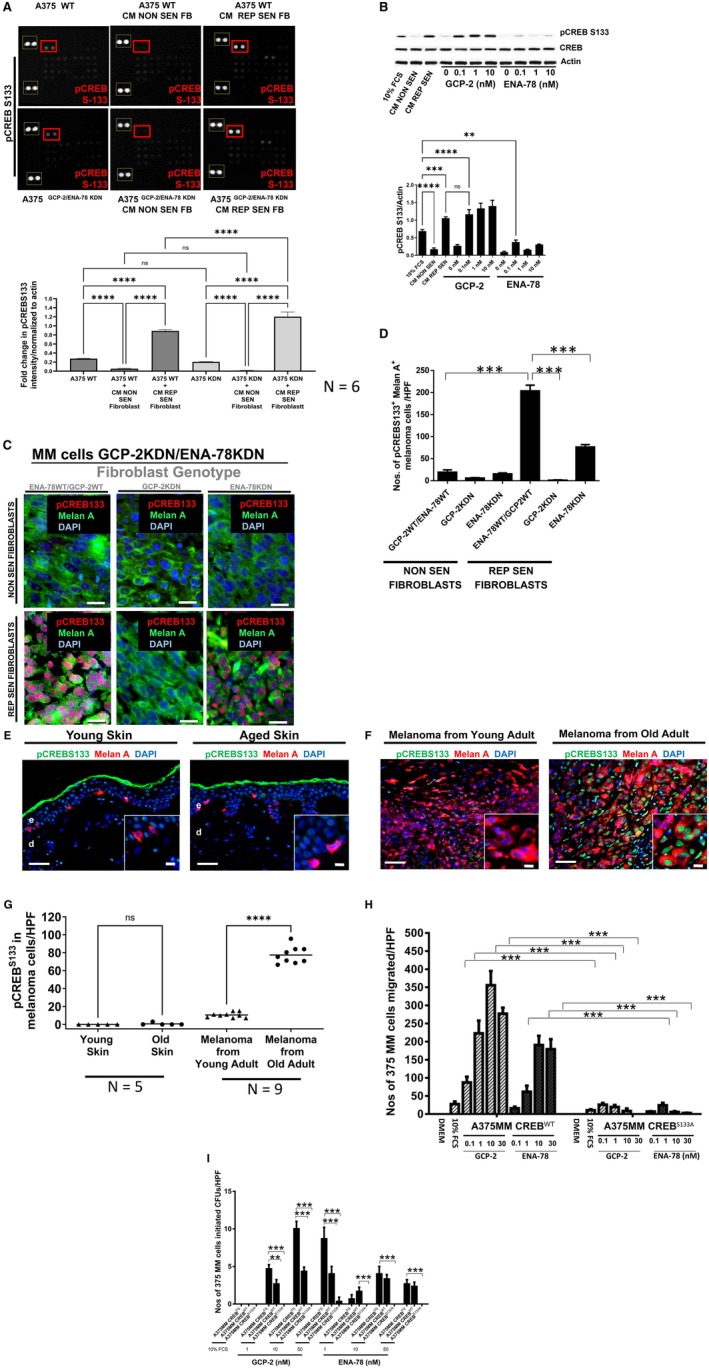
GCP‐2 Induced CREBS133 Phosphorylation Drives Melanoma Growth (A) CM from replicative senescent (CM REP SEN) but not from young non‐senescent fibroblasts (CM NON SEN) enhanced CREB phosphorylation in the melanoma cell line A375 as depicted by phosphokinase arrays (upper two panels). Note that the representative experiment of the upper panel was done with A375 wild‐type melanoma cells (A375 WT) and in the lower panel, we employed A375 melanoma cells silenced for GCP‐2/ENA‐78 (A375 KDN). CREB phosphorylation intensities are depicted in red rectangles. Actin served as loading control and is depicted in yellow rectangles. Data are mean ± S.E.M. *n* = 3. ns, nonsignificant, ****p < 0.0001 by ANOVA and Tukey's post hoc test (lower diagram). (B) Western blot analysis of CREB phosphorylation at the serine residue 133 (pCREBS133) in cell lysates from A375 melanoma cells exposed to CM from non‐senescent (CM NON‐SEN) or replicative senescent fibroblasts (CM REP SEN), and to recombinant GCP‐2 or ENA‐78 at the indicated concentrations (upper panel). Densitometry assessment of western blots with normalization of pCREBS133 to actin (lower panel). Data are mean ± S.E.M. *n* = 3. ns, nonsignificant, **p < 0.0001 by ANOVA, and Tukey's post hoc test. (C) Immunostaining of sections from tumors grown after co‐injection of A375 melanoma cells (MM cells GCP 2KDN/ENA‐78KDN) with replicative senescent (REP SEN FIBROBLASTS) and non‐senescent fibroblasts (NON SEN FIBROBLASTS) of different genotypes for phosphorylated CREB (pCREBS133, red) and DAPI staining nuclei (blue, overlay purple) of Melan A positive (green) A375GCP‐2KDN/ENA‐78KDN melanoma cells. (D) Quantification of CREBS133 Melan A‐positive cells of tumors derived from co‐injection of young, non‐senescent (NON SEN) and replicative senescent (REP SEN) fibroblasts of the indicated genotypes (*n* = 5) by multiple comparison ANOVA with Tukey's post hoc test, ***p < 0.0001. (E) Representative photomicrographs of skin sections of a healthy young (left) and an old individual (right) stained for Melan A (red) and phosphorylated (active) pCREBS133 (green). Nuclei were stained with DAPI (blue). A total of five young and five old individuals were studied. (F) Representative photomicrographs of melanoma sections of a 24‐year‐old patient (left) and a 84‐year‐old patient (right) stained for Melan A (red) and phosphorylated (active) pCREBS133 (green). Nuclei were stained with DAPI (blue). A total of nine melanomas from young patients and nine melanomas from old patients were studied. (G) The diagram depicts data of (F). Melan A pCREBS133‐positive melanoma cells were counted. Of note, only melanomas from old patients revealed increased CREB activation. Scale bar, 100 μM. At least sections of five different biopsies for each entity were analysed. All data are mean ± S.E.M. ***p < 0.001 by unpaired one‐way ANOVA multiple comparison with Tukey's post hoc test. (H) CREB with a functional phosphorylation site at serine 133 is essential for GCP‐2 and ENA‐78‐dependent melanoma cell migration. The migratory response of either A375 melanoma cells with wild‐type CREB (A375MM CREBWT) or with a replacement mutation at the CREB phosphorylation site at serine 133 (A375MM CREBS133A) was assessed. Mean ± S.E.M. All experiments were repeated three times. ***p < 0.0001 by ANOVA multiple comparison with Tukey's post hoc test. (I) CREB mutated at the phosphorylation site at serine 133 (CREBS133A) abrogates anchorage‐independent growth of A375 melanoma cells. Anchorage‐independent growth of either wild‐type CREB (CREBWT) or melanoma cells with a CREB mutation (A375 MM CREBS133A) were subjected to different concentrations of recombinant GCP‐2 and ENA‐78. The total number of A375 melanoma cells was counted following agar dissolution as indicated in the Materials and Methods section. SYBR green intercalation in the DNA double strand, indicative of viable cells, was quantified by the emission of fluorescence at the ratio of 485/520 nm. All data are the mean ± S.E.M. All experiments were repeated three times. **p < 0.001 by ANOVA multiple comparison with Tukey's post hoc test.

Phosphokinase arrays were used to study GCP‐2 and ENA‐78 downstream signaling events. Incubation of melanoma cells (A375 and WM‐266‐4) with CM from replicative senescent fibroblasts but not with CM from non‐senescent fibroblasts resulted in a significant induction of phosphorylation of the cAMP‐responsive element binding protein (CREB), a member of the basic leucine zipper (bZIP) family of transcription factors (Shaywitz and Greenberg 1999; Vinson et al. 1989) at serine 133 (Figure 4A, Figure S12A). This induction is independent of whether A375 melanoma cells are wild type for GCP‐2 and ENA‐78 (Figure 4A, upper panel) or whether they are silenced for GCP‐2 and ENA‐78 (Figure 4A, lower panel). CREB phosphorylation at serine 133 is required for its binding to the transcriptional co‐activator CREB‐binding protein (CBP). This results in CREB activation, nuclear translocation, and binding to cAMP‐responsive elements on the promoters of several genes involved in migration, matrix proteolysis, survival, and tumor progression (Braeuer et al. 2011; Melnikova et al. 2010; Shankar et al. 2005). Incubation of A375 melanoma cells with rhGCP‐2 (0.1–10 nM) and to a much lesser extent with rhENA‐78 led to CREB^S133^ phosphorylation (Figure 4B), and GCP‐2 enhanced translocation of phosphorylated CREB^S133^ to the nucleus of both non‐metastatic (WM‐115) and metastatic melanoma cells (WM‐266‐4), indicative of CREB activation (Figure S12B). To further test the relevance of GCP‐2 and ENA‐78 derived from replicative senescent fibroblasts on CREB phosphorylation in vivo, melanoma tumors established after subcutaneous injection of A375 melanoma cells with either non‐senescent or replicative senescent fibroblasts into SCID mice were analyzed for CREB^S133^ phosphorylation (Figure 4C). In these experiments, GCP‐2 and ENA‐78 were silenced in A375 melanoma cells to exclude a melanoma cell intrinsic effect on CREB phosphorylation. Following co‐injection of A375^KDN^ melanoma cells with non‐senescent fibroblasts, CREB^S133^ phosphorylation is virtually absent (Figure 4C, upper left panel) as opposed to a strong CREB^S133^ induction after co‐injection of A375^KDN^ melanoma cells with replicative senescent fibroblasts (REP SEN) (Figure 4C, bottom left panel). By contrast, when replicative senescent (REP SEN) fibroblasts silenced for GCP‐2 (GPC‐2KDN) were co‐injected with A375^KDN^ melanoma cells, CREB^S133^ induction is almost completely suppressed (Figure 4C, mid bottom panel and Figure 4D). Only a moderate suppression of CREB^S133^ phosphorylation was observed when ENA‐78‐silenced replicative senescent (Rep SEN) fibroblasts (ENA‐78KDN) were co‐injected (Figure 4C, right bottom panel and Figure 4D). These data suggest that primarily GCP‐2 secreted by senescent fibroblasts drives CREB^S133^ phosphorylation (activation) in vivo. Of note, virtually no CREB^S133^ phosphorylation occurred in melanocytes, the benign counterpart of melanoma cells, in healthy skin, independent of age (Figure 4E). Intriguingly, the expression of activated pCREB^S133^ (green) was markedly higher in DAPI‐stained nuclei (blue) of melanoma cells in older patients compared to young patients (Figure 4F,G). This increase in pCREB^S133^ is not due to an increase in the overall level of CREB, as non‐phosphorylated CREB did not increase in melanoma cells from old individuals (data not shown). GCP‐2 was overexpressed in Melan A‐negative fibroblasts (red, arrows) in the direct neighborhood of Melan A‐positive melanoma cells (yellow) of old patients (Figure 2A, right panel, arrows), but not of young patients (Figure 2A, left panel, arrows). This is highly consistent with a model in which GCP‐2 derived from senescent fibroblasts enhances pCREB S^133^ activation also in human melanoma. We also observed that senescent fibroblasts that released GCP‐2 enhanced proliferation and prevented melanoma cell apoptosis, most likely in a CREB‐dependent manner. In this regard, sections from explanted melanomas which developed after injection of melanoma cells together with GCP‐2 silenced replicative senescent fibroblasts into SCID mice showed completely suppressed CREB activation (Figure 4C, bottom middle panel) with reduced expression of the proliferation marker Ki67 (Figure S13A, outer right upper row, bottom panel, and Figure S13B) and enhanced expression of the apoptosis marker caspase 3 in melanoma cells (Figure S13A, lower row, outer right bottom panel, and Figure S13C). By contrast, caspase 3 and Ki 67 expression did not change significantly in melanomas that originate from co‐injection of A375 melanoma cells silenced for GCP‐2/ENA‐78 with non‐senescent fibroblasts of all assessed genotypes in SCID mice (Figure S13A–C). CREB activation is also mandatory for GCP‐2‐induced directed migration and anchorage‐independent growth of melanoma cells. This conclusion is supported by our results that GCP‐2 treatment of A375 melanoma cells, which harbor a mutation in the phosphorylation (activation) site of CREB, is neither able to generate a migratory response (Figure 4H) nor to grow in an anchorage‐independent manner (Figure 4l). Together, these data imply that GCP‐2 released from senescent fibroblasts in the peritumoral niche enforces prime hallmarks of melanoma progression.

### 2.5 CREB^S133^ Activation Shifts Melanoma Cell Metabolism to Glycolysis

**FIGURE 5:**
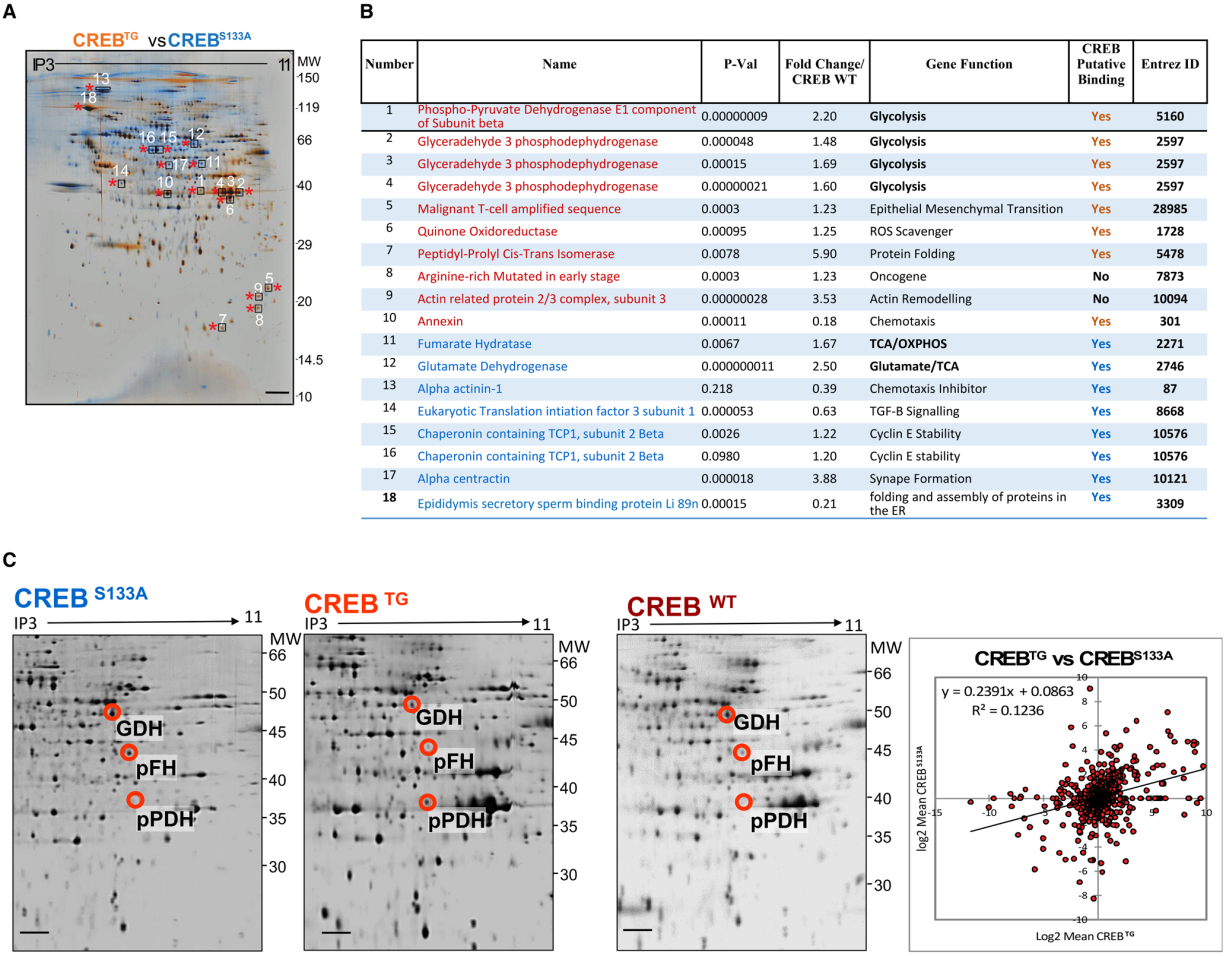
CREB Overexpression in Melanoma Cells Promotes Glycolysis at the Expense of TCA (A) Proteome analysis of the CREBTG A375 melanoma cell vs CREBS133A A375 melanoma cell phosphoproteins using 2D/MALDI‐TOF reveals a higher expression of TCA/OXPHOS enzymes in the CREB mutated melanoma cell group in comparison with the CREBTG group. Specifically, glutamate dehydrogenase (GDH), fumarate hydratase (FH), and chaperonin E increased in the CREBS133A melanoma cell group, while inactive phosphate dehydrogenase (phospho‐PDH1AE) and the glycolytic intermediate (glyceraldehye‐3 phosphodehydrogenase) are increased in CREBTG melanoma cells. (B) Data from (A) are depicted in the table according to fold change in CREB overexpressing A375 melanoma cells (CREBTG) compared with CREB wild‐type A375 melanoma cells. (C) A comparative representation of the 2‐dimensional protein spots is represented between CREB overexpressing (CREBTG), CREB mutated inactive (CREBS133A), and CREB wild‐type (CREBWT) A375 melanoma cells. The lower right panel depicts a correlation plot showing a total of identified spots (charge/size based) with low r values representing dissimilarities among both the CREBTG and CREBS133A groups normalized to CREBWT. Apart from the glycolytic genes that were upregulated in the CREBTG, ROS scavenging genes such as quinine oxidoreductase, genes coding for epithelial mesenchymal transformation such as MCTS1, or the oncogene ARMET were upregulated. Taken together, these results hint to a strong role of the transcription factor CREB in enhancing glycolytic enzymes, while suppressing TCA. Lower levels of glutamine dehydrogenase (GDH) as observed in CREBTG A375 melanoma cells suppress TCA cycle, while high GDH levels of the inactivated CREBS133A A375 melanoma cells enhance the TCA cycle by glutaminolysis resulting in higher α ketoglutarate supplies for the TCA cycle. Lower phosphorylated fumarate hydratase (FH), a key enzyme in TCA cycle likely supports the repressive effect of CREB activation on TCA cycle and tumor suppression as opposed to inactivated CREBS133A A375 melanoma cells with an enforced TCA cycle. Phosphorylation of PDH (pPDH) suppresses acetyl CoA formation as the key metabolite initiating the TCA cycle. Thus, high pPDH concentrations as detected by proteome analysis in CREBTG melanoma cells rather enhance glycolysis through a shift of pyruvate to lactic acid. All data are mean ± S.E.M., *n* = 3. *p < 0.01, ***p < 0.0001 by Student's *t*‐test.

To further explore target proteins downstream of CREB^S133^ activation and their role in melanoma progression, proteome analysis of CREB overexpressing (CREB^TG^) A375 melanoma cells versus CREB mutated (CREB^S133A^) A375 melanoma cells was performed employing 2D/MALDI‐TOF analysis. We identified a change in proteins involved in melanoma progression by their growth and proteins like proliferation inducing properties (peptidyl‐prolyl cis/trans isomerase (Pin1), the malignant T‐cell amplified sequence 1 (MTCS1)), by redox control (epididymis secretory sperm binding protein), and by the property to suppress apoptosis (ubiquinone oxidoreductase), among others in CREB overexpressing cells (Figure 5A,B). Most importantly, key proteins of metabolism were identified in CREB overexpressing melanoma cells, implying a shift towards energy sources like glycolysis upon CREB activation (Figure 5). In fact, CREB overexpressing A375 melanoma cells showed a profound upregulation of phosphorylated phospho‐pyruvate dehydrogenase (pPDH). PDH in its phosphorylated form is inactive and cannot enforce the conversion of pyruvate to acetyl CoA, the main substrate that drives the tricarboxylic acid (TCA) cycle. Hence, the TCA cycle is profoundly suppressed in CREB overexpressing A375 melanoma cells, while glycolysis is enhanced, and this is associated with a substantial increase in melanoma progression in vitro and in vivo. Conversely, a severe reduction of pPDH was found in the CREB^S133A^ mutated A375 melanoma cells, promoting the TCA cycle (Figure 5A–C). Glutamate dehydrogenase (GDH), a cytosolic enzyme that generates α‐ketoglutarate, an important substrate for the TCA cycle, is significantly reduced in CREB overexpressing A375 melanoma cells (Figure 5C). By contrast, in A375 melanoma cells overexpressing mutated CREB (CREB^S133A^), GDH was increased (Figure 5C). Previously unreported, fumarate hydratase, which converts fumarate to malate, driving the TCA cycle, was reduced in CREB overexpressing, while significantly increased in CREB^S133A^ mutated A375 melanoma cells.

**FIGURE 6:**
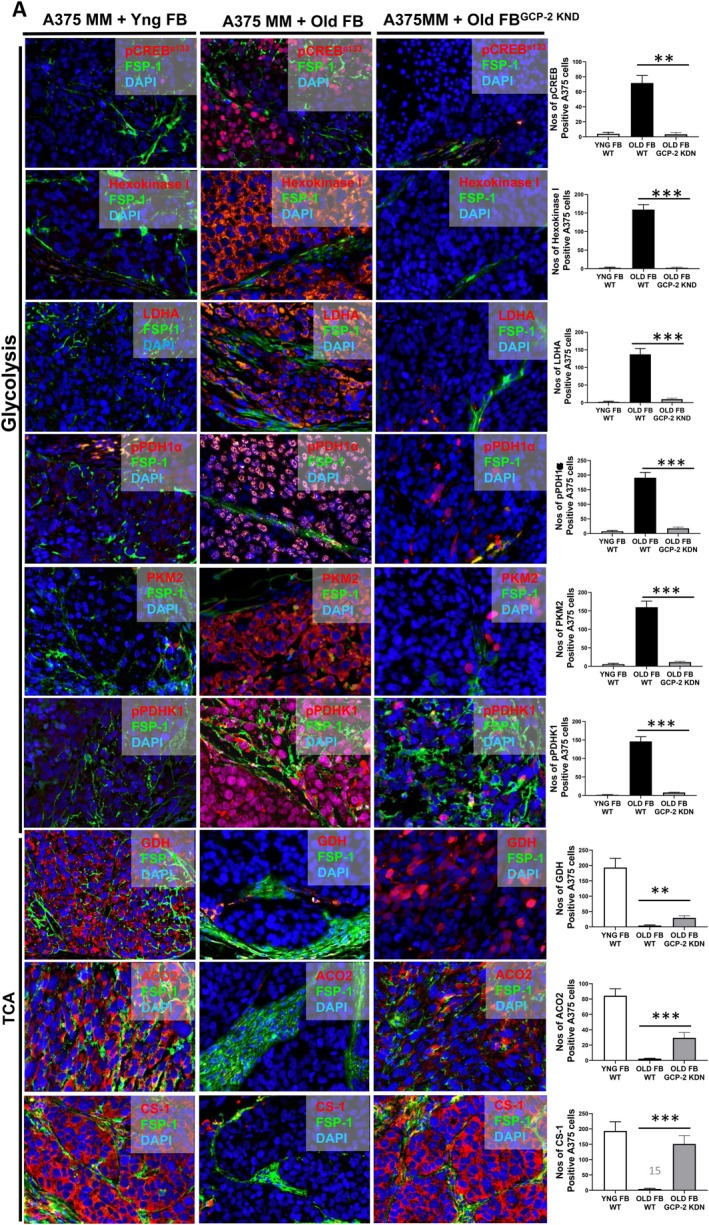
GCP‐2 released from senescent fibroblasts via CREB activation shifts metabolism toward glycolysis in melanoma cells. Representative microphotographs of sections from melanomas established in SCID mice following co‐injection of A375 melanoma cells with either. With non‐senescent (young) fibroblasts (Yng FB) or replicative senescent fibroblasts (Old FB) or with replicative senescent fibroblasts silenced for GCP‐2 (Old FBGCP‐2 KDN). Immunostaining was performed for FSP‐1, a fibroblast‐specific protein 1 (green), nuclei were stained with DAPI (blue). Extensive staining for activated CREB (pCREBS133, red) was observed in melanomas originating from co‐injection with replicative senescent (Old FB) and A375 melanoma cells into SCID mice. pCREBS133 staining was almost completely suppressed in melanomas when originating from co‐injection of melanoma cells with GCP‐2‐silenced replicative senescent (old) fibroblasts. Glycolytic and TCA cycle enzymes are indicated at the outer left side of the panels (glycolysis, TCA). Immunostaining against glycolytic enzymes include Hexokinase I (red), inactivated phospho‐Pyruvat‐Deydrogenase (pPDH1α, red), Lactate Dehydrogenase A (LDHA, red), Pyruvate Kinase isoenzyme M2 (PKM2, red), and Pyruvate Dehydrogenase Kinase1 (PDHK1). Immunostaining was also performed for the TCA indicator enzyme Glutamate Dehydrogenase (GDH, red), Aconitase (ACO2, red), and Citrate Synthase (CS, red). Of note, melanomas established after co‐injection of melanoma cells with replicative senescent (old) fibroblasts depict increased expression of glycolytic enzymes. The expression of glycolytic enzymes was abrogated in melanomas derived following co‐injection of melanoma cells with replicative senescent fibroblasts silenced for GCP‐2 (SEN FBGCP‐2KDN). Almost no expression of the TCA enzymes GDH, ACO2, and CS was observed in melanomas established from melanoma cells with replicative senescent fibroblasts, while GDH was highly expressed in melanomas derived after injection with replicative senescent (old) fibroblasts silenced for GCP‐2. These data imply that GCP‐2 released from replicative senescent fibroblasts is responsible for the induction of glycolytic enzymes, while suppressed GDH is indicative for reduced TCA cycle. Data were represented as positive cells/total cell count. The graphical representation of the ratio of positively stained cells and the total cell number is shown in the bar chart (outer right panel). All data are mean ± S.E.M. *n* = 3. **p < 0.01, ***p < 0.0001, ****p < 0.0001 by Student's *t*‐test.

**FIGURE 7:**
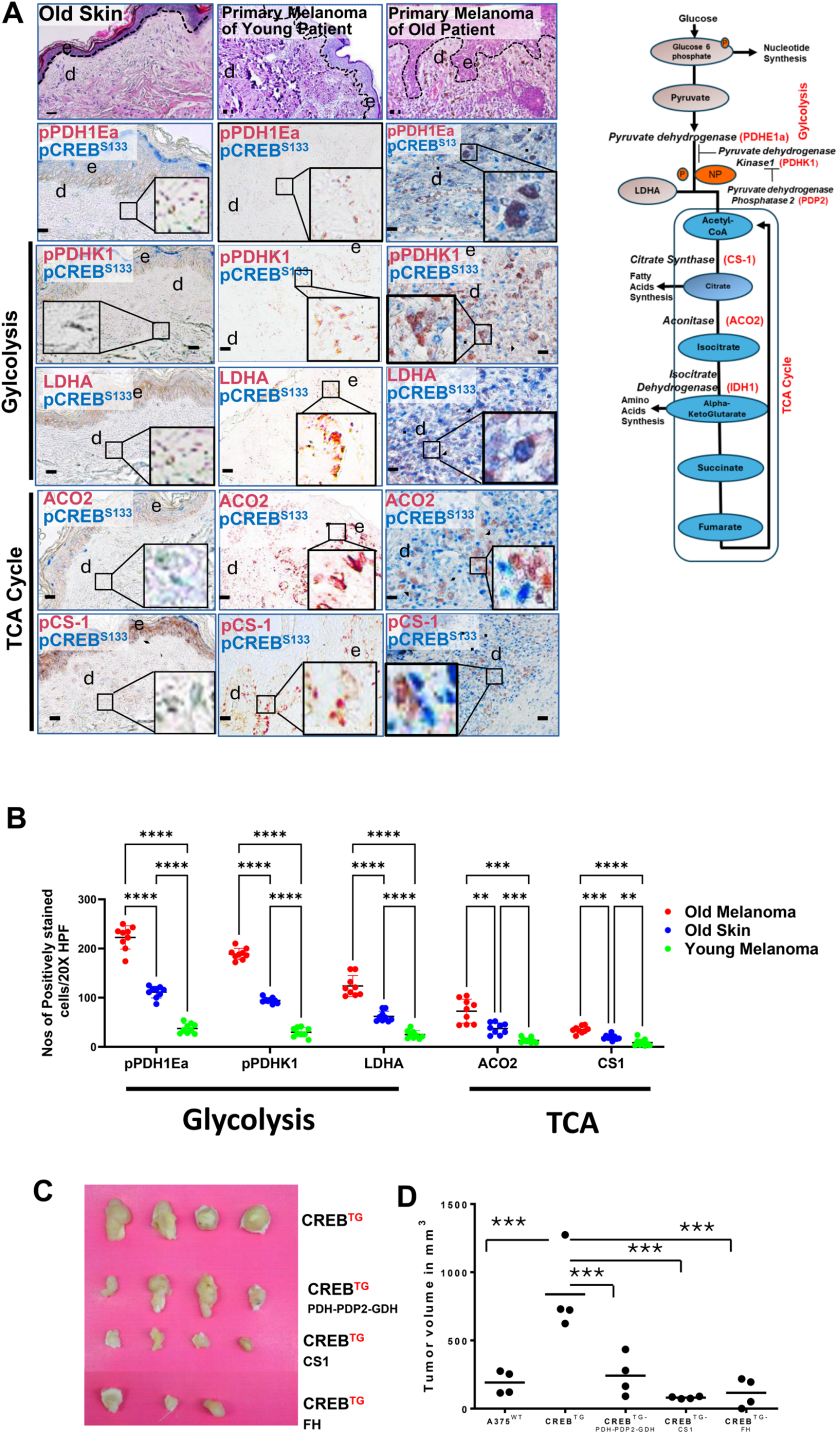
CREBS133 Phosphorylation Promotes Melanoma by Enhancing Glycolysis. (A) Representative photomicrographs of skin sections of a healthy 84‐year‐old individual (left panel), a primary melanoma of a 16‐year‐old young patient with superficial spreading melanoma (middle panel), and a 73‐year‐old patient with superficial malignant melanoma (right panel) stained for H&E (upper row) and below for activated (phosphorylated) CREB at S133 (pCREBS133, blue) and various enzymes of glycolysis and the TCA cycle (brown) using immunostaining. The relationship of the studied enzymes to the glycolytic or the TCA metabolic pathways and the employed abbreviations are depicted in the graphical sketch (outer right graph). An overview and higher magnification (inset) are depicted. The selected area for high magnification is depicted by a small box connected with black lines to the area of higher magnification. CREB phosphorylation (activation) was mainly found in skin sections of primary melanomas of old patients but not in young patients, while in normal skin from old adults, minor staining was only observed in higher layers of the epidermis, which do not correspond to melanonocytes confined to the epidermal dermal junction (stippled lines). Of note, double staining of pCREB+ melanoma cells (blue at the nucleus indicative of activated CREB) with glycolytic enzymes (brown) is much stronger in sections from old melanoma patients compared to almost no double staining of CREB and TCA cycle enzymes (brown) in sections from young melanoma patients. Immunostainings are representative of nine independently studied healthy human skin biopsies and for nine superficial spreading melanomas of young patients (age ranging from 27 to 37 years) and old patients (age ranging from 67 to 89 years). Melanomas from young and old melanomas are matched for anatomical localization. e, epidermis; d, dermis; dashed line, epidermal dermal junction. Scale bars 200 μM in case of old skin sections and 400 μM in case of primary melanomas. (B) Quantification of cells double stained for nuclear CREB and metabolic glycolytic or TCA enzymes from nine samples of skin from old healthy adults, nine melanomas from old adults, and nine samples from melanomas from young patients are depicted. Double staining (nuclear CREB and metabolic enzymes as indicated at the *y*‐axis) are depicted for melanoma cells in melanoma specimen and for healthy old adults only epidermal cells depicting a staining were counted and expressed as numbers of double‐positive stained cells/per high power field (HPF). Double staining for CREB occurred only with glycolytic enzymes but not for TCA enzymes in melanomas from old adults. Virtually, no CREB staining occurred in young melanomas or in spindle cells in the dermis. Virtually, no CREB staining was detected in the nucleus in cells confined to the epidermal dermal junction where melanocytes reside. Only few epidermal cells depict a CREB and a metabolic enzyme staining. ***p < 0.0001 by ANOVA multiple comparison with Tukey's post hoc test. (C) To understand whether an enforced switch from glycolysis towards the TCA cycle may abrogate the growth of transplanted CREB‐activated A375 melanoma cells (CREBTG), we injected CREBTG A375 melanoma cells, which overexpress the combined cDNAs coding for pyruvate dehydrogenase (PDH), the gate keeper enzyme for TCA initiation, its activating dephosphorylating phosphatase (PDP2), and glutamine dehydrogenase (GDH) (PDH‐PDP2‐GDH) into SCID mice. GDH in addition to the TCA cycle promoting PDH and PDP2 enforces TCA cycle by its capacity to enhance α ketoglutarate concentrations. In addition, the cDNA coding for the key TCA enzyme citrate synthase (CS1), which metabolizes acetyl CoA irreversibly in a one‐way direction to citrate, thus effectively driving TCA cycle, and the cDNA for fumarate hydrogenase (FH), an important TCA cycle enforcing enzyme, were overexpressed in CREBTG A375 melanoma cells. Representative pictures of explanted CREBTG A375 melanoma cells overexpressing key TCA enzymes in particular CS1 and FH led to a significant size reduction of melanomas after injection of CREB activated melanoma cells (CREBTG) in SCID mice. (D) Data from (C) are depicted. Tumor volumes of four tumors for each genotype were analyzed (right diagram). ***p < 0.0001 by ANOVA multiple comparison with Tukey´s post hoc test.

Similarly, melanoma derived from co‐injection of replicative senescent fibroblasts with A375 melanoma cells into SCID mice is larger and reveals a consistent overexpression of activated CREB and glycolytic enzymes, including Hexokinase 1, Lactate Dehydrogenase A (LDHA), Pyruvate Kinase isoenzyme M2 (PKM2), while depicting reduced expression of Glutamate Dehydrogenase (GDH), a key enzyme of the TCA cycle. CREB activation with enhanced expression of glycolytic enzymes was profoundly reduced in melanomas originating from co‐injection of melanoma cells with replicative senescent (Rep SEN) fibroblasts silenced for GCP‐2 (Figure 6). These data imply that GCP‐2 released from replicative senescent fibroblasts is mainly responsible for the CREB‐mediated up‐regulation of the glycolytic state in melanoma cells (Figure 6). Interestingly, a representative photo‐micrograph of biopsies derived from primary melanomas of old patients depict enhanced CREB expression (Figure 7A). By contrast, virtually no double staining of pCREB and either citrate synthase (CS) or aconitase (ACO2), key enzymes in the TCA cycle, was detected in sections from old patients with human primary melanomas (Figure 7A, B). Unbiased mRNA profiling of A375 melanoma cells overexpressing active CREB (CREB^TG^) cells depicts high expression of transcripts clustering in the glycolysis pathway, with suppressed transcripts coding for proteins in the TCA cycle. By contrast, A375 melanoma cells harboring a plasmid expressing an inactivating mutation of CREB show the opposite, with highly expressed genes of the TCA cycle (Figure S14A). In addition, there is a shift from ketogenic amino acids originating from the TCA cycle towards glucogenic amino acids originating from glycolysis (data not shown). Employing U‐13C6 glucose stable isotope tracing analysis in A375 melanoma cells exposed to GCP‐2, we found a profound metabolic shift towards glycolysis (Figure S14B).

To functionally assess metabolic expression profiles, real‐time mitochondrial oxygen consumption (respiration) indicative of OXPHOS/TCA (Figure S15A,B upper panels) and extracellular acidification rates indicative of glycolysis (Figure S15A,B lower panels) were performed employing the Seahorse extracellular flux analyzer. Here, we found that CREB activation, in fact, resulted in higher extracellular acidification rates (ECAR), indicative of glycolysis as opposed to enhanced TCA resulting from CREB inactivation either by overexpression of mutated CREB in A375 melanoma cells or by employing an inhibitor of CREB activation. Of note, GCP‐2 was able to enhance ECAR while suppressing respiration (OXPHOS/TCA) (Figure S15A,B). These data show that the GCP‐2 CREB activation axis profoundly contributes to the melanoma‐promoting “glycolytic state” (also referred to as “Warburg‐like effect”), while suppressing the “oxidative state.” These data, in addition, provide strong evidence for the impact of peritumoral senescent fibroblasts driving melanoma progression and clearly uncover CREB activation as a major switch for metabolic transformation towards neoplastic progression.

### 2.6 Forced Overexpression of TCA Enzymes or CREB Inhibition Suppresses the Glycolytic Shift and Melanoma Progression

To assess the causal role of TCA enzymes on melanoma growth, we overexpressed TCA enzymes along with CREB in A375 melanoma cells. For this purpose, we co‐overexpressed three interrelated TCA cycle driving enzymes, which act together in a functional TCA cycle hub (Figure 7C,D). PDH, the gatekeeper enzyme for pyruvate conversion to the TCA cycle, required acetyl‐CoA, and was overexpressed in conjunction with PDH phosphatase 2 (PDP2), which dephosphorylates and, thus, activates PDH, driving the TCA cycle. The third TCA cycle enhancing enzyme, which, together with PDH and PDHP2, was overexpressed in A375 melanoma cells, is glutamine dehydrogenase (GDH). GDH prevents α‐ketoglutarate from leaking out of the TCA cycle and, in consequence, protects it from being alternatively channeled into amino acid synthesis instead (see graphical sketch Figure 7 upper outer right panel). The combination of these TCA supporting enzymes was purposely selected for overexpression in CREB transgenic A375 melanoma cells, which were then injected subcutaneously in a murine transplantation model. Expression of these TCA‐enhancing enzymes in CREB transgenic melanomas (CREB^TG^/PDH‐PDP2‐GDH) resulted in significantly reduced melanoma size when compared to melanomas developed from CREB overexpressing melanoma cells CREB^TG^ (Figure 7C,D). Citrate synthase (CS) is responsible for the irreversible conversion of acetyl‐CoA to citrate, crucial for the TCA cycle. Overexpression of CS in CREB overexpressing A375 melanoma cells (CREB^TG^/CS) suppressed the melanoma growth in the transplant mouse model. In addition, overexpression of a TCA‐promoting gene, fumarate hydratase (FH), in CREB overexpressing melanoma cells (CREB^TG^/FH), resulted in an impressive reduction in melanoma size in comparison to melanomas that developed after injection of CREB^TG^ A375 melanoma cells (Figure 7C,D). These data support our hypothesis that enforced TCA in melanoma cells results in suppression of melanoma growth. Interestingly, apart from melanoma suppression, overexpression of the studied combinations of TCA enhancing enzymes (PDH‐PDP‐GDH) or the employment of a CREB inhibitor (CREBi) led to an almost complete restoration of the phospho‐protein pattern of activated TCA enzymes (data not shown), increased TCA metabolites, and resulted in a profound suppression of glycolysis (data not shown), demonstrating that fueling of the TCA cycle and not the toxicity of accumulating substrates are responsible for our observations.

### 2.7 Metabolome Profiling Uncovers a Shift Towards Glycolysis in Melanomas From Old Patients

**FIGURE 8:**
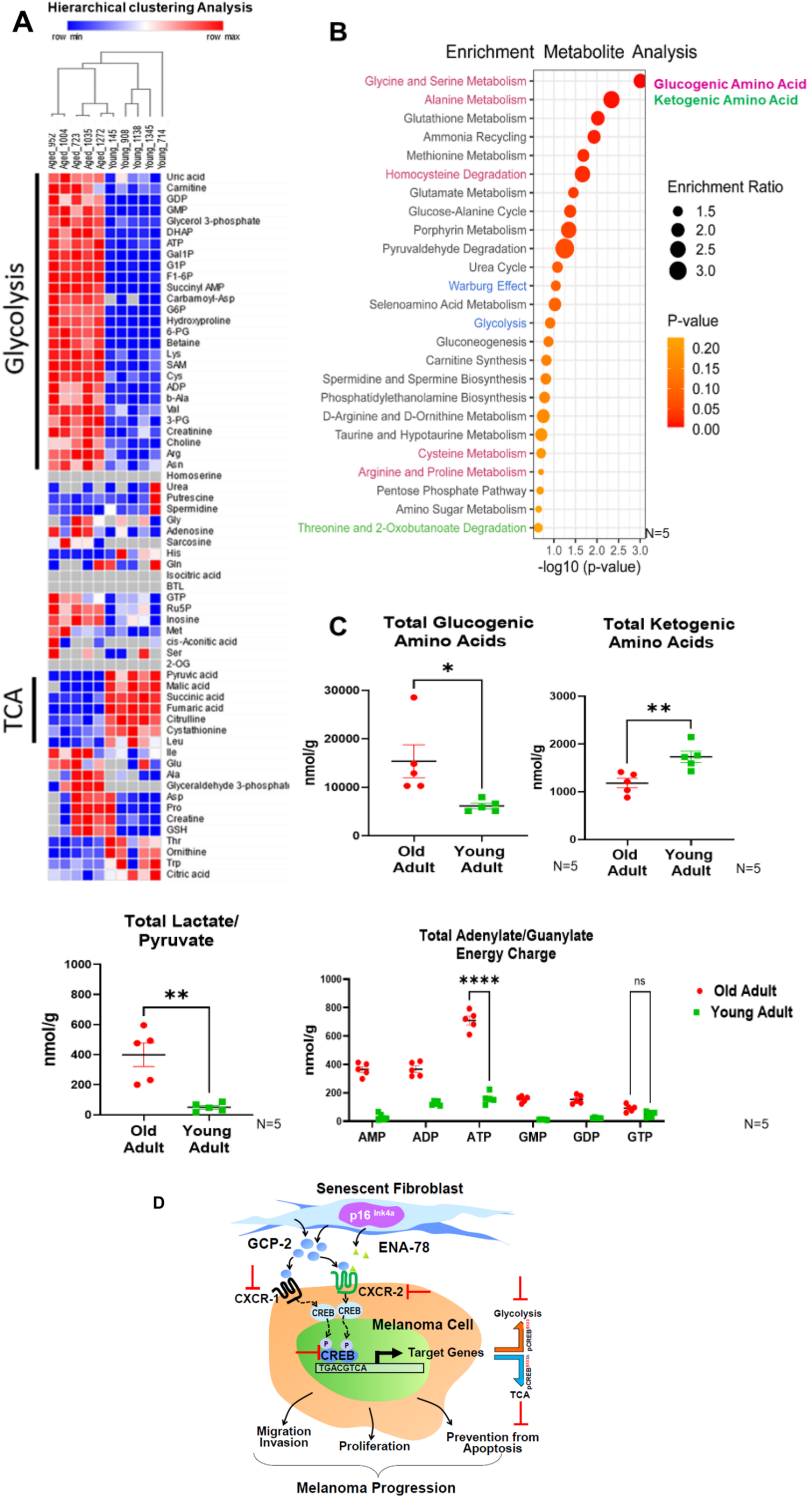
Metabolome Profiling Uncovers a Shift Toward Glycolysis in Melanomas from Old Patients. Metabolome analysis was performed on biopsy samples from superficial spreading melanomas from five young and five old melanoma patients. The locations of biopsies from young and old melanoma patients were matched (young patients: back (2), lower leg (1), upper trunk (2); old patients: back (2), lower leg (1), abdomen (1), upper trunk (1)). The age range of the young melanoma patients ranged from 22 to 44 years of age, that of old patients from 66 to 86 years of age. (A) Differentially regulated intermediate metabolites of glycolysis and TCA are shown in the hierarchical clustering analysis indicated as heat‐map charts (left). The upregulated metabolites are depicted in red color and the downregulated metabolites are shown in blue. Those metabolites that are unchanged are depicted in gray to white color, which is common in both the age groups. A significant large number of glycolytic metabolites are expressed in a high proportion in melanomas of old adults as compared to melanomas from young patients. In young patients melanomas, glycolytic intermediates are poorly expressed and TCA metabolites are expressed at high concentrations. (B) Enrichment analysis plot of the metabolites shows the enrichment ratio between biopsies from old vs biopsies from young melanoma patients along the *y*‐axis. The enrichment ratio scale (from 1.5‐folds to threefold) is represented by the size of the circles and the significance as *p*‐value along the *x*‐axis. Melanomas from old patients show amino acid metabolism by‐products with significant higher levels (more than twofold) of glycine, serine, cysteine, homocysteine, proline, and arginine; these amino acids are indicative of glucogenic amino acids representing glycolysis pathway (shown in pink). The ketogenic amino acids preferentially deriving from TCA (in green) were detected at lower level (< 1.5‐fold) in melanomas from old patients. Threonine, alpha‐ketoglutarate (shown in green). (C) The total glucogenic and ketogenic amino acids metabolites in melanomas from old patients (red) and in melanomas from young patients (green) are depicted. In melanomas from old patients, higher glucogenic and lower ketogenic amino acid metabolites occurred as opposed to melanomas from young patients. The total lactate to pyruvate ratio is also higher in melanomas from old patients as opposed to melanomas from young patients. The total ATP/GTP ratios are higher in melanomas from old patients when compared to melanomas from young patients. The *y*‐axis represents metabolite trace amounts in nmol/g and *x*‐axis as groups. All experiments were technically repeated three times with five different melanomas from old adults and five melanomas from young adults. *p < 0.0001 by ANOVA multiple comparison with Tukey's post hoc test. (D) Summary graph. Senescent Fibroblasts Drive Melanoma Progression. Senescent p16Ink4a+ fibroblasts release enhanced concentrations of GCP‐2/CXCL‐6 and ENA‐74/CXCL 5, which bind to their corresponding receptors. GCP‐2/CXCL‐6 significantly more than ENA 74/CXCL‐5 enhances CREB phosphorylation (activation) and its location from the cytoplasm to the nucleus. In the nucleus, it binds to its specific binding sites and transactivates the expression of target genes. These genes promote migration, anchorage‐independent growth, suppression of apoptosis and oxidative stress. Most importantly, CREB activation leads to a metabolic shift toward glycolysis at the expense of TCA cycle. Currently available inhibitors of CCRX‐1 and CCRX‐2 receptors as well as CREB inhibitors and forced expression of TCA enzymes hold substantial promise to qualify as effective therapeutic strategies to counteract neoplastic melanoma progression in old patients.

To investigate whether a shift towards glycolysis also occurs in melanomas of patients at old age, as opposed to no shift in young melanoma patients, we subjected melanoma tissue of patients of different ages to metabolome analysis. Melanomas from old patients showed indeed a clear shift towards glycolysis (hierarchical clustering analysis) with a strong increase in intermediate metabolites of glycolysis and, at the same time, a decrease of TCA metabolites (Figure 8A,B). In contrast, glycolytic intermediates are poorly expressed, and TCA metabolites are detected at high concentrations in melanomas from young patients. Interestingly, melanomas from old patients show amino acid metabolism byproducts with significantly higher levels of glucogenic amino acids, indicative of glycolysis and low concentrations of ketogenic amino acids, enforcing TCA. The total lactate to pyruvate ratio and ATP levels are also higher in melanoma biopsies from old patients (Figure 8C). In aggregate, these data support a view in which CREB activation in melanoma cells from old patients causes a switch of metabolism towards glycolysis, at the expense of the TCA cycle.

## 3 Discussion

Our major finding is that senescent fibroblasts in aged skin—via the soluble chemokine GCP‐2—activate the master transcription factor CREB in melanoma cells and, in consequence, suppress apoptosis, enhance migration, and melanoma growth due to a switch towards glycolysis (see graphic summary, Figure 8D). Our data further support the emerging concept that cues from the peritumoral microenvironment significantly impact melanoma and, in general, tumor progression (Krtolica et al. 2001; Landsberg et al. 2012; Ruhland et al. 2016).

CREB activation has been reported to be associated with a variety of human cancers (Chhabra et al. 2007; Crans‐Vargas et al. 2002; Seo et al. 2008), and a CREB regulon has previously been studied in the PC12 neuroblastic cell line (Impey et al. 2004), without functional analyses of CREB target genes in the context of metabolic reprogramming or tumor progression. Our results fundamentally extend previous in vitro *data* concerning the role of CREB *in* melanoma (Melnikova et al. 2010; Shankar et al. 2005). In addition to the newly uncovered link between senescent fibroblasts, which GCP‐2‐dependently activates CREB through phosphorylation at S133 in melanoma cells, we also identified the previously unreported role of the transcription factor CREB in regulating major metabolic pathways, and its impact on enhancing glycolysis. CREB, thus, qualifies as an essential metabolic regulator of melanoma. The advantage of tumor cells to rely on glycolysis, suggested by Otto Warburg already in 1956, was later confirmed as a hallmark for malignancy in general (Hanahan and Weinberg 2011). Though glycolysis has recently been claimed to play a prime role in melanoma progression (Bettum et al. 2015; Haq 2014; Kaplon et al. 2013; Roesch et al. 2013; Tasdogan et al. 2020), the impact of the peritumoral environment on melanoma metabolism is largely unexplored. Of note, primary melanomas in old patients revealed an impressive co‐staining of activated CREB, which typically translocates to the nucleus, and glycolytic enzymes in the cytoplasm of melanoma cells. These data show that our findings likely also apply to human melanoma.

Earlier, it was suggested that the oncogenic BRAF^V600E^ mutation is responsible for oncogene‐induced senescence (OIS), and that this oncogene acts as a brake protecting melanocytes from malignant transformation to melanoma (Kaplon et al. 2013). The authors provide evidence that the mitochondrial gatekeeper pyruvate dehydrogenase (PDH) is crucial for the BRAF^V600E^‐induced OIS. PDH, in fact, drives TCA and enhances the generation of reactive oxygen species responsible for OIS. This metabolic state is considered a tumor suppressor mechanism as it promotes melanoma cell senescence. We here show that GCP‐2 enhanced CREB activation—opposite to OIS—distinctly enhances melanoma cell proliferation by enforcing a metabolic shift towards a melanoma progression “glycolytic state.”

The question of how a mutation at the phosphorylation site and, in consequence, CREB inactivation regulates the switch from glycolysis to TCA/OXPHOS metabolism needs to be further investigated. It is, however, likely that this profound transcriptomic shift is due to the inability of CREB mutated at the phosphorylation site either to translocate to the nucleus or to exert its function as a repressing transcription factor (Lamph et al. 1990), thereby suppressing glycolysis‐encoding genes while enforcing TCA cycle‐enhancing genes.

With the employed xenotransplantation model (SCID mice with human fibroblasts and melanoma cells), we had the unique opportunity to follow direct fibroblast melanoma cell interactions. This model, however, does not allow for studying the interaction with immune cells. Previously, an adaptive signaling network in melanoma inflammatory niches has been reported to confer melanoma progression. Macrophage‐derived IL‐1β is mandatory for the induction of fibroblast CXCR2 ligands like IL‐8 and Gro‐α, and is also responsible for IL‐6 release. Inhibition of IL‐1 receptor signaling in vivo reduces melanoma growth (Young et al. 2017). Our data can be reconciled with this previous report if one considers that, in the absence or reduced release of IL‐1β, as shown in our experiments, neither IL‐8 nor IL‐6 nor Gro‐α can be released by senescent fibroblasts. However, GCP‐2 released from senescent fibroblasts can—independent of IL‐1β signaling—enhances CREB activation in melanoma cells and thereby enforces melanoma progression. Apart from the here studied GCP‐2 effects on melanoma cells enforcing melanoma progression, GCP‐2 has been reported to attract neutrophils (Wuyts et al. 1997). We observed a slight increase in the number of myeloperoxidase‐positive neutrophils in melanomas from aged patients, in contrast to melanomas from young patients. We currently cannot exclude that neutrophils may play an additional role in melanoma progression, as reported previously in a murine melanoma model after exposure to UV‐irradiation (Bald et al. 2014).

As to the question of how GCP‐2 activates CREB, the following sequence of events is likely: GCP‐2 binds to its receptor, like CXCR1/2 (Wuyts et al. 1997), belonging to the G protein‐coupled receptors. Thereafter, the G protein complex exchanges GDP for GTP. This activates *adenyl cyclase*, which, in consequence, catalyzes the conversion of ATP into cyclic adenosine monophosphate (cAMP), thereby enforcing a profound increase in cAMP concentrations. The binding of 4 cAMP molecules to Protein Kinase A (PKA) induces a conformational change in its regulatory subunits, causing PKA activation ^48,49^, which phosphorylates (activates) CREB. In fact, we found that PKA expression is increased in melanomas from old patients as opposed to young melanoma patients (data not shown).

Our data now allows several options for novel strategies for therapeutic intervention. Targeting GCP‐2 or the CXCR1 and CXCR2 receptors relaying signals of GCP‐2 to activate CREB or CREB itself might be a unique possibility to suppress melanoma progression in old patients. Neutralizing antibodies against GCP‐2 (Verbeke et al. 2011), small molecule inhibitors for the CXCR1/CXCR2 receptors (Campbell et al. 2013), and for CREB (Xiao et al. 2010) have already been shown to reduce tumor progression in breast cancer (Ginestier et al. 2010). Also, small molecule inhibitors for the CXCR1/CXCR2 receptors are already in clinical trials for liver metastasis in colon cancer (Varney et al. 2011), breast cancer (Campbell et al. 2013), and other indications (Citro et al. 2012; Holz et al. 2010; Moss et al. 2013; Nair et al. 2012). It will be of utmost clinical relevance to understand CREB activation and—in consequence—its herein identified metabolic target genes to develop novel precision therapies. In fact, forced overexpression of TCA genes in melanoma cells led to an impressive reduction in melanoma size and to a complete metabolic shift towards TCA (Figure 7C,D). This was particularly effective for citrate synthase and fumarate hydratase—both enzymes may thus qualify as promising therapeutic targets. Furthermore, in the context of our findings, the earlier report on genetically induced apoptosis of p16^Ink4a^ senescent cells with an impressive rejuvenation of old mice (Baker et al. 2011) is of potential interest in the long perspective. Accordingly, a senolytic therapy with ablation of senescent fibroblasts and, in consequence, their secretory phenotype, preventing CREB activation, may effectively complement conventional therapies for melanoma and other tumor entities in elderly patients.

## 4 Experimental Procedures

### 4.1 Generation of Conditioned Medium

Normal diploid human foreskin fibroblasts were cultured at high density (10^5^ cells/cm^2^) to release factors into the culture medium. The cumulative population doubling (CPD) was determined as published (Bayreuther et al. 1992). Young non‐senescent fibroblasts (NON SEN) at CPDs ranging between 5 and 14 and replicative senescent fibroblasts (REP SEN) of the human dermis‐derived strains FF95 and FFRa at CPDs between 60 and 70 were incubated at 37°C, 21% O_2_, and 5% CO_2_ as described earlier (Millis et al. 1977). Supernatants were centrifuged at 1000 rpm for 5 min at 4°C to remove cell debris, passed through a 40 μm cell strainer (352340, BD Bioscience, Europe), and thereafter referred to as “conditioned medium” (CM). We purposely generated CM in the presence of 10% FCS to avoid a starvation stress response during the 10 days of fibroblast culture. This allowed us to model the conditions of human skin with interstitial fluid containing serum proteins.

### 4.2 Melanoma Cell Lines

Melanoma cell lines were procured from A.T.C.C. and grown as per their guidelines. Melanoma cell lines were grown in DMEM with 10% FCS supplemented with (2 mM L‐glutamine and 100 IU of penicillin–streptomycin)/mL of DMEM.

### 4.3 Primary Malignant Melanoma and Healthy Human Skin

Biopsy materials were obtained from the Department of Dermatology and Allergic Diseases, Ulm, Germany, and from the Department of Dermatology, Cologne University, Cologne, Germany, as shown in Table S1.

The records of melanoma patients of 135 subsequent patients with location‐matched primary melanoma were used to establish a correlation between the age of the patients and tumor depth. The collection and analysis of human melanoma biopsies and healthy skin of patients and healthy volunteer individuals at different ages was performed with informed consent and approval by the Institutional Review Board at the University of Ulm and Cologne (155/2005 and 08‐144). Further clinical information on the samples is given in Table S2.

### 4.4 Transwell Migration Assay

Migration of different melanoma cells (Table S3) was assessed by means of the Transwell chamber technique (Corning GmbH, Germany) with slight adaptations to an earlier described protocol (Scharffetter‐Kochanek et al. 1992). The lower compartment of the chamber was loaded with 600 μL of conditioned medium derived from supernatants of senescent and young dermal fibroblasts or recombinant chemokines diluted to concentrations of 1, 10, and 30 nM in serum‐free medium (UltraCULTURE medium, Lonza). The upper compartment was loaded with 10^5^ A375 or other melanoma cells/well in 200 μL serum‐free medium. Following a 6 h incubation period at 37°C, 5% CO_2_, perforated filters were removed, the upper chamber and the polycarbonate membrane were swapped clean with cotton ear buds, and the membrane was further fixed and stained with DiffQuick Stain (Medion Diagnostics AG). The migrated cell count was analyzed from three technical replicates in 7 high‐power fields (20×). The experiment was independently repeated three times. The statistical significance of the migration index in different groups was calculated using ANOVA multiple comparison with *Tukey*'*s* posthoc test. *p* values < 0.0001 were considered significant.

### 4.5 Anchorage Independent Growth Assay

The anchorage‐independent growth assay was performed according to the manufacturer's instructions (CellBio Labs Inc.). Briefly, 10^5^ melanoma cells or melanocytes in DMEM without FCS were mixed with 1.2% top agar and 2× DMEM without FCS in a 1:1:1 ratio. 75 μL thereof was placed in each well of a 96‐well flat‐bottom plate, resulting in 10^4^ cells/well. The plate was incubated for 15 min at 4°C, and thereafter cells within the soft agar were overlaid with 100 μL of serum‐free medium or CM from old or young fibroblasts. The plates were incubated at 37°C, in an atmosphere of 5% CO_2_, for 7 days and were analyzed using a Zeiss inverted microscope. The total number of colonies was counted after dissolving the agar and subsequent incubation with SYBR green. The fluorescence intensity as a measure of colony formation was determined at 485/520 nm.

### 4.6 Animals

SCID mice were used for the xenotransplantation assays (Laboratory of Cell Proliferation & Aging, Institute Biosciences and Applications, NCSR “Demokritos,” Athens, Greece). All animals were bred and maintained in the Animal Facility of the above Institute. All animal studies were conducted according to the institutional guidelines conforming to international standards, and the protocol was approved by the relevant committee of the Veterinary Direction, Greek Ministry of Rural Development and Food (Protocol number 6455 and 3751) as previously published (Papadopoulou and Kletsas 2011).

### 4.7 Construction of Lentiviral Vectors Containing Specific shRNA for Human GCP‐2 and ENA‐78

The pLVX‐ShRNA2 vector (Clontech) with eGFP reporter gene sequence was used to distinguish between single transgenic cells and double transgenic cells. Phosphorylated ShRNA2 oligos with restricted compatible ends matching the vector backbone were generated from Thermoscientific polymers and diluted to a final concentration of 1 pmol/μL. The complementary ShRNA oligos were added in NEB buffer 2. Complementary strands were annealed at 95°C for 5 min with subsequent slow cooling to room temperature in 1× annealing buffer (NEB buffer 2). Thereafter, annealed oligos were ligated to the dephosphorylated digested vector backbone and transformed into STBL4 *E. coli* competent cells (Invitrogen). Transformed clones were assessed by colony PCR and sequencing. Plasmids were transfected with TransIT‐LT1 (MirusBio) in Lenti‐X cells (Clontech), and viral particles were concentrated by spin centrifugation at 25,000 rpm for 2.5 h at 4°C. Fibroblasts and melanoma cells were transduced with polybrene (1 μg/μL) (Millipore) and incubated for 24 h. A375 melanoma cells co‐injected with young or old fibroblasts of different genotypes were assessed in the xenotransplant SCID model for the role of old fibroblasts and GCP‐2 and ENA‐78 on tumor size. Sequences of ShRNA hairpin used for generating transgenic cell lines are summarized in Table S3.

### 4.8 Xenograft Tumorigenesis Model

Male SCID mice 8 weeks of age were injected subcutaneously in the flank with 10^6^ A375 melanoma cells either alone or in combination with replicative senescent (REP SEN, old CPD 68) and non‐senescent (NON SEN, young CPD 14) FF95 fibroblasts in a total volume of 200 μL. The tumor volume was calculated using the following equation: V = 0.5 × L × W^2^, where V is the melanoma volume, L represents the melanoma length, and W is the melanoma width. Five mice of the following genotypes were assessed (*n* = 5 per group). (1) A375MM + REP SEN fibroblasts, (2) A375MM + NON SEN, fibroblasts, (3) A375 MM ^GCP‐2KDN/ENAKDN^ + REP SEN fibroblasts, (4) A375 MM ^GCP‐2KDN/ENAKDN^ + REP SEN fibroblasts^GCP‐2KDN^, (5) A375 MM ^GCP‐2KDN/ENAKDN^ + REP SEN fibroblasts ^ENA78KDN^, (6) A375 MM ^GCP‐2KDN/ENAKDN^ + NON SEN fibroblasts, (7) A375 MM ^GCP‐2KDN/ENAKDN^ + NON SEN fibroblasts^GCP‐2KDN^, (8) A375 MM ^GCP‐2KDN/ENAKDN^ + NON SEN fibroblasts^ENA‐78KDN^. Animal experiments were performed in accordance with the regulations of the Greek government.

### 4.9 Transcript PCR, Antibody Arrays, and ELISA

To identify SASP factors released by senescent fibroblasts which promote tumor progression, young non‐senescent and replicative senescent fibroblasts and the CM thereof were subjected to complementary qPCR‐based transcriptome analyses (RT^2^ Profiler, Cat. No.: 330231, Qiagen) and to antibody arrays for cytokines, chemokines, and their corresponding receptors (RayBiotech, Chemokine Array Cat. No. GSH‐CHE‐1, Cytokine Array Cat. No. QAH‐CAA‐2000). After normalization for cell number and mRNA quantity, mRNA was subjected to cytokine and chemokine transcriptome analysis using a qPCR‐based cytokine, chemokine, and receptor array. In parallel, supernatants of cell number‐normalized CM were analyzed on human chemokine, cytokine antibody arrays containing a panel for 174 cytokines and 38 chemokines. Concentrations of specific GCP‐2, ENA‐78, and RANTES were assessed in the CM with an ELISA (Cat. No. DGC00, GCP‐2, Cat. No. DX000, ENA‐78, and Cat. No. DRN00B, RANTES, R&D Systems) according to the manufacturer's instructions. Only those factors that were found to be consistently upregulated both on the mRNA and protein levels, as assessed by antibody arrays and specific ELISAs, with a positive Pearson's correlation between 2 different primary fibroblast strains, were further analyzed.

### 4.10 Immunofluorescence and Immunochemistry

Immunostaining of cryosections and paraffin sections obtained either from xeno‐ and isotransplant murine models, from skin of healthy individuals, and from melanomas (Department of Dermatology and Allergic Diseases, University of Ulm, Germany) was performed using a previously described protocol (Wang et al. 2006). Frozen cryosections and paraffin sections of melanomas were incubated with antibodies against (p16^INK4a^ #G175‐405; BD Biosciences), (FSP‐1 #TE‐7; Millipore), (Rabbit anti‐MelanA #EP1422Y; Abcam), (Mouse Anti‐Melan A #A103; Dako), and young and senescent fibroblast cells (p53BP1 #PA1‐46147; Thermo Scientific), (Ki67 #SP6; Thermo Scientific), (p16^INK4a^ #G175‐405; BD Biosciences), (GCP‐2 #10L25; GeneTex), (ENA‐78 #EPR4450; GeneTex) and young and aged skin biopsy's were incubated with (CD18 #YFC 118.3; GeneTex), (Vimentin; V9; Millipore), (CD31 #ab28364; Abcam), (GCP‐2 #10L25; GeneTex), (ENA‐78; EPR4450 #GeneTex). Young and aged murine skin was incubated with antibodies against (Lix #32B3, GeneTex). Tumor biopsy from SCID mice were incubated with antibodies against (pCREB^S133^ #87G3; Cell Signaling Technologies), (cleaved caspase‐3 #D3E9; Cell Signaling Technologies), (pCS1 #SC33397; Santacruz), (pPDH‐E1a #AP1062; Millipore), (PDHK1 #3820; LDHA/C #3558; ACO2 #6571; Cell signaling Technologies) and (Ki67; SP6; Thermo Scientific). Isotype IgG served as a negative control, and Alexa Fluor 488 and Alexa Fluor 555 (Molecular Probes) served as secondary antibodies. Immunohistochemistry of melanoma sections were incubated with secondary goat ([Anti‐Rabbit‐Biotin; BA‐1000] and [Anti‐Mouse‐Biotin; BA‐2000]; Vector Laboratories) antibodies and further developed with ([ABC‐AP; AK‐5003] and [ABC‐Elite; PK‐6103]; Vector Laboratories) and substrates ([Vector Blue Alkaline Peroxidase; SK‐5300], [ImmPACT DAB HRP; SK‐4105] and [ImmPACT NovaRed HRP; SK‐4805]; Vector Laboratories). Photomicrographs were taken using a Zeiss Axiophot microscope and AxioVison 4.8 software (Zeiss).

### 4.11 Western Blotting

Cells were stimulated with chemokines for 1 h, harvested, and lysed with 1xRIPA containing protease and phosphatase inhibitors (Roche) for a 20 min incubation period on ice. Lysates were centrifuged at 13,000 rpm for 30 min. Protein quantification was performed by BCA assay (Pierce). Lysates were loaded with 4× LDS sample buffer (Nupage) and subjected to electrophoresis in a 4%–12% Bis‐Tris polyacrylamide gel (Novex, LifeTechnologies). Antibodies such as pCREB S133 #Clone 10E9 were purchased from Millipore and (HexI #2024; Hex II #2867; pPFKFB2 #13064; G3PDH #2118; PDH #3205; IDH1 #8137, IDH2 #12652; OGDH #13407; GDH1/2 #12793; SDH #11998; FH #4567; Cell Signalling Technologies) were diluted 1:1000 in 3% SKMT and incubated overnight at 4°C. Visualization was performed by enhanced chemiluminescence (ECL kit, Amersham Biosciences).

### 4.12 Phosphokinase Analysis of Melanoma Cells

Melanoma cells were incubated with conditioned media (CM) derived from 10^5^ non‐senescent or replicative senescent fibroblasts for a period of 5–6 h. The media were washed with cold PBS, the 375 melanoma cells were lysed, and the protein amount was quantified. 200 μg of cell lysate was dissolved in 2 mL of array buffer 1 and incubated on the phosphokinase array (ARY003B, R&D Systems Inc.) overnight at 4°C with gentle rocking as per the manufacturer's protocol. Membranes were washed three times for 10 min in 1 × washing buffer and further hybridized with diluted streptavidin‐HRP conjugated antibody for a period of 30 min at room temperature. Thereafter, the membranes were washed and developed with chemiluminescence reagent in the Vilber Lourmat chemiluminescence imaging system using the Fusion II and Bio‐1D Image analysis and quantification software.

### 4.13 Phospho Protein Enrichment and 2D Electrophoresis

The phospho‐proteins were enriched from the cell lysates of CREB^WT^, CREB^TG^, and CREB^S133A^, and further subject to 2D electrophoresis using the Phosphoprotein Enrichment Kit (#90003, Pierce) was employed according to the manufacturer's instructions. Briefly, A375 melanoma cells were scraped at 4°C and lysed using lysis buffer containing 0.25% CHAPS, 1X phosphatase and protease inhibitor cocktail (#78420, Pierce), and incubated for 45 min. The protein concentration was determined by Bradford assay, and up to 4 mg of protein were loaded on a washed/equilibrated phosphate‐enrichment column and incubated at 4°C on an orbital shaker for an hour. The column was washed three times, 1 min each, and finally eluted in 1 mL of elution buffer with 0.25% CHAPS. The eluted protein was further desalted with a Zeba Desalting column (#89877, Thermoscientific). A final concentration of 60 μg of desalted purified protein was used with a broad range carrier Ampholyte (SERVALYT) between (pH 2–11) on water at a 1:40 ratio. The gels were scanned, and the spots were analyzed, quantified, and digitally colored using Delta2D GmbH software.

### 4.14 Maldi‐TOF/MS of Phospho‐Proteins

#### 4.14.1 NanoHPLC‐ESI‐MS/MS

Protein identification was performed by Proteome Factory (Proteome Factory AG, Berlin, Germany). The LCMS system consisted of an Agilent 1100 nanoHPLC system (Agilent, Waldbronn, Germany), PicoTip electrospray emitter (New Objective, Woburn, MA), and an Orbitrap XL or LTQ‐FT Ultra mass spectrometer (ThermoFisher Scientific, Bremen, Germany). Protein spots were in‐gel digested by trypsin (Promega, Mannheim, Germany) and analyzed by nanoHPLC‐ESI‐MS/MS. Peptides were first trapped and desalted on the enrichment column (Zorbax 300SB‐C18, 0.3 × 5 mm, Agilent) for 5 min (solvent: 2.5% acetonitrile/0.5% formic acid), then separated on a Zorbax 300SB‐C18, 75 μm × 150 mm column (Agilent) using a linear gradient from 10% to 32% B (solvent A: 5% acetonitrile in water, solvent B: acetonitrile, both with 0.1% formic acid). Ions of interest were data‐dependently subjected to MS/MS according to the expected charge state distribution of peptide ions.

### 4.15 Peptide Spectrum Mapping

Proteins were identified by database search against the human subset of the SwissProt_2016_database using MS/MS ESI‐TRAP ion search of the Mascot Matrix search (Matrix Science, London, England). The Ms/Ms tolerance was set to 5 ppm and 0.6 Da. Post Translation Modification (PTM) modifications, both (Fixed) Carbamidomethyl (C) at the Cys residue and (Variable) Deamidated (NQ), Phospho (ST& Y) were analyzed using the Peaks PTM function. Only peptides matching with a score of 20 or above and an emPAI score above 2.0 were accepted for further analysis. The best peptide spectrum match (PSM) was estimated using the Target‐Decoy method with 1% FDR, where FDR = #decoy/#Target. De Novo sequences were verified using the Peak DataBase (DB) Spider homologous search function from Peaks 7.5 software.

### 4.16 Metabolomics

#### 4.16.1 Melanoma Cells

The human A375 melanoma cell lines and human melanocytes were grown to 80% confluence, rinsed with PBS and with serum‐free medium, and incubated in serum‐free medium for 2 h. Thereafter, cells were harvested, washed with PBS, and counted. Sample preparation was done using a methanol/chloroform extraction protocol with a 20 μL sample volume of frozen cell pellets according to the manufacturer's instructions (BIOCRATES Life Sciences AG, Innsbruck, Austria). The non‐diluted extracts were measured with two acquisition methods, and the diluted extracts were measured with a third acquisition method. Targeted metabolomics analysis was performed using the validated in‐house method LIPIDS/Intermediate energy metabolites and amino acids analyzed on an ABSCiex triple‐quadrupole mass spectrometer operating in positive and negative MRM mode (BIOCRATES Life Sciences AG, Innsbruck, Austria). Forty‐three calibrators in 7 levels and 5 internal standards (3 of them were deuterated) were used to measure a panel of glycerophospholipids and sphingolipids. Data analysis was performed using the MetIDQ software (BIOCRATES Life Sciences AG, Innsbruck, Austria). An isotope‐correction tool was included to correct and recalculate the measured signals, to avoid any influence of neighboring MRMs, and to ensure the quality of the measurement. Melanoma patient samples were processed according to the company's protocol (ULTRAFREE‐MC‐PLHCC, Human Metabolome Technologies, Yamagata, Japan). Briefly, the samples were mixed with 50% acetonitrile in water (v/v) containing internal standards (20 μM) and homogenized by a homogenizer (3500 rpm, 60 s × 20 times); then, the same amount of 50% acetonitrile in water (v/v) was added. The supernatant (400 μL) was then filtered through a 5‐kDa cut‐off filter to remove macromolecules. The filtrate was centrifugally concentrated and resuspended in 50 μL of ultrapure water immediately before the measurement. Cationic compounds were measured in the cation mode of metabolome analysis using the CE‐TOFMS Agilent CE‐TOFMS system (Agilent Technologies), and the anionic compounds were measured using the Agilent 6460 TripleQuad LC/MS Machine. All the metabolite concentrations were calculated by normalizing the peak area of each metabolite with respect to the area of the internal standard and by using standard curves, which were obtained by three‐point calibrations, and the data with hierarchical clustering and enrichment analysis were carried out using GSEA and R Package tools. Analysis of glutagenic and ketogenic amino acids from the metabolite was plotted using GraphPad Prism software.

#### 4.16.2 Human Melanoma

The human melanoma patient samples were mixed with 50% acetonitrile in water (v/v) containing internal standards (20 μM) and homogenized by a homogenizer (3500 rpm, 60 s × 20 times). Thereafter, the same amount of 50% acetonitrile in water (v/v) was added. The supernatant thereof (400 μL) was then filtered through a 5‐kDa cut‐off filter (ULTRAFREE‐MC‐PLHCC, Human Metabolome Technologies, Yamagata, Japan) to remove macromolecules. The filtrate was centrifugally concentrated and resuspended in 50 μL of ultrapure water immediately before the measurement. Cationic compounds were measured in the cation mode of metabolome analysis using the CE‐TOFMS Agilent CE‐TOFMS system (Agilent Technologies), and the anionic compounds were measured using the Agilent 6460 TripleQuad LC/MS Machine.

Peaks detected in CE‐TOFMS analysis were extracted using automatic integration software (MasterHands ver.2.17.1.11 developed at Keio University) (4), and those in CE‐QqQMS analysis were extracted using automatic integration software (MassHunter Quantitative Analysis B.06.00 Agilent Technologies, Santa Clara, CA, USA) to obtain peak information including m/z, migration time (MT), and peak area. The peak area was then converted to relative peak area by the following equation 1. The peaks were annotated based on the migration times in CE and m/z values determined by TOFMS.

In addition, absolute quantification was performed in 116 metabolites, including glycolytic and TCA cycle intermediates, amino acids, and nucleic acids. All metabolite concentrations were calculated by normalizing the peak area of each metabolite with respect to the area of the internal standard and by using standard curves, which were obtained by three‐point calibrations.

### 4.17 ECAR and OCR Measurements

Oxygen consumption rate (OCR) and extracellular acidification rate (ECAR) were measured by Seahorse Bioanalyzer XF^e^96.2 × 10^4^ cells were seeded per well in a 96‐well Seahorse XF plate with overnight incubation at 21% O_2_ and 5% CO_2_. Cells were incubated for 60 min in an XF 96 incubator in 50 μL XF Assay medium containing 0 mM Glucose (Seahorse Bioscience, # 101022‐100) before ECAR/OCR estimation. Later, the assay medium was replaced with 180 μL of fresh assay medium containing 5 mM physiological glucose. The assay was carried out with Port A, B, C, and D containing 2 μM Oligomycin, blank, 0.5 μM 2‐Deoxy‐Glucose, and 0.5 μM (Rotenone + Antimycin). In the case of the CREB inhibitor (CREBi, 100 nM CREB), it was delivered through port B. The analysis was carried out with *WAVE* software (Seahorse Biosciences). The graphical representation of the assay was carried out using GraphPad Prism and Wave Software. The data represent the mean value of 4 technical replicates carried out with three independent melanoma cell lines and compared using ANOVA. Significance was defined as *p* < 0.05.

### 4.18 Statistics

Data were analyzed with GraphPad Prism software (GraphPad Software Inc., San Diego, CA). One‐way analysis of variance (ANOVA) was used with *Tukey*'*s* post hoc analysis for comparison between multiple groups. The Student's *t*‐test was used for comparison between two groups. Significance was defined as *p* < 0.05. For rank‐based correlation between the tumor thickness in cohorts of young and old melanoma patients, the Mann–Whitney *U* test was performed. For correlation between the ratio of Old/REP SEN versus Young/NON SEN fibroblast expression values of chemokines and cytokines from two independent primary fibroblast lines, Pearson's correlation was carried out, with an *R* ^2^ value of 0.8 was considered highly correlated and plotted using R.

## Author Contributions

Abhijit Basu performed most of the experiments, developed the conceptual design of experiments, and prepared the manuscript. Vida Farsam assisted with the in vitro transwell migration. Dimitris Kletsas mainly contributed to the experimental design of murine experiments and performed these experiments with ethical approval from his institution. Daniel Brandt and Martin Jastroch supported the studies on metabolomics. Jennifer I. Engelmeyer performed the screening with antibody arrays. Nicolai Treiber, Lars Alexander Schneider, Margit Huber, Hartmut Geiger, Karmveer Singh, and Pallab Maity provided essential suggestions for the project and critically reviewed the manuscript. Cornelia Mauch provided us with clinical knowledge, records, and melanoma samples from the Cologne biobank. Karin Scharffetter‐Kochanek designed the scientific concept, translated clinically relevant questions into experimental approaches, and wrote the manuscript.

## Conflicts of Interest

The authors declare no conflicts of interest.

## Supporting information


**Figure S1:** acel70239‐sup‐0001‐FigureS1.pptx.

**Table S1:** acel70239‐sup‐0002‐TableS1.xlsx.

**Table S2:** acel70239‐sup‐0003‐TableS2.xlsx.

**Table S3:** acel70239‐sup‐0004‐TableS3.xlsx.

**Table S4:** acel70239‐sup‐0005‐TableS4.xlsx.

**Table S5:** acel70239‐sup‐0006‐TableS5.xlsx.

## Data Availability

The data that support the findings of this study are available on request from the corresponding author.
