# Supplementary material for: Senescent Fibroblasts Drive Melanoma Progression Through GCP‐2 Induced CREB Phosphorylation Enhancing Glycolysis
Source: Aging Cell. 2025 Nov 19;24(12):e70239. doi: 10.1111/acel.70239 (PMC12686573; doi:10.1111/acel.70239)
Supplement: Supplementary file 1 — Figure S1: acel70239‐sup‐0001‐FigureS1.pptx. [file ACEL-24-e70239-s002.pptx]

## Slide 1
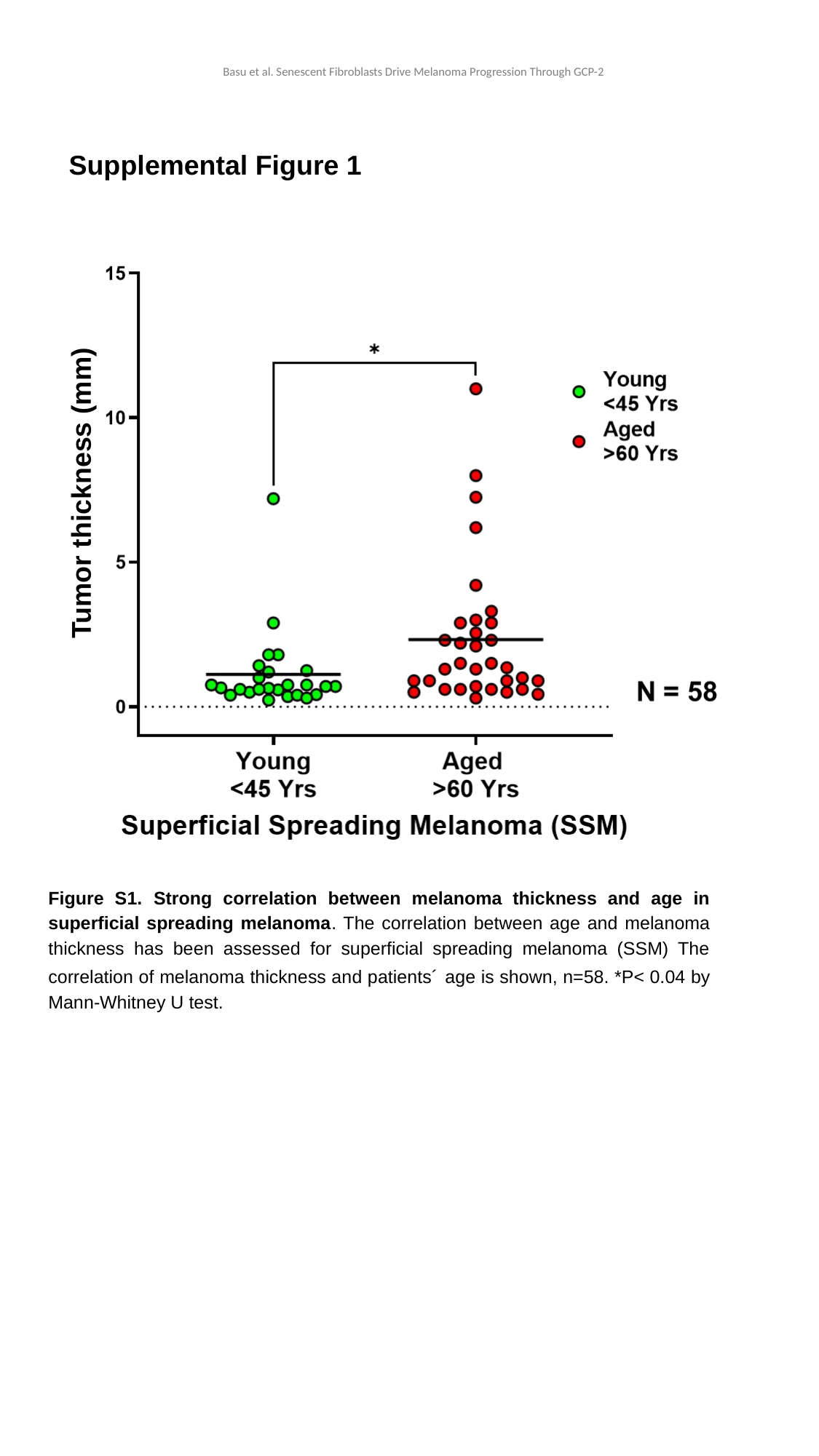

Basu et al. Senescent Fibroblasts Drive Melanoma Progression Through GCP-2
Supplemental Figure 1
Tumor thickness (mm)
Figure S1. Strong correlation between melanoma thickness and age in superficial spreading melanoma. The correlation between age and melanoma thickness has been assessed for superficial spreading melanoma (SSM) The correlation of melanoma thickness and patients´ age is shown, n=58. *P< 0.04 by Mann-Whitney U test.

## Slide 2
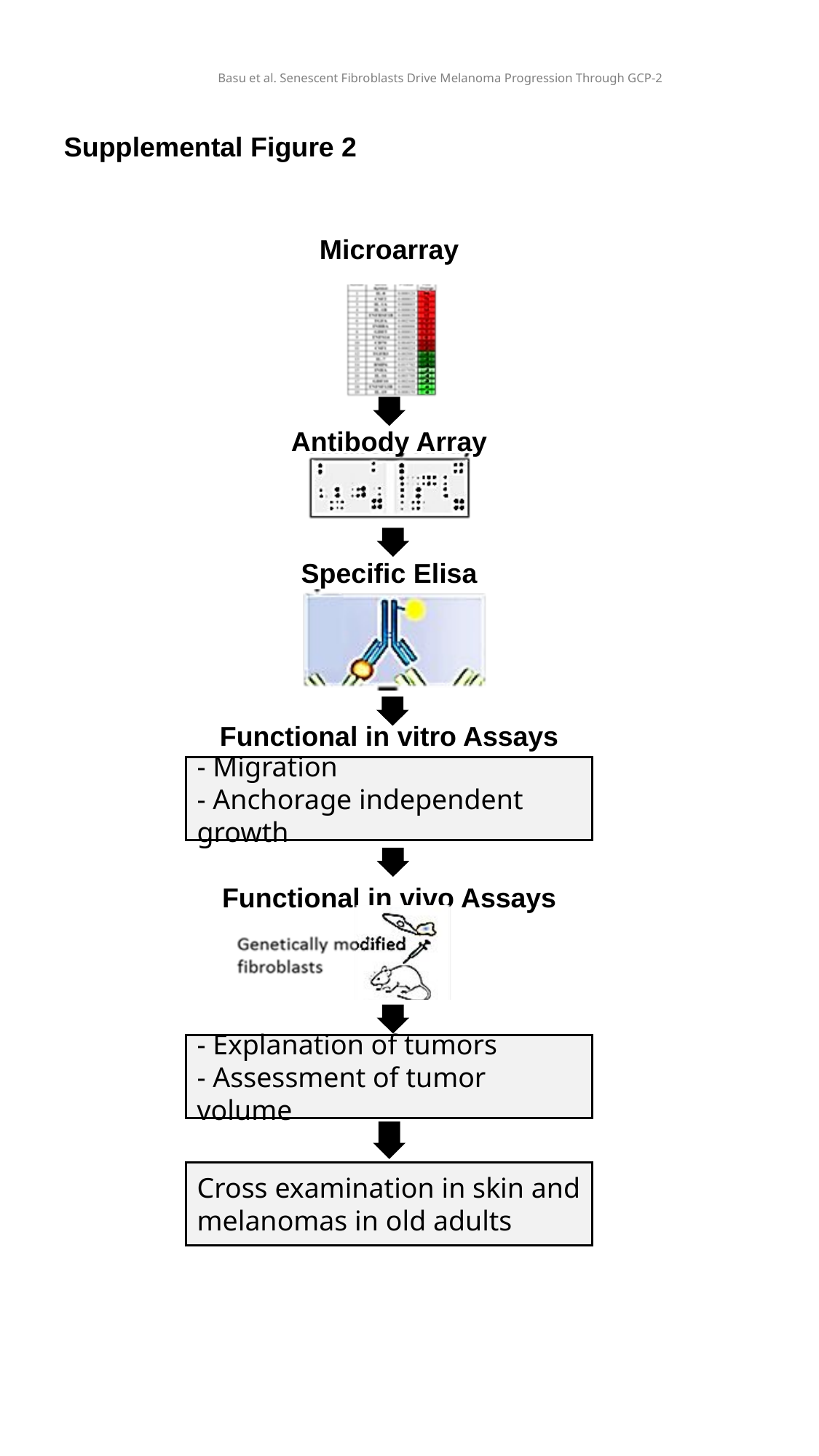

Basu et al. Senescent Fibroblasts Drive Melanoma Progression Through GCP-2
Supplemental Figure 2
Microarray
Antibody Array
Specific Elisa
Functional in vitro Assays
- Migration
- Anchorage independent growth
Functional in vivo Assays
- Explanation of tumors
- Assessment of tumor volume
Cross examination in skin and
melanomas in old adults

## Slide 3
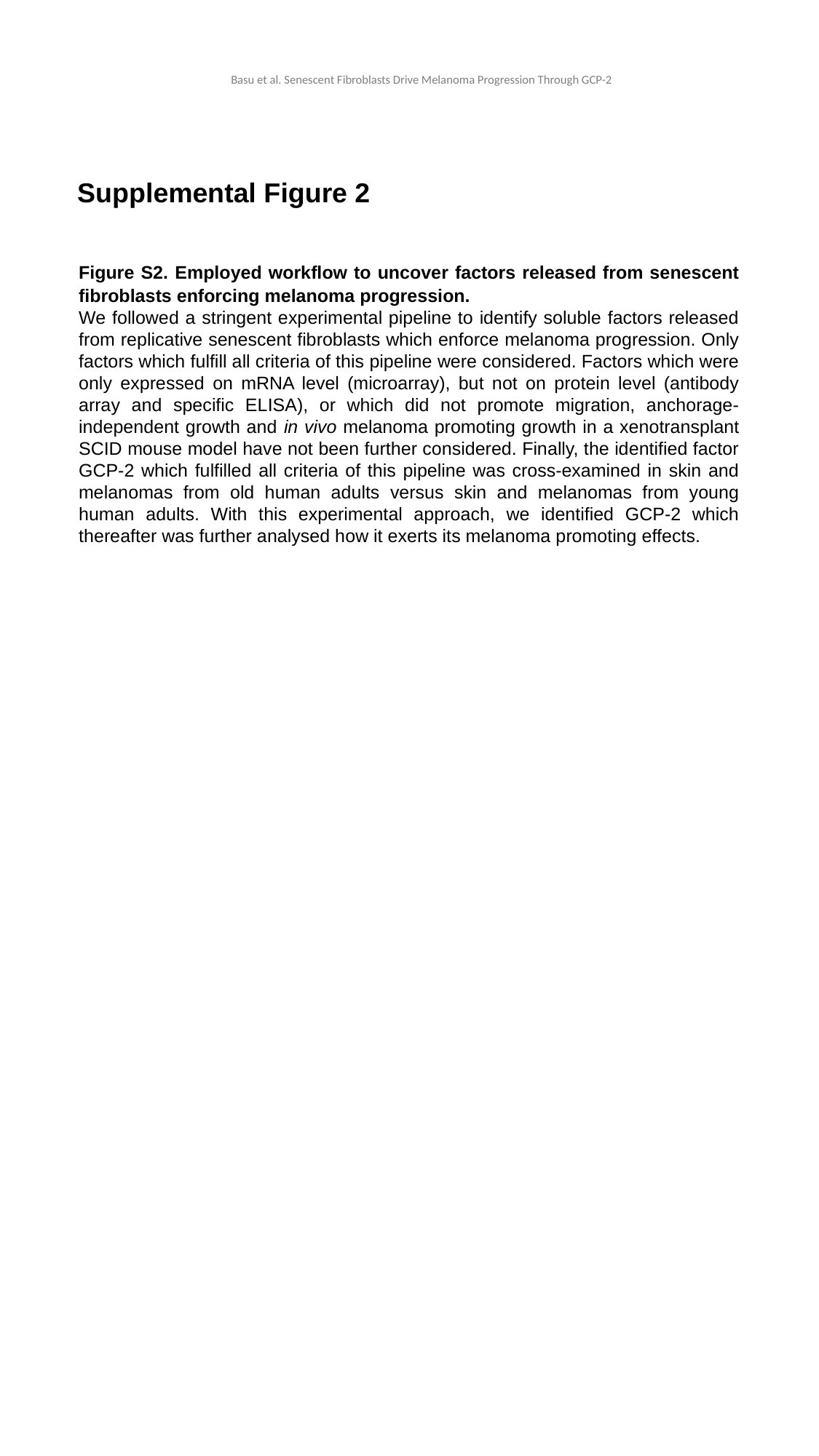

Basu et al. Senescent Fibroblasts Drive Melanoma Progression Through GCP-2
Supplemental Figure 2
Figure S2. Employed workflow to uncover factors released from senescent fibroblasts enforcing melanoma progression.
We followed a stringent experimental pipeline to identify soluble factors released from replicative senescent fibroblasts which enforce melanoma progression. Only factors which fulfill all criteria of this pipeline were considered. Factors which were only expressed on mRNA level (microarray), but not on protein level (antibody array and specific ELISA), or which did not promote migration, anchorage-independent growth and in vivo melanoma promoting growth in a xenotransplant SCID mouse model have not been further considered. Finally, the identified factor GCP-2 which fulfilled all criteria of this pipeline was cross-examined in skin and melanomas from old human adults versus skin and melanomas from young human adults. With this experimental approach, we identified GCP-2 which thereafter was further analysed how it exerts its melanoma promoting effects.

## Slide 4
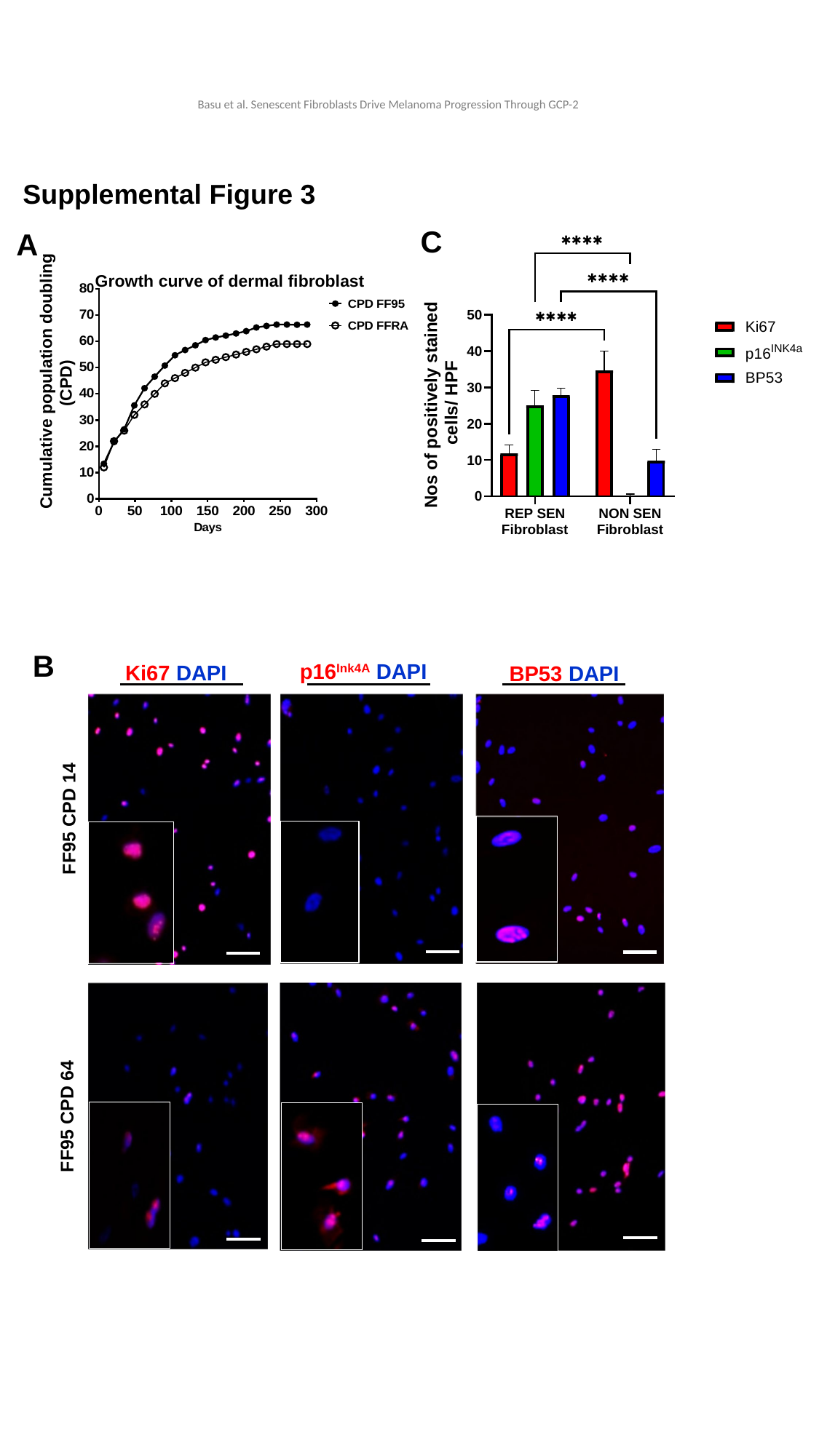

Basu et al. Senescent Fibroblasts Drive Melanoma Progression Through GCP-2
Supplemental Figure 3
C
A
 Growth curve of dermal fibroblast
Cumulative population doubling (CPD)
B
p16Ink4A DAPI
Ki67 DAPI
BP53 DAPI
FF95 CPD 14
FF95 CPD 64

## Slide 5
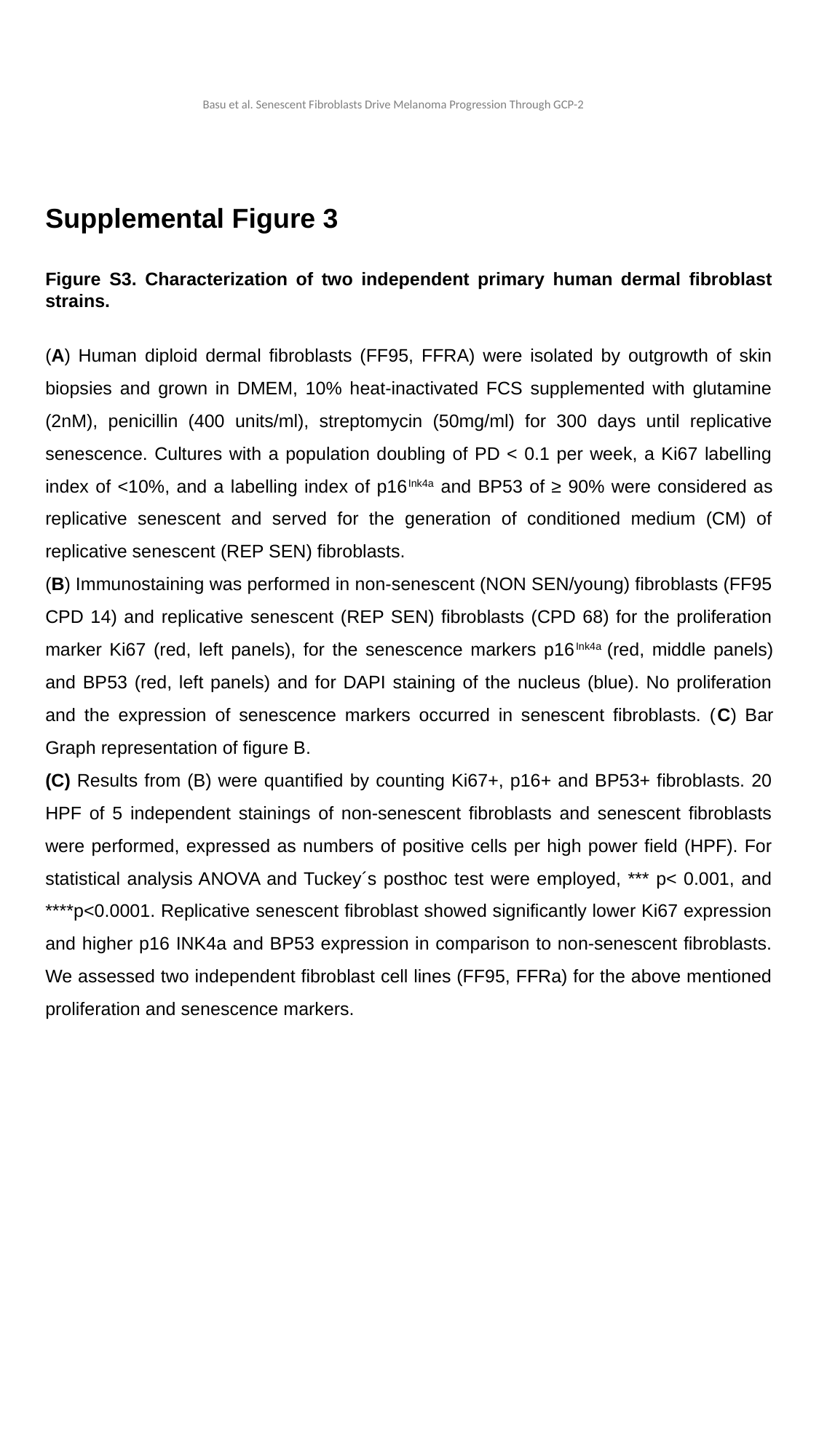

Basu et al. Senescent Fibroblasts Drive Melanoma Progression Through GCP-2
Supplemental Figure 3
Figure S3. Characterization of two independent primary human dermal fibroblast strains.
(A) Human diploid dermal fibroblasts (FF95, FFRA) were isolated by outgrowth of skin biopsies and grown in DMEM, 10% heat-inactivated FCS supplemented with glutamine (2nM), penicillin (400 units/ml), streptomycin (50mg/ml) for 300 days until replicative senescence. Cultures with a population doubling of PD < 0.1 per week, a Ki67 labelling index of <10%, and a labelling index of p16Ink4a and BP53 of ≥ 90% were considered as replicative senescent and served for the generation of conditioned medium (CM) of replicative senescent (REP SEN) fibroblasts.
(B) Immunostaining was performed in non-senescent (NON SEN/young) fibroblasts (FF95 CPD 14) and replicative senescent (REP SEN) fibroblasts (CPD 68) for the proliferation marker Ki67 (red, left panels), for the senescence markers p16Ink4a (red, middle panels) and BP53 (red, left panels) and for DAPI staining of the nucleus (blue). No proliferation and the expression of senescence markers occurred in senescent fibroblasts. (C) Bar Graph representation of figure B.
(C) Results from (B) were quantified by counting Ki67+, p16+ and BP53+ fibroblasts. 20 HPF of 5 independent stainings of non-senescent fibroblasts and senescent fibroblasts were performed, expressed as numbers of positive cells per high power field (HPF). For statistical analysis ANOVA and Tuckey´s posthoc test were employed, *** p< 0.001, and ****p<0.0001. Replicative senescent fibroblast showed significantly lower Ki67 expression and higher p16 INK4a and BP53 expression in comparison to non-senescent fibroblasts. We assessed two independent fibroblast cell lines (FF95, FFRa) for the above mentioned proliferation and senescence markers.

## Slide 6
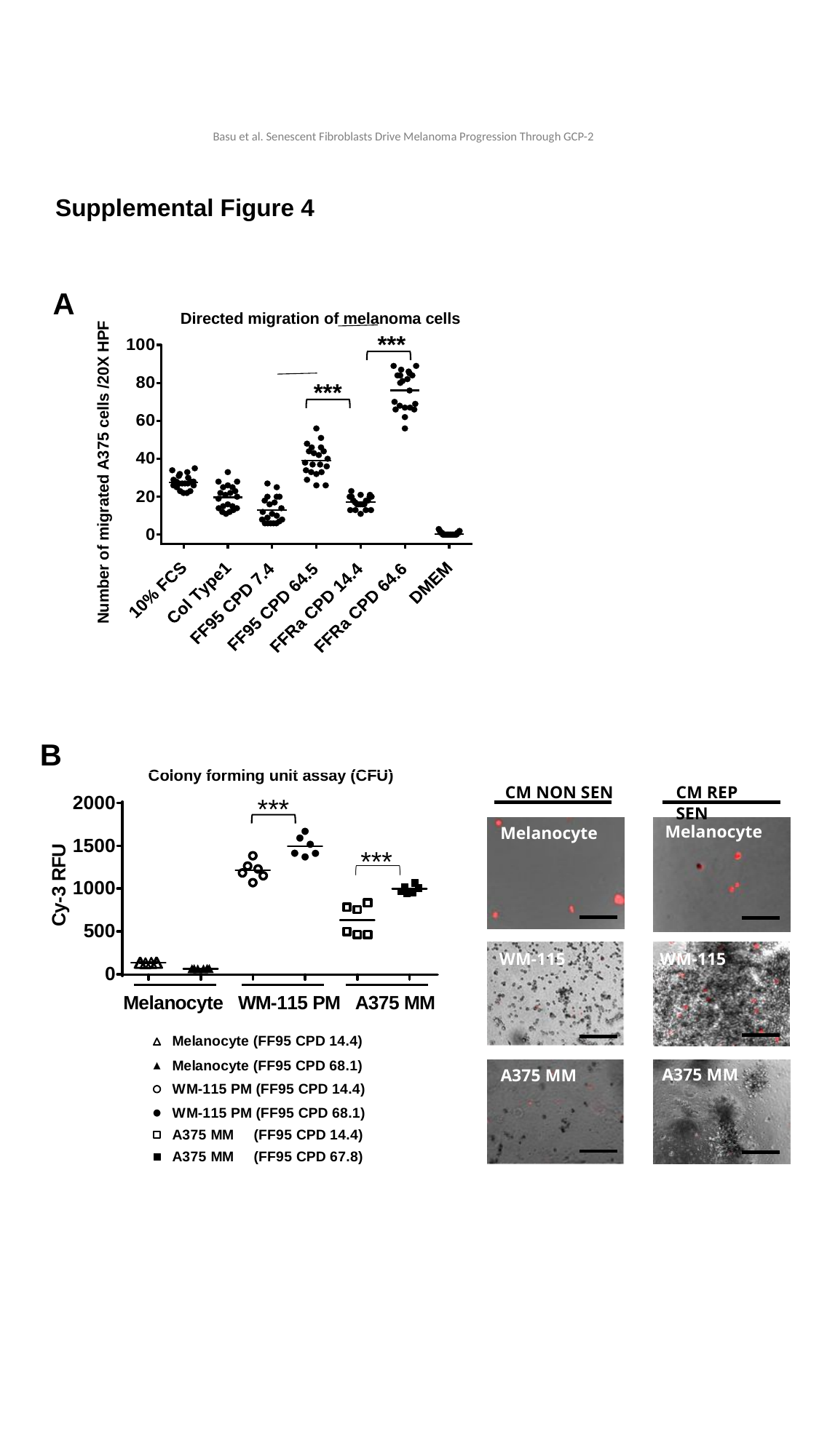

Basu et al. Senescent Fibroblasts Drive Melanoma Progression Through GCP-2
Supplemental Figure 4
A
 Directed migration of melanoma cells
***
***
Number of migrated A375 cells /20X HPF
B
 Colony forming unit assay (CFU)
CM REP SEN
CM NON SEN
***
Melanocyte
Melanocyte
 ***
WM-115
WM-115
A375 MM
A375 MM

## Slide 7
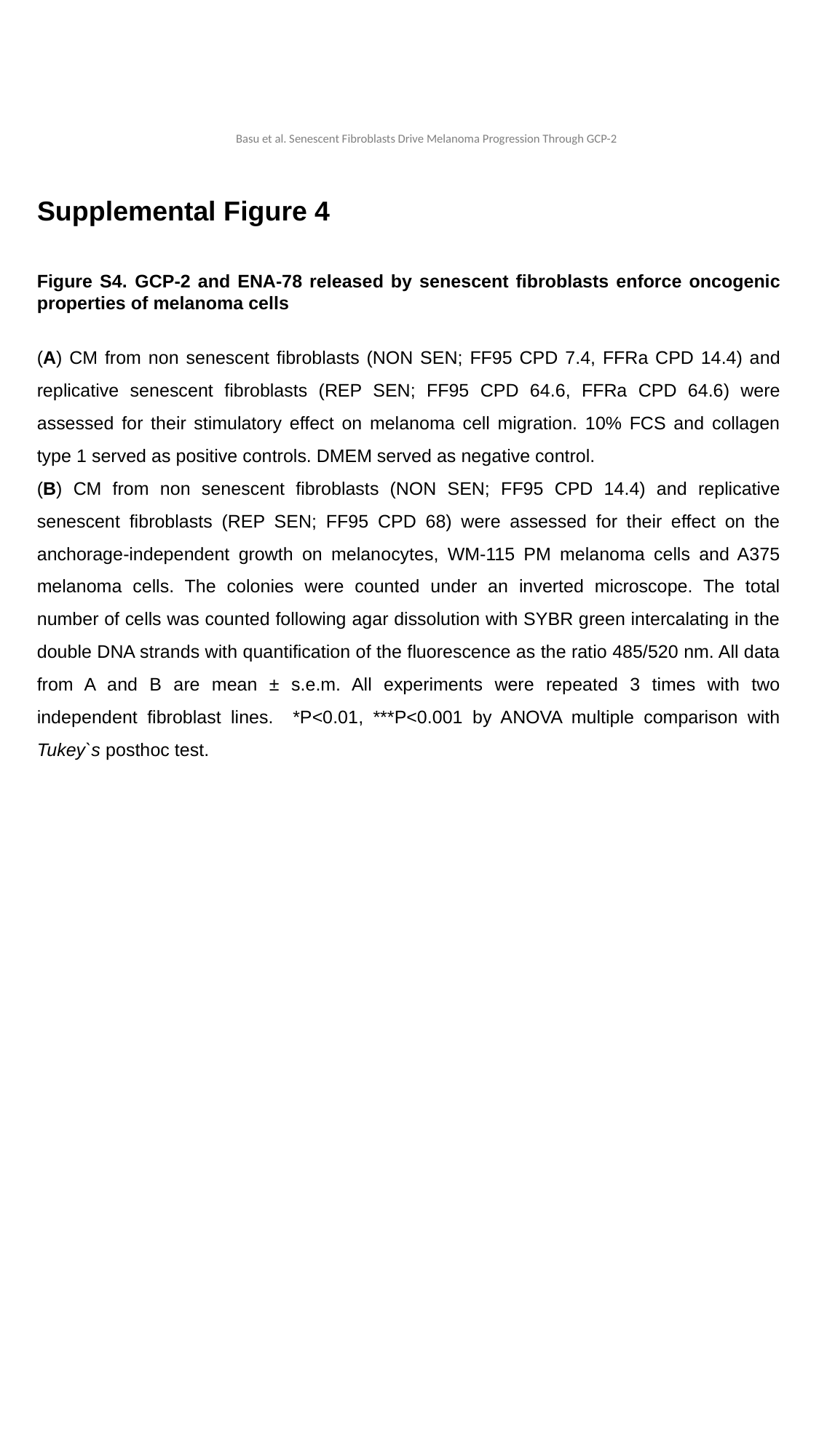

Basu et al. Senescent Fibroblasts Drive Melanoma Progression Through GCP-2
Supplemental Figure 4
Figure S4. GCP-2 and ENA-78 released by senescent fibroblasts enforce oncogenic properties of melanoma cells
(A) CM from non senescent fibroblasts (NON SEN; FF95 CPD 7.4, FFRa CPD 14.4) and replicative senescent fibroblasts (REP SEN; FF95 CPD 64.6, FFRa CPD 64.6) were assessed for their stimulatory effect on melanoma cell migration. 10% FCS and collagen type 1 served as positive controls. DMEM served as negative control.
(B) CM from non senescent fibroblasts (NON SEN; FF95 CPD 14.4) and replicative senescent fibroblasts (REP SEN; FF95 CPD 68) were assessed for their effect on the anchorage-independent growth on melanocytes, WM-115 PM melanoma cells and A375 melanoma cells. The colonies were counted under an inverted microscope. The total number of cells was counted following agar dissolution with SYBR green intercalating in the double DNA strands with quantification of the fluorescence as the ratio 485/520 nm. All data from A and B are mean ± s.e.m. All experiments were repeated 3 times with two independent fibroblast lines. *P<0.01, ***P<0.001 by ANOVA multiple comparison with Tukey`s posthoc test.

## Slide 8
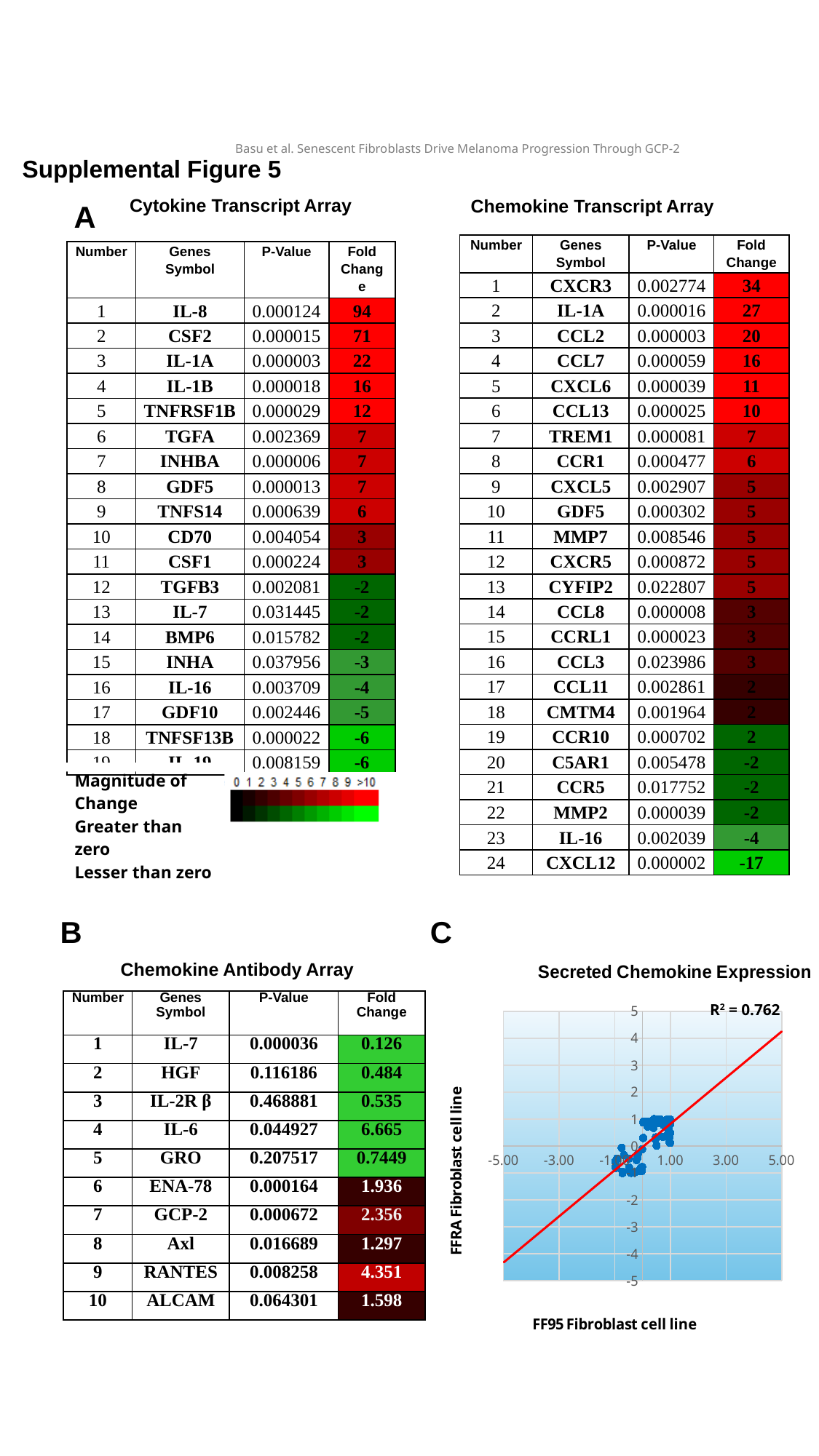

Basu et al. Senescent Fibroblasts Drive Melanoma Progression Through GCP-2
Supplemental Figure 5
Cytokine Transcript Array
Chemokine Transcript Array
A
| Number | Genes Symbol | P-Value | Fold Change |
| --- | --- | --- | --- |
| 1 | CXCR3 | 0.002774 | 34 |
| 2 | IL-1A | 0.000016 | 27 |
| 3 | CCL2 | 0.000003 | 20 |
| 4 | CCL7 | 0.000059 | 16 |
| 5 | CXCL6 | 0.000039 | 11 |
| 6 | CCL13 | 0.000025 | 10 |
| 7 | TREM1 | 0.000081 | 7 |
| 8 | CCR1 | 0.000477 | 6 |
| 9 | CXCL5 | 0.002907 | 5 |
| 10 | GDF5 | 0.000302 | 5 |
| 11 | MMP7 | 0.008546 | 5 |
| 12 | CXCR5 | 0.000872 | 5 |
| 13 | CYFIP2 | 0.022807 | 5 |
| 14 | CCL8 | 0.000008 | 3 |
| 15 | CCRL1 | 0.000023 | 3 |
| 16 | CCL3 | 0.023986 | 3 |
| 17 | CCL11 | 0.002861 | 2 |
| 18 | CMTM4 | 0.001964 | 2 |
| 19 | CCR10 | 0.000702 | 2 |
| 20 | C5AR1 | 0.005478 | -2 |
| 21 | CCR5 | 0.017752 | -2 |
| 22 | MMP2 | 0.000039 | -2 |
| 23 | IL-16 | 0.002039 | -4 |
| 24 | CXCL12 | 0.000002 | -17 |
| Number | Genes Symbol | P-Value | Fold Change |
| --- | --- | --- | --- |
| 1 | IL-8 | 0.000124 | 94 |
| 2 | CSF2 | 0.000015 | 71 |
| 3 | IL-1A | 0.000003 | 22 |
| 4 | IL-1B | 0.000018 | 16 |
| 5 | TNFRSF1B | 0.000029 | 12 |
| 6 | TGFA | 0.002369 | 7 |
| 7 | INHBA | 0.000006 | 7 |
| 8 | GDF5 | 0.000013 | 7 |
| 9 | TNFS14 | 0.000639 | 6 |
| 10 | CD70 | 0.004054 | 3 |
| 11 | CSF1 | 0.000224 | 3 |
| 12 | TGFB3 | 0.002081 | -2 |
| 13 | IL-7 | 0.031445 | -2 |
| 14 | BMP6 | 0.015782 | -2 |
| 15 | INHA | 0.037956 | -3 |
| 16 | IL-16 | 0.003709 | -4 |
| 17 | GDF10 | 0.002446 | -5 |
| 18 | TNFSF13B | 0.000022 | -6 |
| 19 | IL-19 | 0.008159 | -6 |
Magnitude of Change
Greater than zero
Lesser than zero
B
C
Chemokine Antibody Array
### Chart: Secreted Chemokine Expression
| Category | |
|---|---|| Number | Genes Symbol | P-Value | Fold Change |
| --- | --- | --- | --- |
| 1 | IL-7 | 0.000036 | 0.126 |
| 2 | HGF | 0.116186 | 0.484 |
| 3 | IL-2R β | 0.468881 | 0.535 |
| 4 | IL-6 | 0.044927 | 6.665 |
| 5 | GRO | 0.207517 | 0.7449 |
| 6 | ENA-78 | 0.000164 | 1.936 |
| 7 | GCP-2 | 0.000672 | 2.356 |
| 8 | Axl | 0.016689 | 1.297 |
| 9 | RANTES | 0.008258 | 4.351 |
| 10 | ALCAM | 0.064301 | 1.598 |
R2 = 0.762

## Slide 9
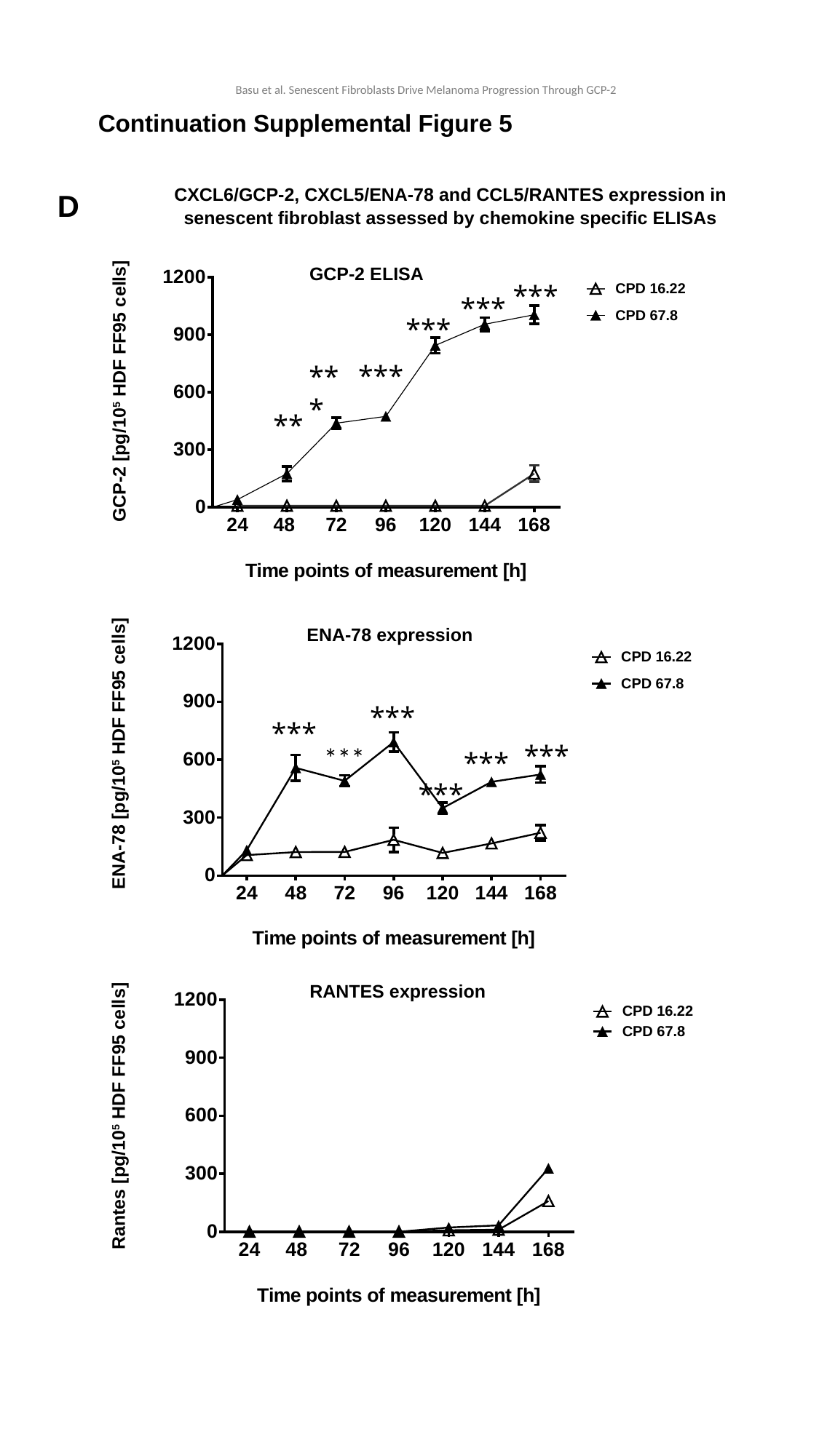

Basu et al. Senescent Fibroblasts Drive Melanoma Progression Through GCP-2
Continuation Supplemental Figure 5
CXCL6/GCP-2, CXCL5/ENA-78 and CCL5/RANTES expression in senescent fibroblast assessed by chemokine specific ELISAs
D
GCP-2 ELISA
***
***
***
***
***
GCP-2 [pg/105 HDF FF95 cells]
**
ENA-78 expression
***
***
***
ENA-78 [pg/105 HDF FF95 cells]
***
***
***
RANTES expression
Rantes [pg/105 HDF FF95 cells]

## Slide 10
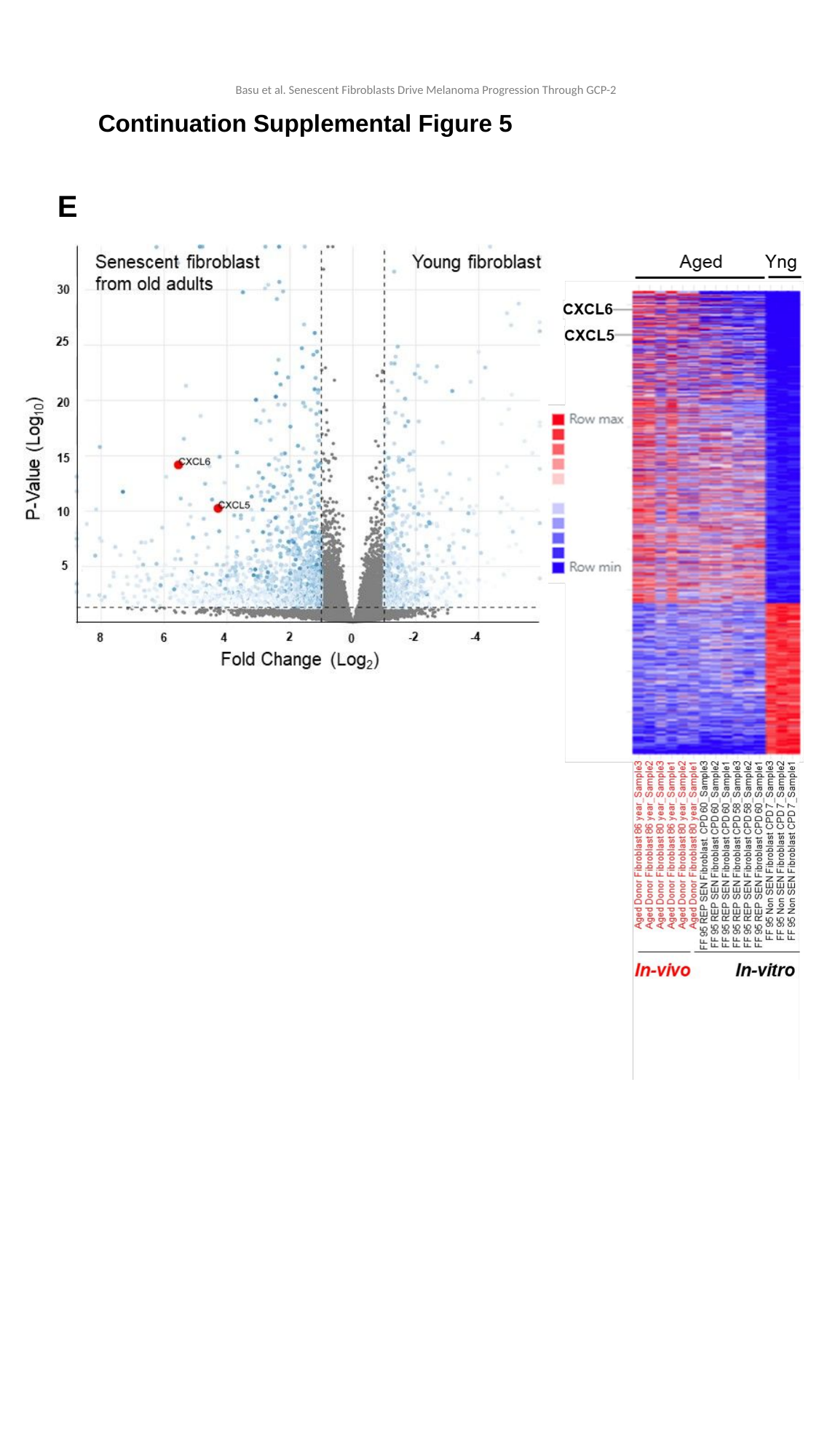

Basu et al. Senescent Fibroblasts Drive Melanoma Progression Through GCP-2
Continuation Supplemental Figure 5
E

## Slide 11
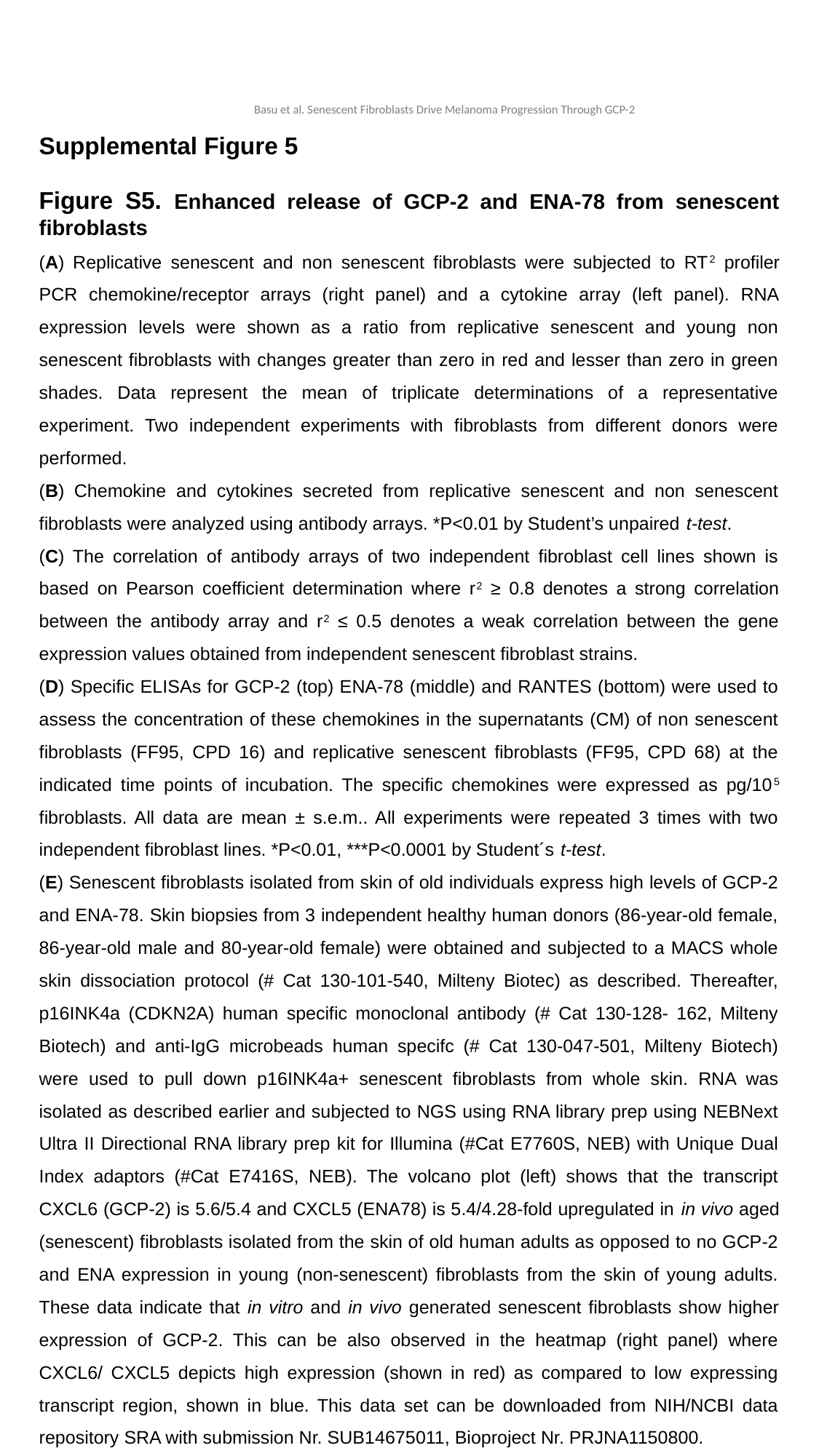

Basu et al. Senescent Fibroblasts Drive Melanoma Progression Through GCP-2
Supplemental Figure 5
Figure S5. Enhanced release of GCP-2 and ENA-78 from senescent fibroblasts
(A) Replicative senescent and non senescent fibroblasts were subjected to RT2 profiler PCR chemokine/receptor arrays (right panel) and a cytokine array (left panel). RNA expression levels were shown as a ratio from replicative senescent and young non senescent fibroblasts with changes greater than zero in red and lesser than zero in green shades. Data represent the mean of triplicate determinations of a representative experiment. Two independent experiments with fibroblasts from different donors were performed.
(B) Chemokine and cytokines secreted from replicative senescent and non senescent fibroblasts were analyzed using antibody arrays. *P<0.01 by Student’s unpaired t-test.
(C) The correlation of antibody arrays of two independent fibroblast cell lines shown is based on Pearson coefficient determination where r2 ≥ 0.8 denotes a strong correlation between the antibody array and r2 ≤ 0.5 denotes a weak correlation between the gene expression values obtained from independent senescent fibroblast strains.
(D) Specific ELISAs for GCP-2 (top) ENA-78 (middle) and RANTES (bottom) were used to assess the concentration of these chemokines in the supernatants (CM) of non senescent fibroblasts (FF95, CPD 16) and replicative senescent fibroblasts (FF95, CPD 68) at the indicated time points of incubation. The specific chemokines were expressed as pg/105 fibroblasts. All data are mean ± s.e.m.. All experiments were repeated 3 times with two independent fibroblast lines. *P<0.01, ***P<0.0001 by Student´s t-test.
(E) Senescent fibroblasts isolated from skin of old individuals express high levels of GCP-2 and ENA-78. Skin biopsies from 3 independent healthy human donors (86-year-old female, 86-year-old male and 80-year-old female) were obtained and subjected to a MACS whole skin dissociation protocol (# Cat 130-101-540, Milteny Biotec) as described. Thereafter, p16INK4a (CDKN2A) human specific monoclonal antibody (# Cat 130-128- 162, Milteny Biotech) and anti-IgG microbeads human specifc (# Cat 130-047-501, Milteny Biotech) were used to pull down p16INK4a+ senescent fibroblasts from whole skin. RNA was isolated as described earlier and subjected to NGS using RNA library prep using NEBNext Ultra II Directional RNA library prep kit for Illumina (#Cat E7760S, NEB) with Unique Dual Index adaptors (#Cat E7416S, NEB). The volcano plot (left) shows that the transcript CXCL6 (GCP-2) is 5.6/5.4 and CXCL5 (ENA78) is 5.4/4.28-fold upregulated in in vivo aged (senescent) fibroblasts isolated from the skin of old human adults as opposed to no GCP-2 and ENA expression in young (non-senescent) fibroblasts from the skin of young adults. These data indicate that in vitro and in vivo generated senescent fibroblasts show higher expression of GCP-2. This can be also observed in the heatmap (right panel) where CXCL6/ CXCL5 depicts high expression (shown in red) as compared to low expressing transcript region, shown in blue. This data set can be downloaded from NIH/NCBI data repository SRA with submission Nr. SUB14675011, Bioproject Nr. PRJNA1150800.

## Slide 12
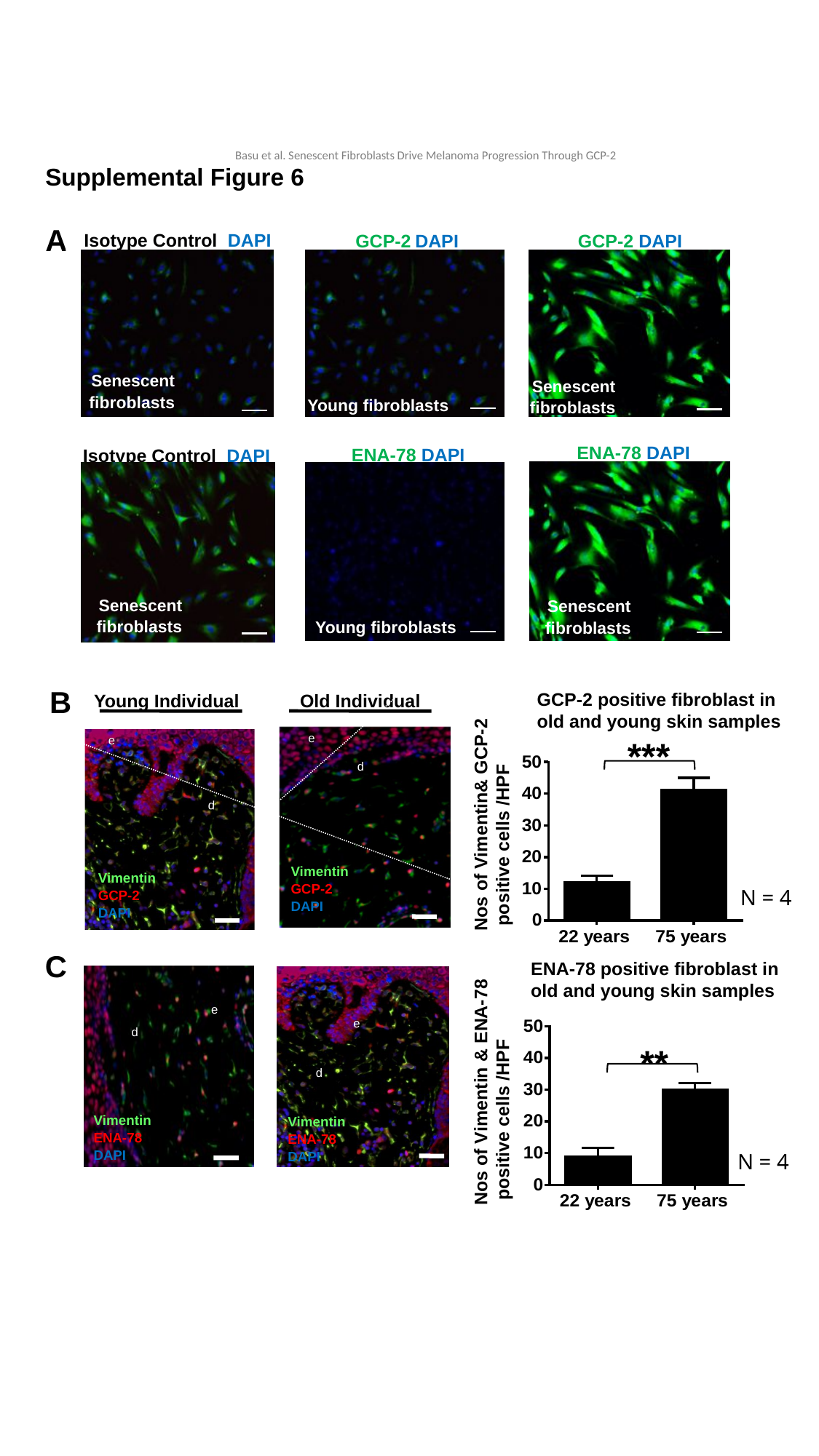

Basu et al. Senescent Fibroblasts Drive Melanoma Progression Through GCP-2
Supplemental Figure 6
A
Isotype Control DAPI
GCP-2 DAPI
GCP-2 DAPI
Senescent fibroblasts
Young fibroblasts
ENA-78 DAPI
ENA-78 DAPI
Senescent fibroblasts
Senescent fibroblasts
Young fibroblasts
Senescent fibroblasts
Isotype Control DAPI
B
Young Individual
Old Individual
 GCP-2 positive fibroblast in
 old and young skin samples
***
Nos of Vimentin& GCP-2
 positive cells /HPF
 ENA-78 positive fibroblast in
 old and young skin samples
**
Nos of Vimentin & ENA-78
 positive cells /HPF
e
e
d
Vimentin
GCP-2
DAPI
d
Vimentin
GCP-2
DAPI
N = 4
C
e
e
d
d
Vimentin
ENA-78
DAPI
Vimentin
ENA-78
DAPI
N = 4

## Slide 13
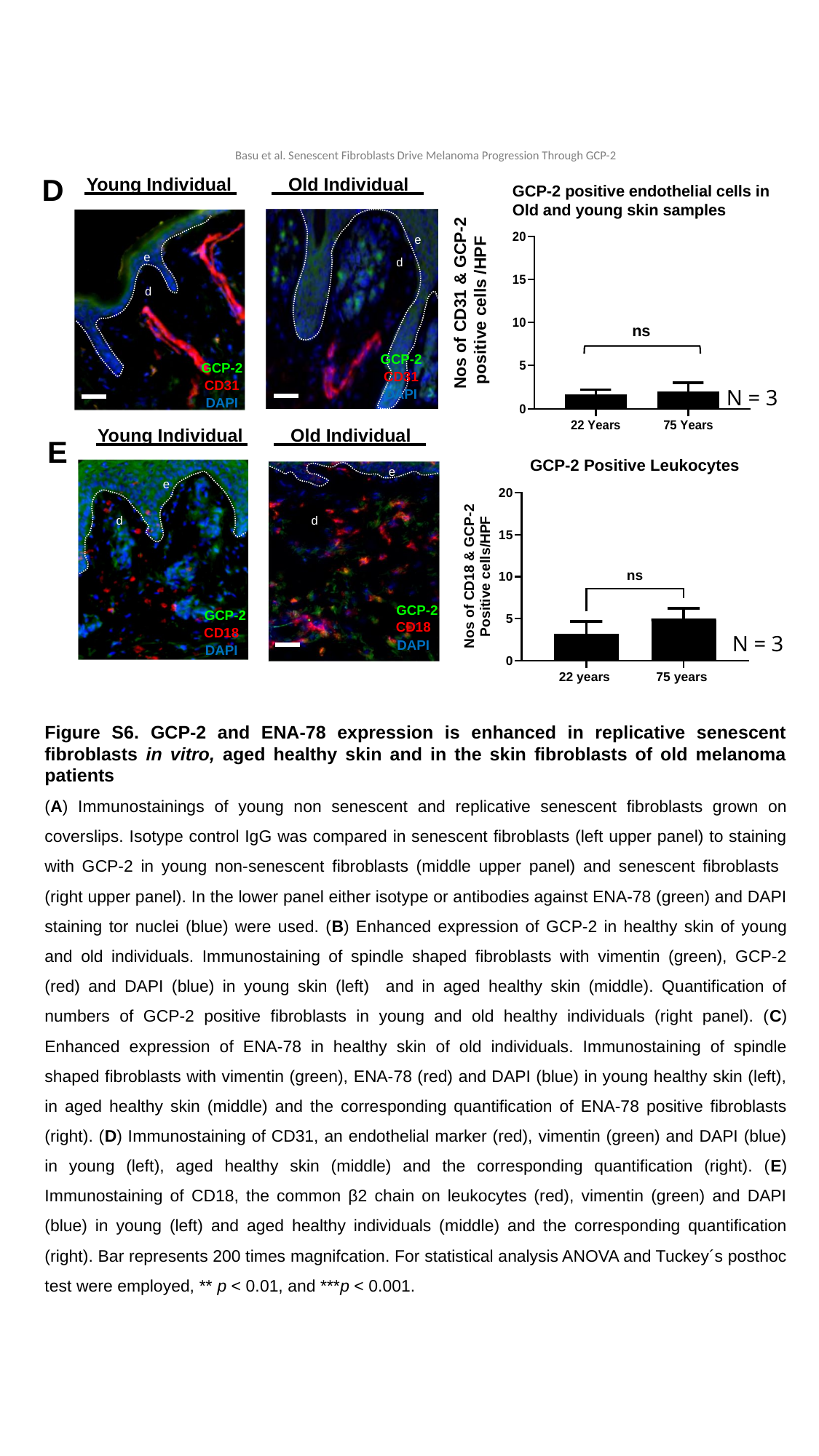

Basu et al. Senescent Fibroblasts Drive Melanoma Progression Through GCP-2
D
Young Individual
Old Individual
e
d
e
d
GCP-2
CD31
DAPI
Young Individual
Old Individual
E
e
e
d
d
 GCP-2
CD18
DAPI
 GCP-2
CD18
DAPI
GCP-2 positive endothelial cells in
Old and young skin samples
ns
Nos of CD31 & GCP-2
 positive cells /HPF
GCP-2
CD31
DAPI
N = 3
N = 3
Figure S6. GCP-2 and ENA-78 expression is enhanced in replicative senescent fibroblasts in vitro, aged healthy skin and in the skin fibroblasts of old melanoma patients
(A) Immunostainings of young non senescent and replicative senescent fibroblasts grown on coverslips. Isotype control IgG was compared in senescent fibroblasts (left upper panel) to staining with GCP-2 in young non-senescent fibroblasts (middle upper panel) and senescent fibroblasts (right upper panel). In the lower panel either isotype or antibodies against ENA-78 (green) and DAPI staining tor nuclei (blue) were used. (B) Enhanced expression of GCP-2 in healthy skin of young and old individuals. Immunostaining of spindle shaped fibroblasts with vimentin (green), GCP-2 (red) and DAPI (blue) in young skin (left) and in aged healthy skin (middle). Quantification of numbers of GCP-2 positive fibroblasts in young and old healthy individuals (right panel). (C) Enhanced expression of ENA-78 in healthy skin of old individuals. Immunostaining of spindle shaped fibroblasts with vimentin (green), ENA-78 (red) and DAPI (blue) in young healthy skin (left), in aged healthy skin (middle) and the corresponding quantification of ENA-78 positive fibroblasts (right). (D) Immunostaining of CD31, an endothelial marker (red), vimentin (green) and DAPI (blue) in young (left), aged healthy skin (middle) and the corresponding quantification (right). (E) Immunostaining of CD18, the common β2 chain on leukocytes (red), vimentin (green) and DAPI (blue) in young (left) and aged healthy individuals (middle) and the corresponding quantification (right). Bar represents 200 times magnifcation. For statistical analysis ANOVA and Tuckey´s posthoc test were employed, ** p < 0.01, and ***p < 0.001.

## Slide 14
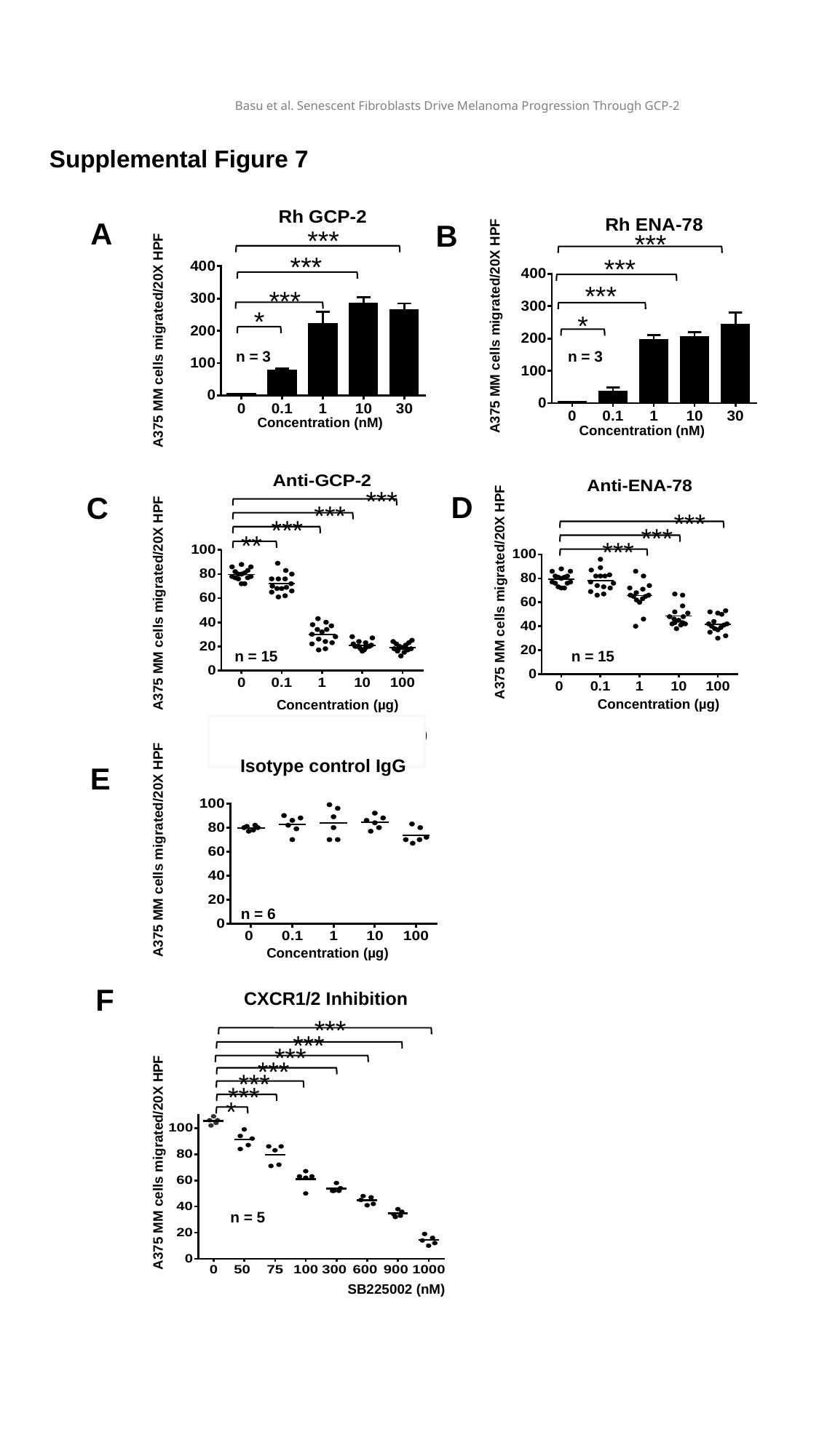

Basu et al. Senescent Fibroblasts Drive Melanoma Progression Through GCP-2
Supplemental Figure 7
A
B
***
***
***
***
***
***
*
*
A375 MM cells migrated/20X HPF
A375 MM cells migrated/20X HPF
n = 3
n = 3
 Concentration (nM)
 Concentration (nM)
***
D
C
***
***
***
***
**
***
A375 MM cells migrated/20X HPF
A375 MM cells migrated/20X HPF
n = 15
n = 15
Concentration (µg)
Concentration (µg)
Isotype control IgG
E
A375 MM cells migrated/20X HPF
n = 6
Concentration (µg)
F
CXCR1/2 Inhibition
***
***
***
***
***
***
*
A375 MM cells migrated/20X HPF
n = 5
SB225002 (nM)

## Slide 15
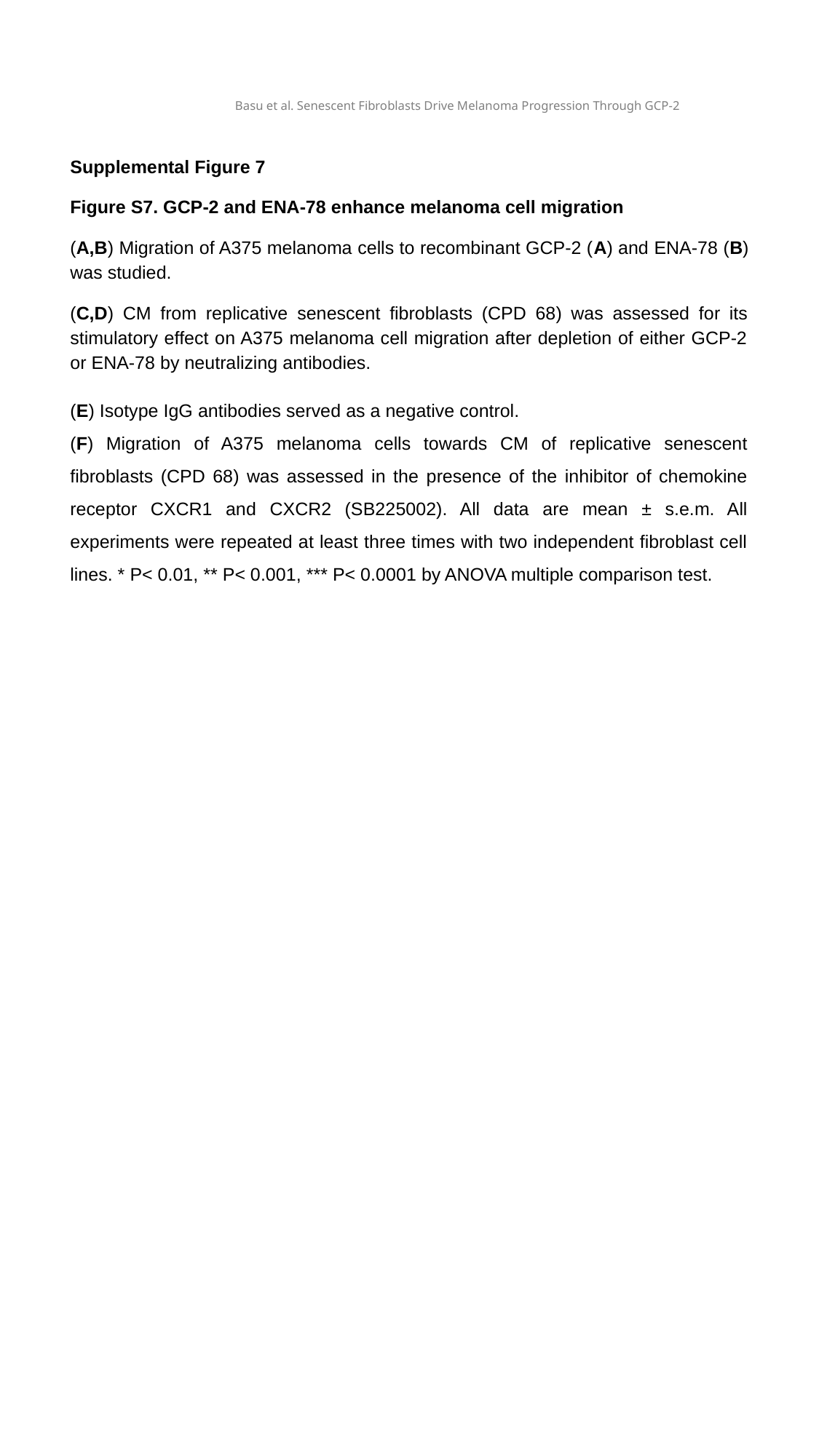

Basu et al. Senescent Fibroblasts Drive Melanoma Progression Through GCP-2
Supplemental Figure 7
Figure S7. GCP-2 and ENA-78 enhance melanoma cell migration
(A,B) Migration of A375 melanoma cells to recombinant GCP-2 (A) and ENA-78 (B) was studied.
(C,D) CM from replicative senescent fibroblasts (CPD 68) was assessed for its stimulatory effect on A375 melanoma cell migration after depletion of either GCP-2 or ENA-78 by neutralizing antibodies.
(E) Isotype IgG antibodies served as a negative control.
(F) Migration of A375 melanoma cells towards CM of replicative senescent fibroblasts (CPD 68) was assessed in the presence of the inhibitor of chemokine receptor CXCR1 and CXCR2 (SB225002). All data are mean ± s.e.m. All experiments were repeated at least three times with two independent fibroblast cell lines. * P< 0.01, ** P< 0.001, *** P< 0.0001 by ANOVA multiple comparison test.

## Slide 16
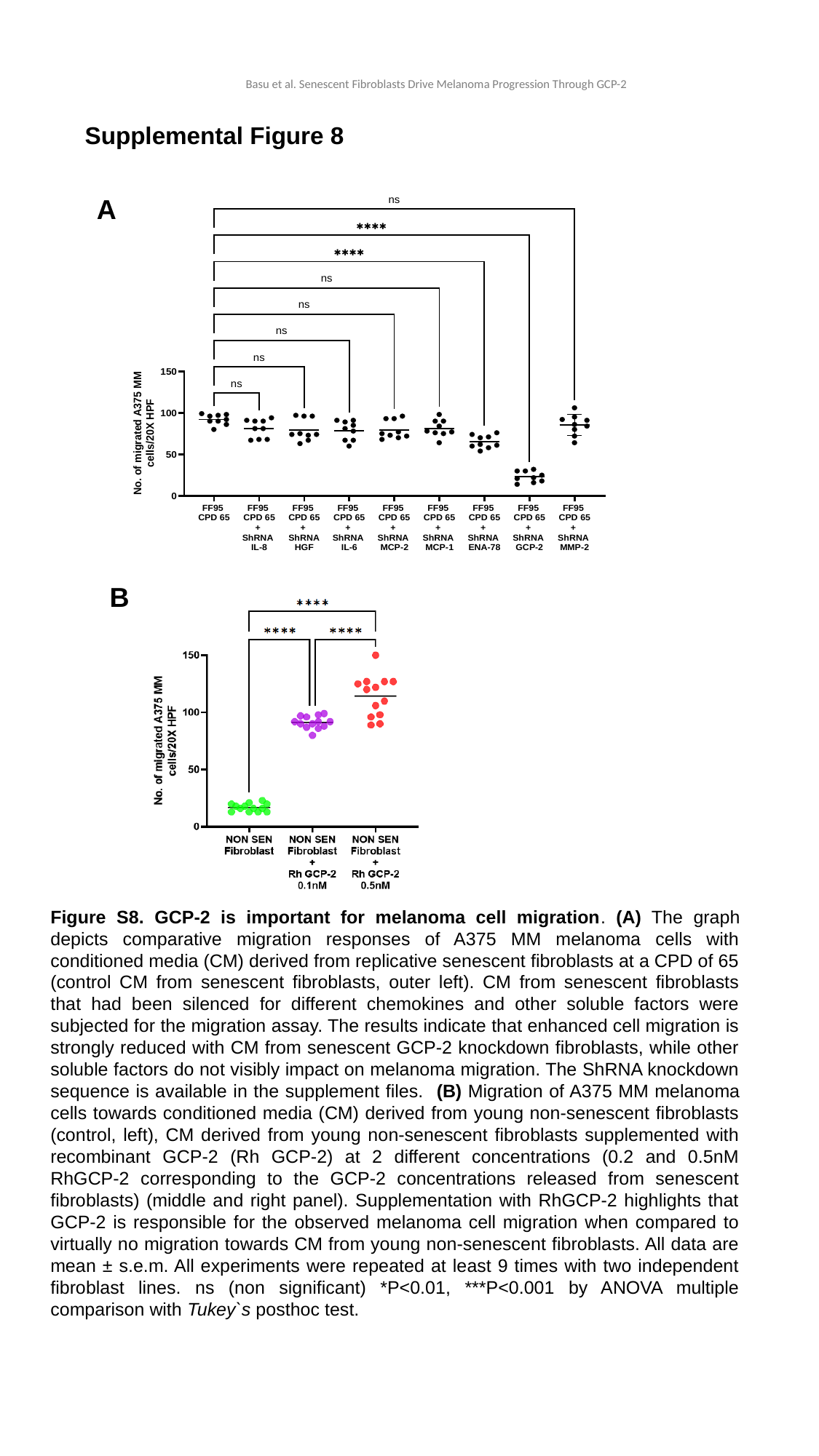

Basu et al. Senescent Fibroblasts Drive Melanoma Progression Through GCP-2
Supplemental Figure 8
A
B
Figure S8. GCP-2 is important for melanoma cell migration. (A) The graph depicts comparative migration responses of A375 MM melanoma cells with conditioned media (CM) derived from replicative senescent fibroblasts at a CPD of 65 (control CM from senescent fibroblasts, outer left). CM from senescent fibroblasts that had been silenced for different chemokines and other soluble factors were subjected for the migration assay. The results indicate that enhanced cell migration is strongly reduced with CM from senescent GCP-2 knockdown fibroblasts, while other soluble factors do not visibly impact on melanoma migration. The ShRNA knockdown sequence is available in the supplement files. (B) Migration of A375 MM melanoma cells towards conditioned media (CM) derived from young non-senescent fibroblasts (control, left), CM derived from young non-senescent fibroblasts supplemented with recombinant GCP-2 (Rh GCP-2) at 2 different concentrations (0.2 and 0.5nM RhGCP-2 corresponding to the GCP-2 concentrations released from senescent fibroblasts) (middle and right panel). Supplementation with RhGCP-2 highlights that GCP-2 is responsible for the observed melanoma cell migration when compared to virtually no migration towards CM from young non-senescent fibroblasts. All data are mean ± s.e.m. All experiments were repeated at least 9 times with two independent fibroblast lines. ns (non significant) *P<0.01, ***P<0.001 by ANOVA multiple comparison with Tukey`s posthoc test.

## Slide 17
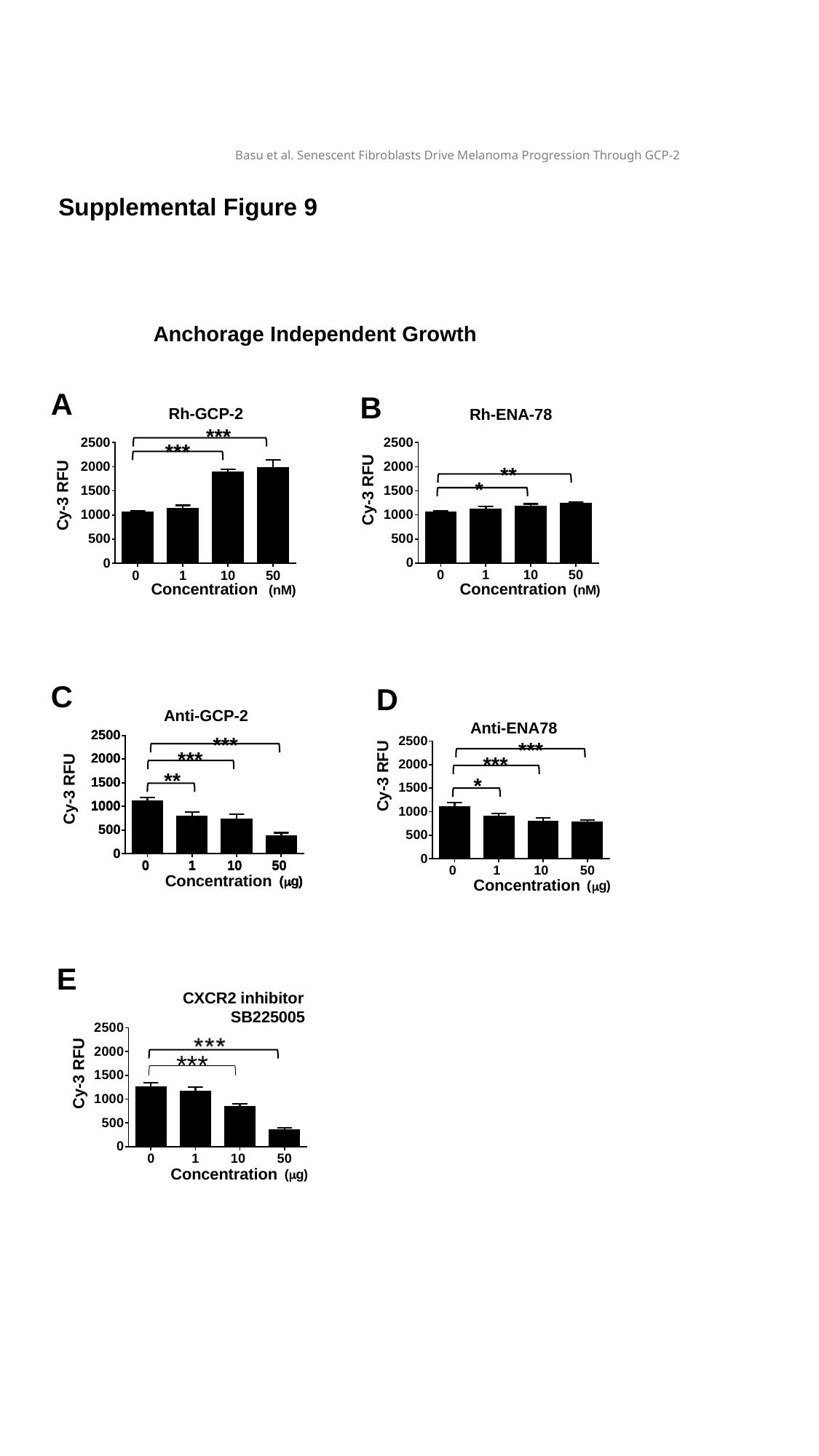

Basu et al. Senescent Fibroblasts Drive Melanoma Progression Through GCP-2
Supplemental Figure 9
Anchorage Independent Growth
A
B
Rh-GCP-2
Rh-ENA-78
***
***
**
*
Cy-3 RFU
Cy-3 RFU
Concentration
Concentration
C
D
Anti-GCP-2
Anti-ENA78
***
***
***
***
**
Cy-3 RFU
*
Cy-3 RFU
Concentration
Concentration
E
 CXCR2 inhibitor SB225005
***
Cy-3 RFU
Concentration

## Slide 18
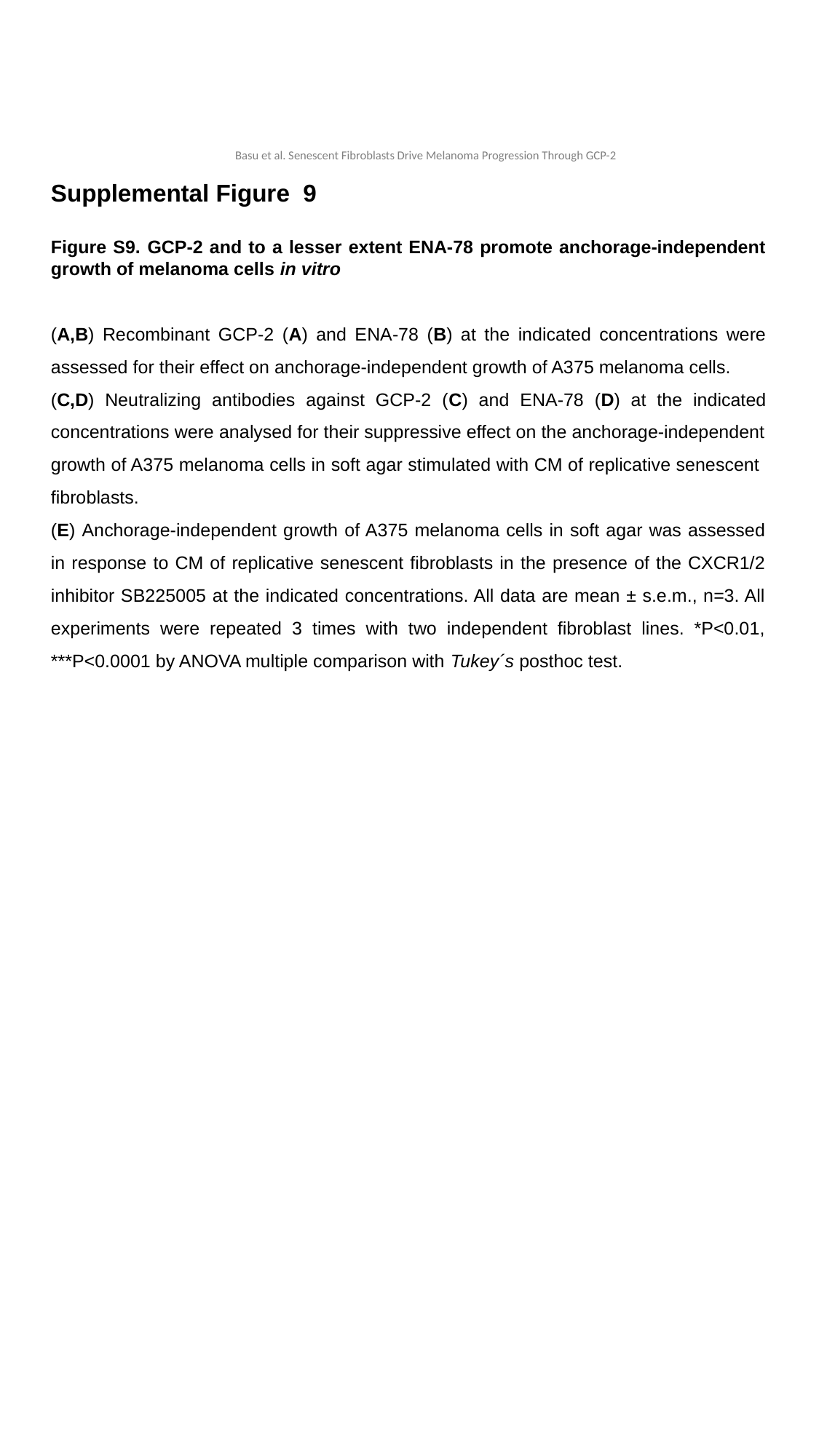

Basu et al. Senescent Fibroblasts Drive Melanoma Progression Through GCP-2
Supplemental Figure 9
Figure S9. GCP-2 and to a lesser extent ENA-78 promote anchorage-independent growth of melanoma cells in vitro
(A,B) Recombinant GCP-2 (A) and ENA-78 (B) at the indicated concentrations were assessed for their effect on anchorage-independent growth of A375 melanoma cells.
(C,D) Neutralizing antibodies against GCP-2 (C) and ENA-78 (D) at the indicated concentrations were analysed for their suppressive effect on the anchorage-independent growth of A375 melanoma cells in soft agar stimulated with CM of replicative senescent fibroblasts.
(E) Anchorage-independent growth of A375 melanoma cells in soft agar was assessed in response to CM of replicative senescent fibroblasts in the presence of the CXCR1/2 inhibitor SB225005 at the indicated concentrations. All data are mean ± s.e.m., n=3. All experiments were repeated 3 times with two independent fibroblast lines. *P<0.01, ***P<0.0001 by ANOVA multiple comparison with Tukey´s posthoc test.

## Slide 19
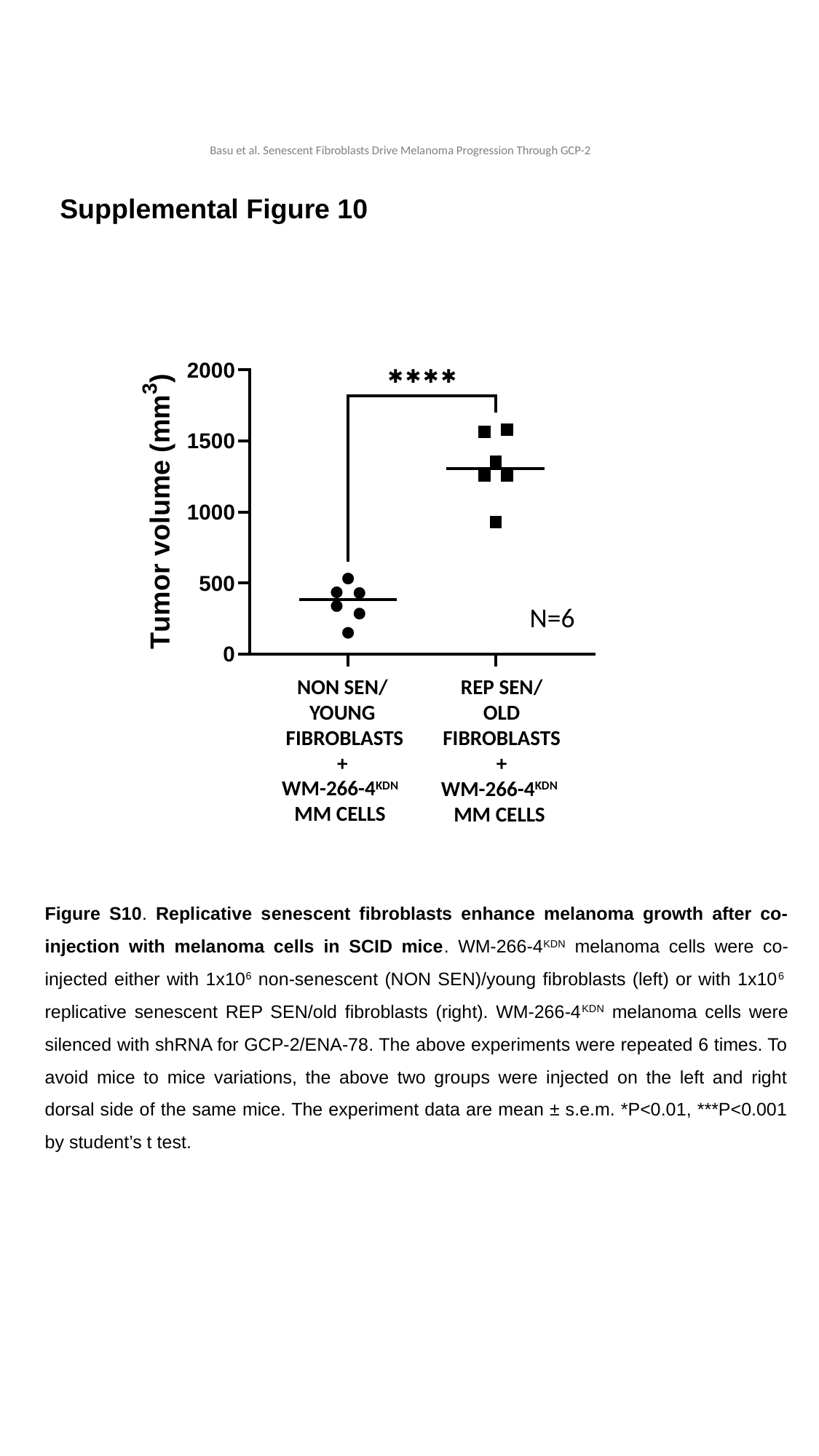

Basu et al. Senescent Fibroblasts Drive Melanoma Progression Through GCP-2
Supplemental Figure 10
N=6
NON SEN/
YOUNG
 FIBROBLASTS
+
WM-266-4KDN
MM CELLS
REP SEN/
OLD
FIBROBLASTS
+
WM-266-4KDN
MM CELLS
Figure S10. Replicative senescent fibroblasts enhance melanoma growth after co-injection with melanoma cells in SCID mice. WM-266-4KDN melanoma cells were co-injected either with 1x10^6^ non-senescent (NON SEN)/young fibroblasts (left) or with 1x10^6^ replicative senescent REP SEN/old fibroblasts (right). WM-266-4KDN melanoma cells were silenced with shRNA for GCP-2/ENA-78. The above experiments were repeated 6 times. To avoid mice to mice variations, the above two groups were injected on the left and right dorsal side of the same mice. The experiment data are mean ± s.e.m. *P<0.01, ***P<0.001 by student’s t test.

## Slide 20
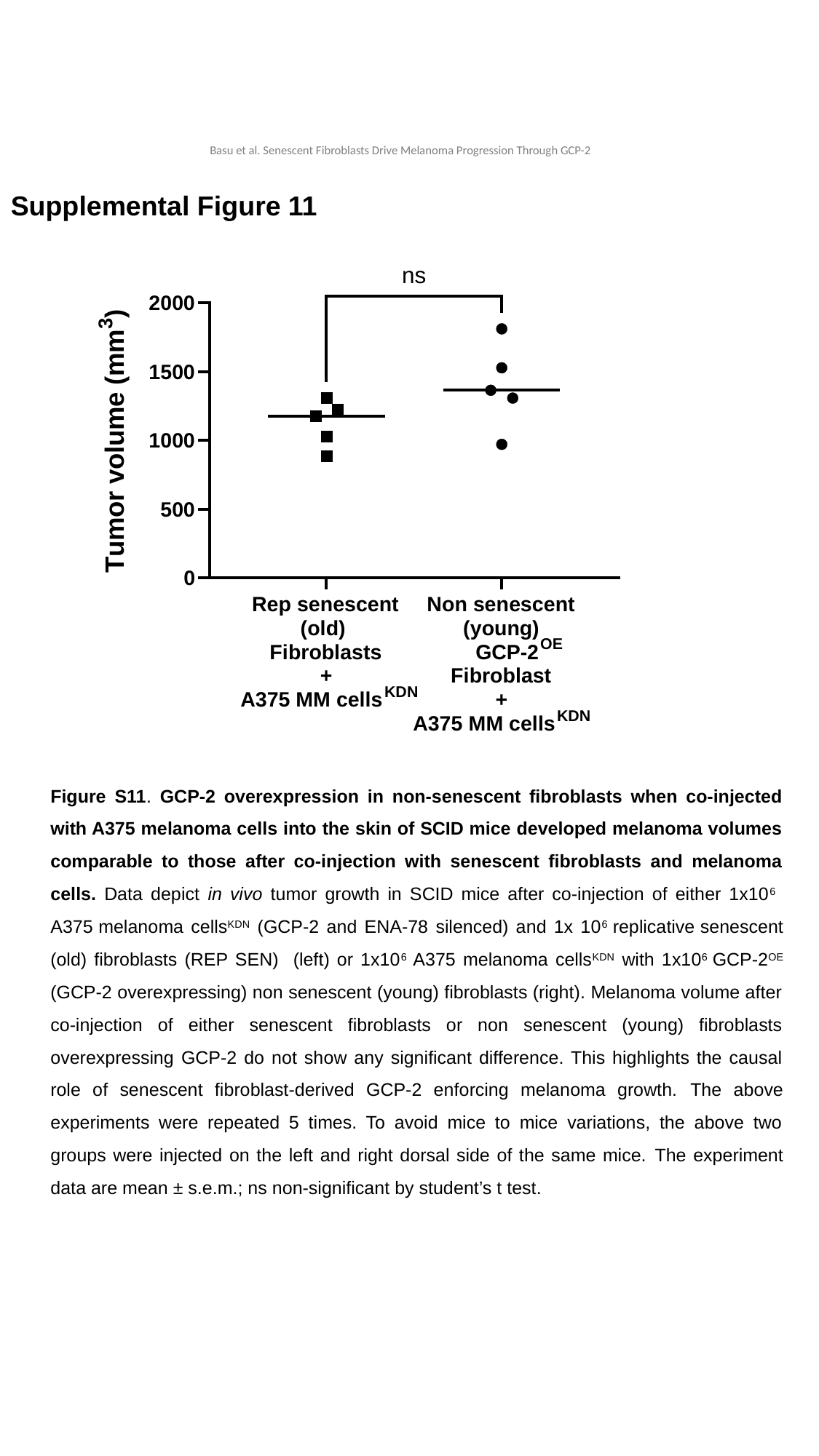

Basu et al. Senescent Fibroblasts Drive Melanoma Progression Through GCP-2
Supplemental Figure 11
Figure S11. GCP-2 overexpression in non-senescent fibroblasts when co-injected with A375 melanoma cells into the skin of SCID mice developed melanoma volumes comparable to those after co-injection with senescent fibroblasts and melanoma cells. Data depict in vivo tumor growth in SCID mice after co-injection of either 1x10^6^ A375 melanoma cellsKDN (GCP-2 and ENA-78 silenced) and 1x 10^6^ replicative senescent (old) fibroblasts (REP SEN) (left) or 1x10^6^ A375 melanoma cellsKDN with 1x10^6^ GCP-2OE (GCP-2 overexpressing) non senescent (young) fibroblasts (right). Melanoma volume after co-injection of either senescent fibroblasts or non senescent (young) fibroblasts overexpressing GCP-2 do not show any significant difference. This highlights the causal role of senescent fibroblast-derived GCP-2 enforcing melanoma growth. The above experiments were repeated 5 times. To avoid mice to mice variations, the above two groups were injected on the left and right dorsal side of the same mice. The experiment data are mean ± s.e.m.; ns non-significant by student’s t test.

## Slide 21
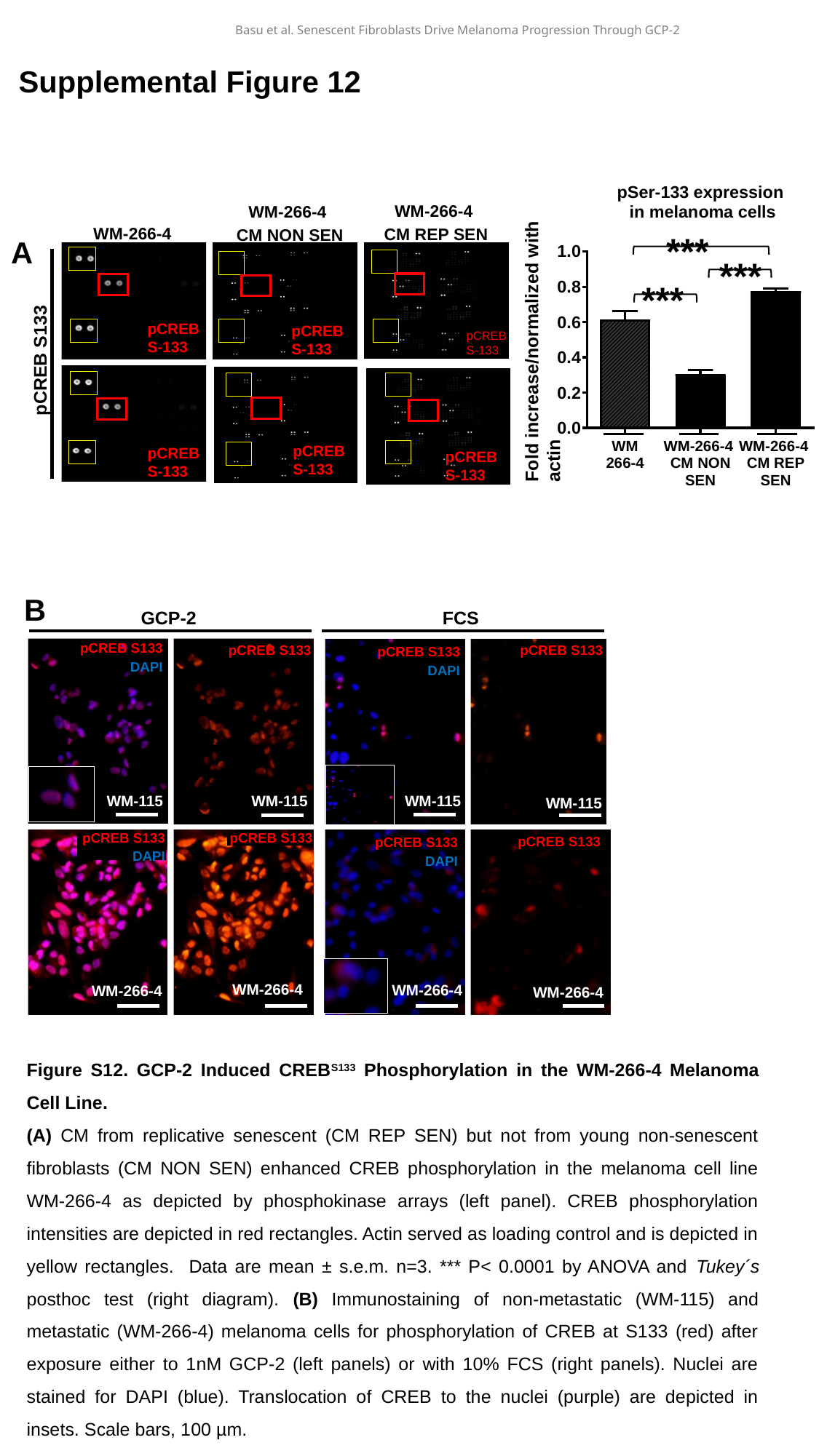

Basu et al. Senescent Fibroblasts Drive Melanoma Progression Through GCP-2
Supplemental Figure 12
***
***
***
Fold increase/normalized with actin
WM-266-4
CM REP SEN
WM-266-4
CM NON SEN
WM-266-4
A
pCREB
S-133
pCREB
S-133
pCREB
S-133
pCREB S133
pCREB
S-133
pCREB
S-133
pCREB
S-133
B
GCP-2
FCS
pCREB S133
DAPI
pCREB S133
pCREB S133
pCREB S133
DAPI
WM-115
WM-115
WM-115
WM-115
pCREB S133
DAPI
pCREB S133
pCREB S133
pCREB S133
DAPI
WM-266-4
WM-266-4
WM-266-4
WM-266-4
Figure S12. GCP-2 Induced CREBS133 Phosphorylation in the WM-266-4 Melanoma Cell Line.
(A) CM from replicative senescent (CM REP SEN) but not from young non-senescent fibroblasts (CM NON SEN) enhanced CREB phosphorylation in the melanoma cell line WM-266-4 as depicted by phosphokinase arrays (left panel). CREB phosphorylation intensities are depicted in red rectangles. Actin served as loading control and is depicted in yellow rectangles. Data are mean ± s.e.m. n=3. *** P< 0.0001 by ANOVA and Tukey´s posthoc test (right diagram). (B) Immunostaining of non-metastatic (WM-115) and metastatic (WM-266-4) melanoma cells for phosphorylation of CREB at S133 (red) after exposure either to 1nM GCP-2 (left panels) or with 10% FCS (right panels). Nuclei are stained for DAPI (blue). Translocation of CREB to the nuclei (purple) are depicted in insets. Scale bars, 100 µm.
.

## Slide 22
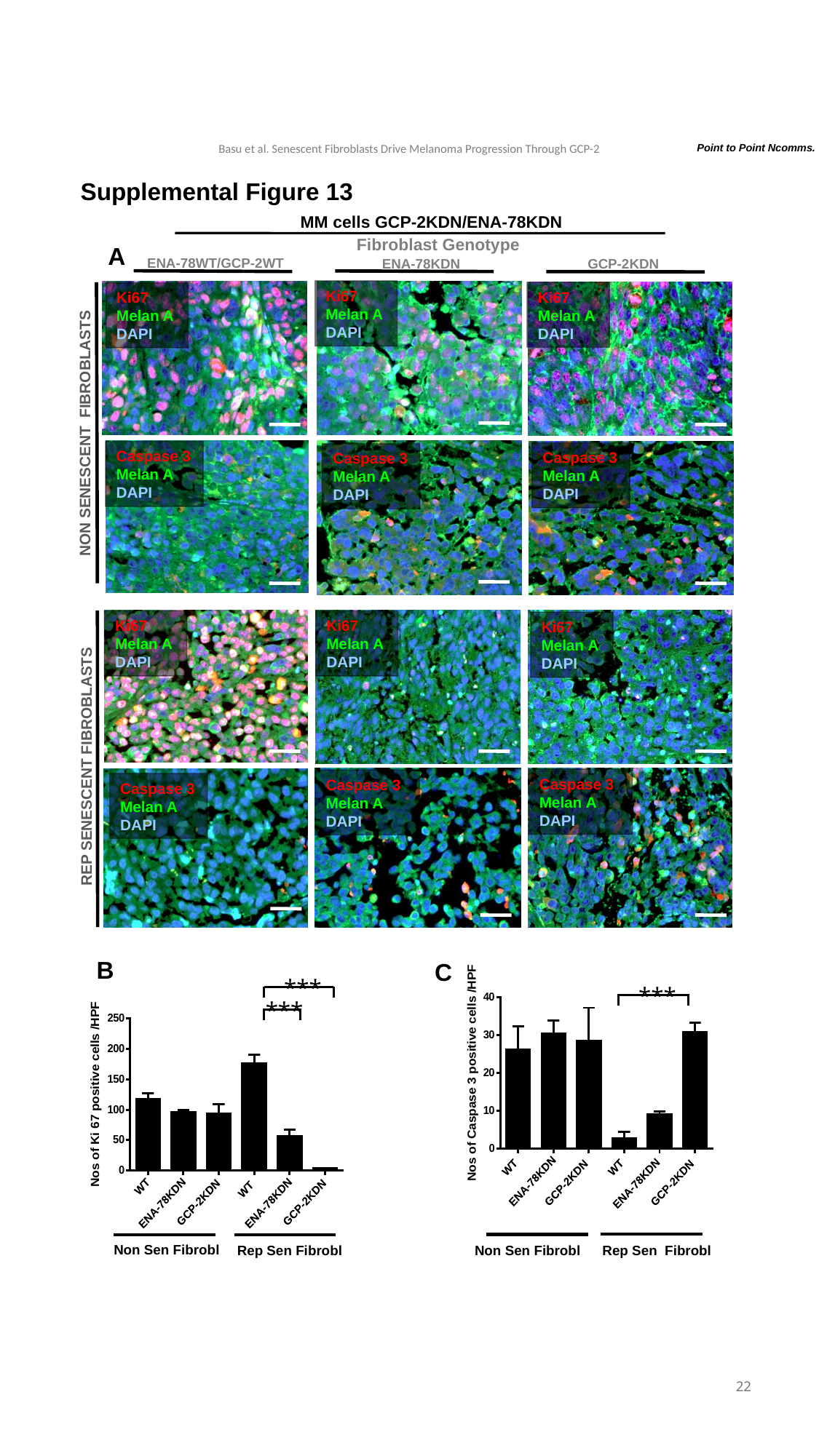

Point to Point Ncomms.
Basu et al. Senescent Fibroblasts Drive Melanoma Progression Through GCP-2
Supplemental Figure 13
MM cells GCP-2KDN/ENA-78KDN
Fibroblast Genotype
A
ENA-78WT/GCP-2WT
GCP-2KDN
ENA-78KDN
Ki67
Melan A
DAPI
Ki67
Melan A
DAPI
Ki67
Melan A
DAPI
NON SENESCENT FIBROBLASTS
Caspase 3
Melan A
DAPI
Caspase 3
Melan A
DAPI
Caspase 3
Melan A
DAPI
Ki67
Melan A
DAPI
Ki67
Melan A
DAPI
Ki67
Melan A
DAPI
REP SENESCENT FIBROBLASTS
Caspase 3
Melan A
DAPI
Caspase 3
Melan A
DAPI
Caspase 3
Melan A
DAPI
B
C
***
***
***
Non Sen Fibrobl
Rep Sen Fibrobl
Non Sen Fibrobl
Rep Sen Fibrobl
22

## Slide 23
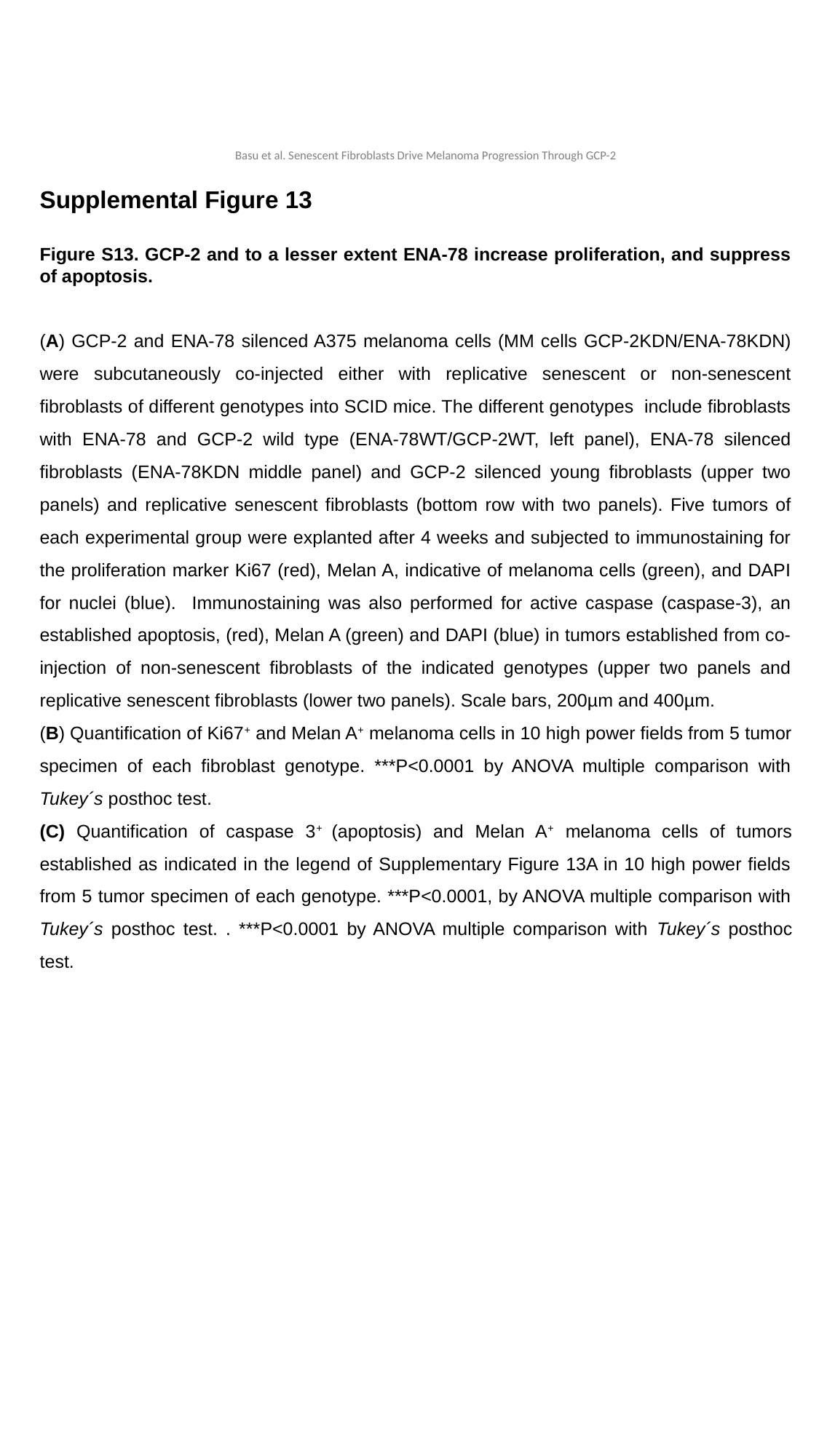

Basu et al. Senescent Fibroblasts Drive Melanoma Progression Through GCP-2
Supplemental Figure 13
Figure S13. GCP-2 and to a lesser extent ENA-78 increase proliferation, and suppress of apoptosis.
(A) GCP-2 and ENA-78 silenced A375 melanoma cells (MM cells GCP-2KDN/ENA-78KDN) were subcutaneously co-injected either with replicative senescent or non-senescent fibroblasts of different genotypes into SCID mice. The different genotypes include fibroblasts with ENA-78 and GCP-2 wild type (ENA-78WT/GCP-2WT, left panel), ENA-78 silenced fibroblasts (ENA-78KDN middle panel) and GCP-2 silenced young fibroblasts (upper two panels) and replicative senescent fibroblasts (bottom row with two panels). Five tumors of each experimental group were explanted after 4 weeks and subjected to immunostaining for the proliferation marker Ki67 (red), Melan A, indicative of melanoma cells (green), and DAPI for nuclei (blue). Immunostaining was also performed for active caspase (caspase-3), an established apoptosis, (red), Melan A (green) and DAPI (blue) in tumors established from co-injection of non-senescent fibroblasts of the indicated genotypes (upper two panels and replicative senescent fibroblasts (lower two panels). Scale bars, 200µm and 400µm.
(B) Quantification of Ki67+ and Melan A+ melanoma cells in 10 high power fields from 5 tumor specimen of each fibroblast genotype. ***P<0.0001 by ANOVA multiple comparison with Tukey´s posthoc test.
(C) Quantification of caspase 3+ (apoptosis) and Melan A+ melanoma cells of tumors established as indicated in the legend of Supplementary Figure 13A in 10 high power fields from 5 tumor specimen of each genotype. ***P<0.0001, by ANOVA multiple comparison with Tukey´s posthoc test. . ***P<0.0001 by ANOVA multiple comparison with Tukey´s posthoc test.

## Slide 24
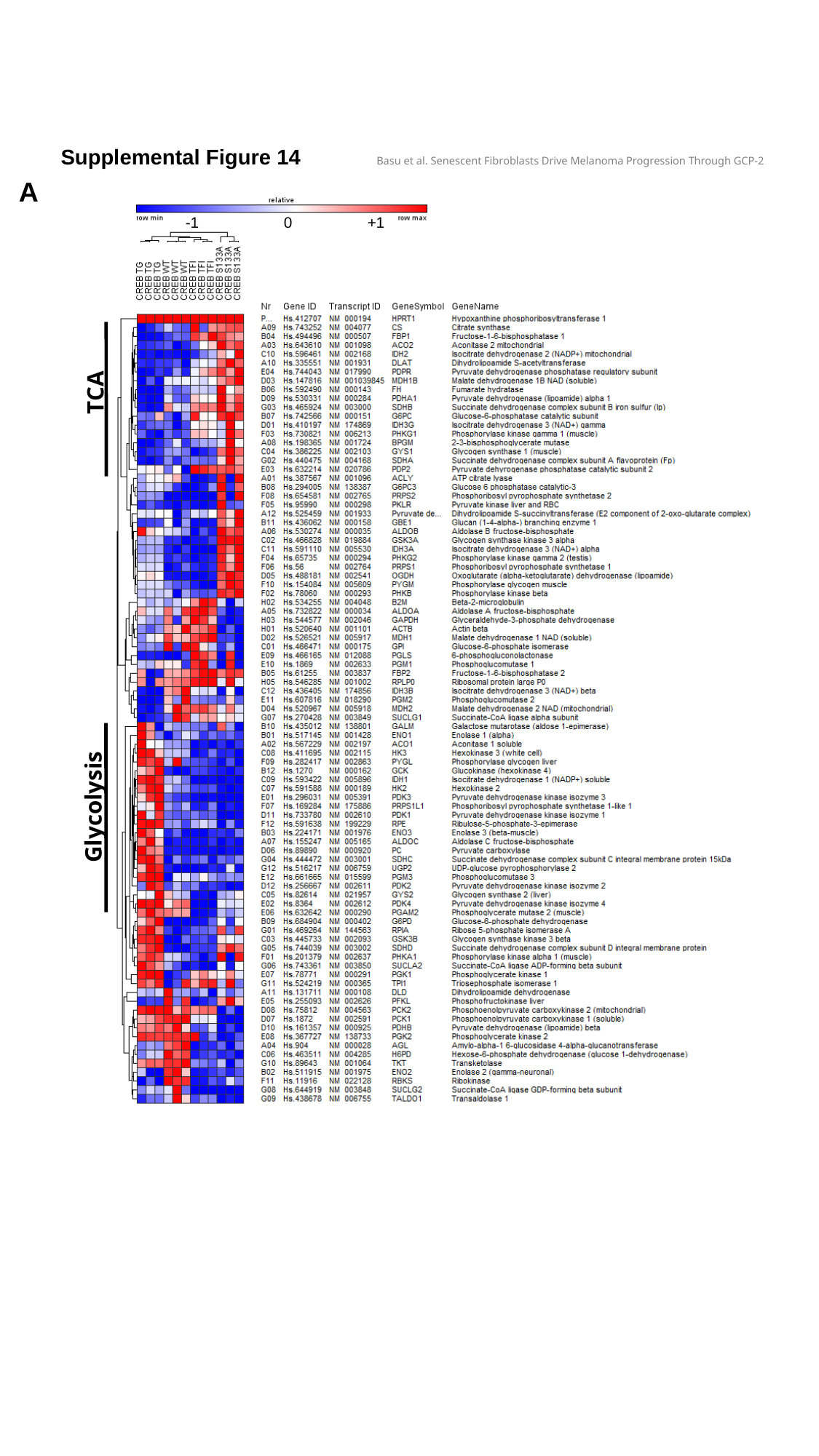

Supplemental Figure 14
Basu et al. Senescent Fibroblasts Drive Melanoma Progression Through GCP-2
A
-1
0
+1
Glucose Metabolism Gene Expression
TCA
Glycolysis

## Slide 25
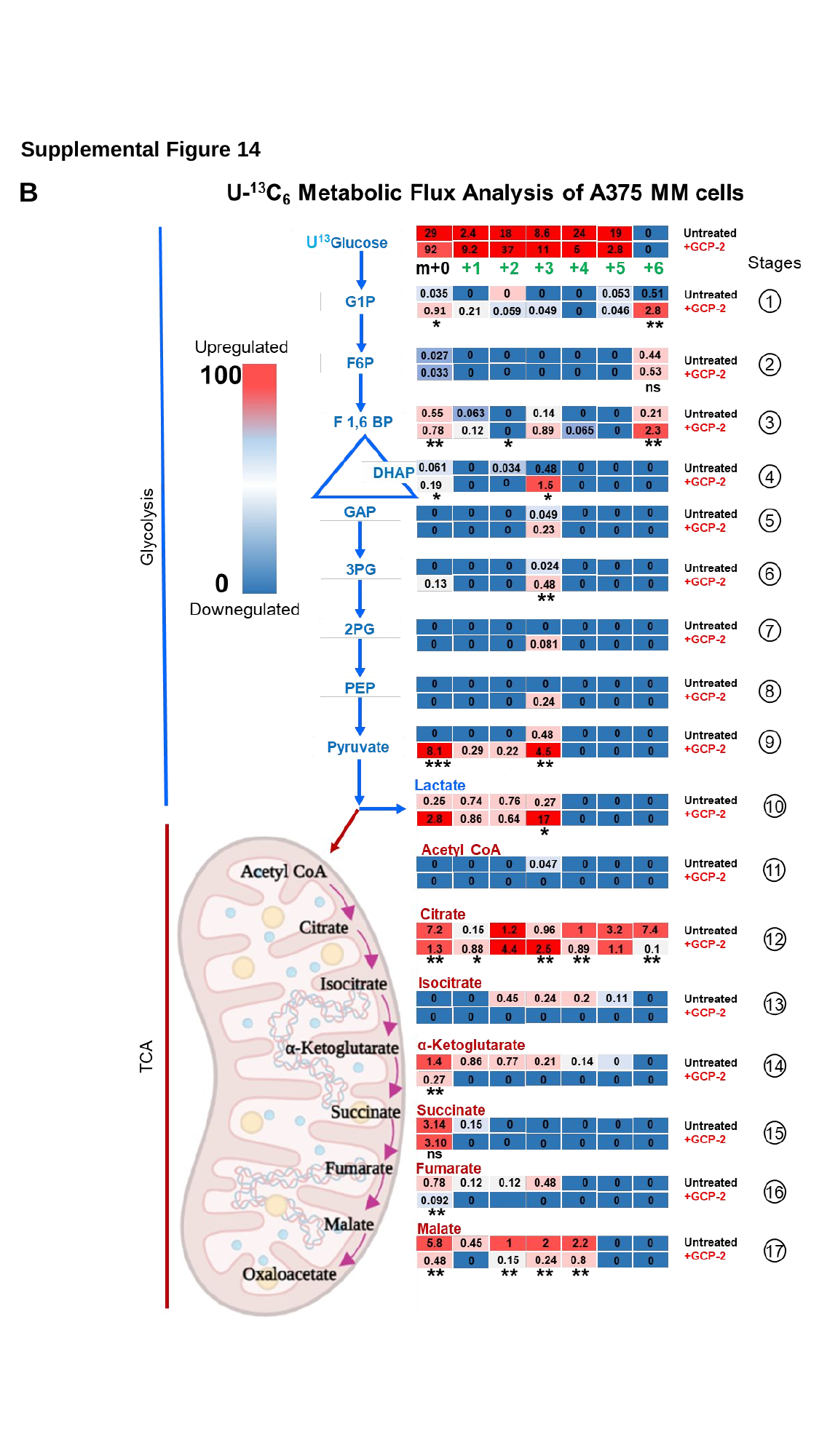

Supplemental Figure 14
B

## Slide 26
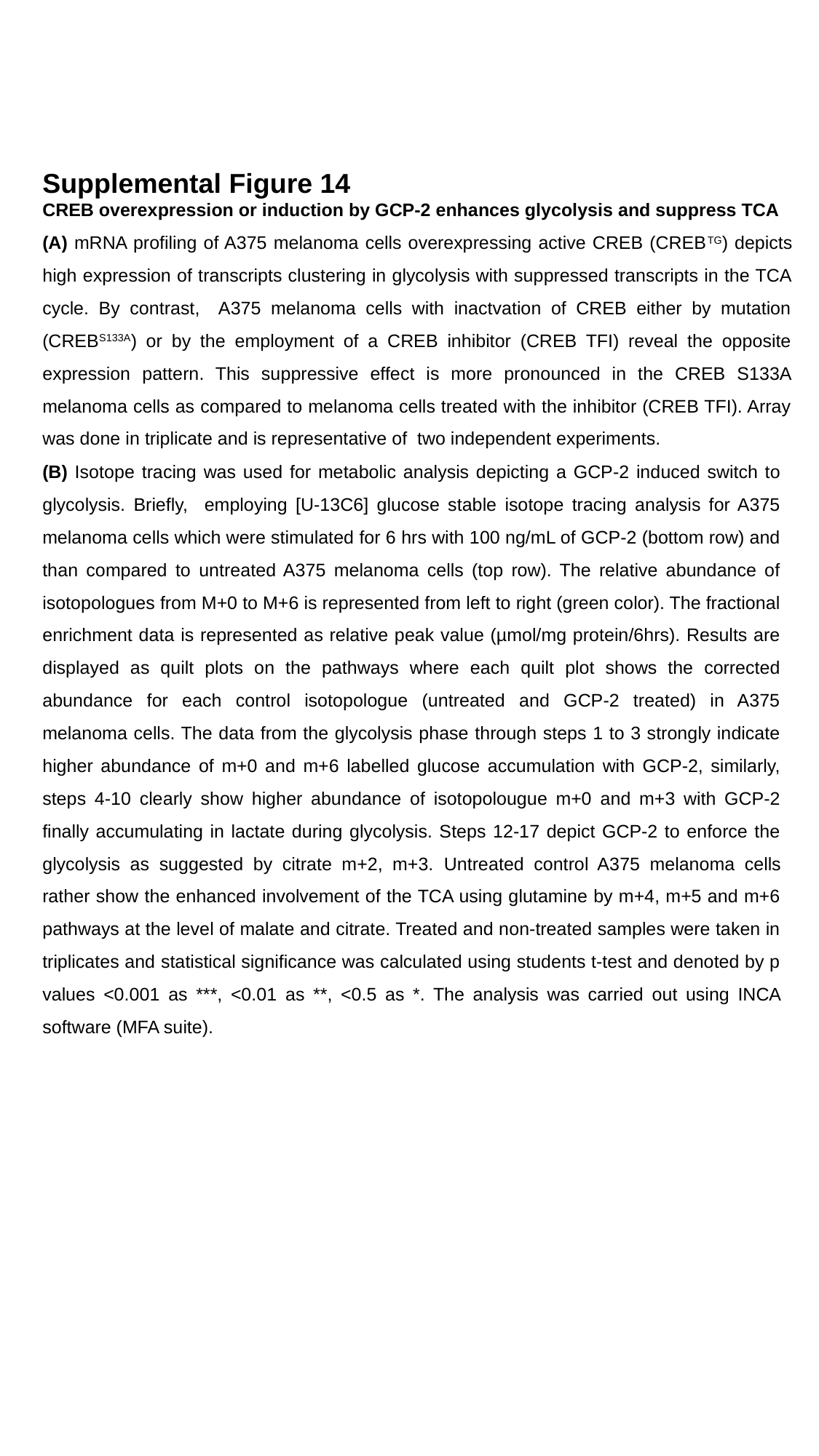

Supplemental Figure 14
CREB overexpression or induction by GCP-2 enhances glycolysis and suppress TCA
(A) mRNA profiling of A375 melanoma cells overexpressing active CREB (CREBTG) depicts high expression of transcripts clustering in glycolysis with suppressed transcripts in the TCA cycle. By contrast, A375 melanoma cells with inactvation of CREB either by mutation (CREBS133A) or by the employment of a CREB inhibitor (CREB TFI) reveal the opposite expression pattern. This suppressive effect is more pronounced in the CREB S133A melanoma cells as compared to melanoma cells treated with the inhibitor (CREB TFI). Array was done in triplicate and is representative of two independent experiments.
(B) Isotope tracing was used for metabolic analysis depicting a GCP-2 induced switch to glycolysis. Briefly, employing [U-13C6] glucose stable isotope tracing analysis for A375 melanoma cells which were stimulated for 6 hrs with 100 ng/mL of GCP-2 (bottom row) and than compared to untreated A375 melanoma cells (top row). The relative abundance of isotopologues from M+0 to M+6 is represented from left to right (green color). The fractional enrichment data is represented as relative peak value (µmol/mg protein/6hrs). Results are displayed as quilt plots on the pathways where each quilt plot shows the corrected abundance for each control isotopologue (untreated and GCP-2 treated) in A375 melanoma cells. The data from the glycolysis phase through steps 1 to 3 strongly indicate higher abundance of m+0 and m+6 labelled glucose accumulation with GCP-2, similarly, steps 4-10 clearly show higher abundance of isotopolougue m+0 and m+3 with GCP-2 finally accumulating in lactate during glycolysis. Steps 12-17 depict GCP-2 to enforce the glycolysis as suggested by citrate m+2, m+3. Untreated control A375 melanoma cells rather show the enhanced involvement of the TCA using glutamine by m+4, m+5 and m+6 pathways at the level of malate and citrate. Treated and non-treated samples were taken in triplicates and statistical significance was calculated using students t-test and denoted by p values <0.001 as ***, <0.01 as **, <0.5 as *. The analysis was carried out using INCA software (MFA suite).

## Slide 27
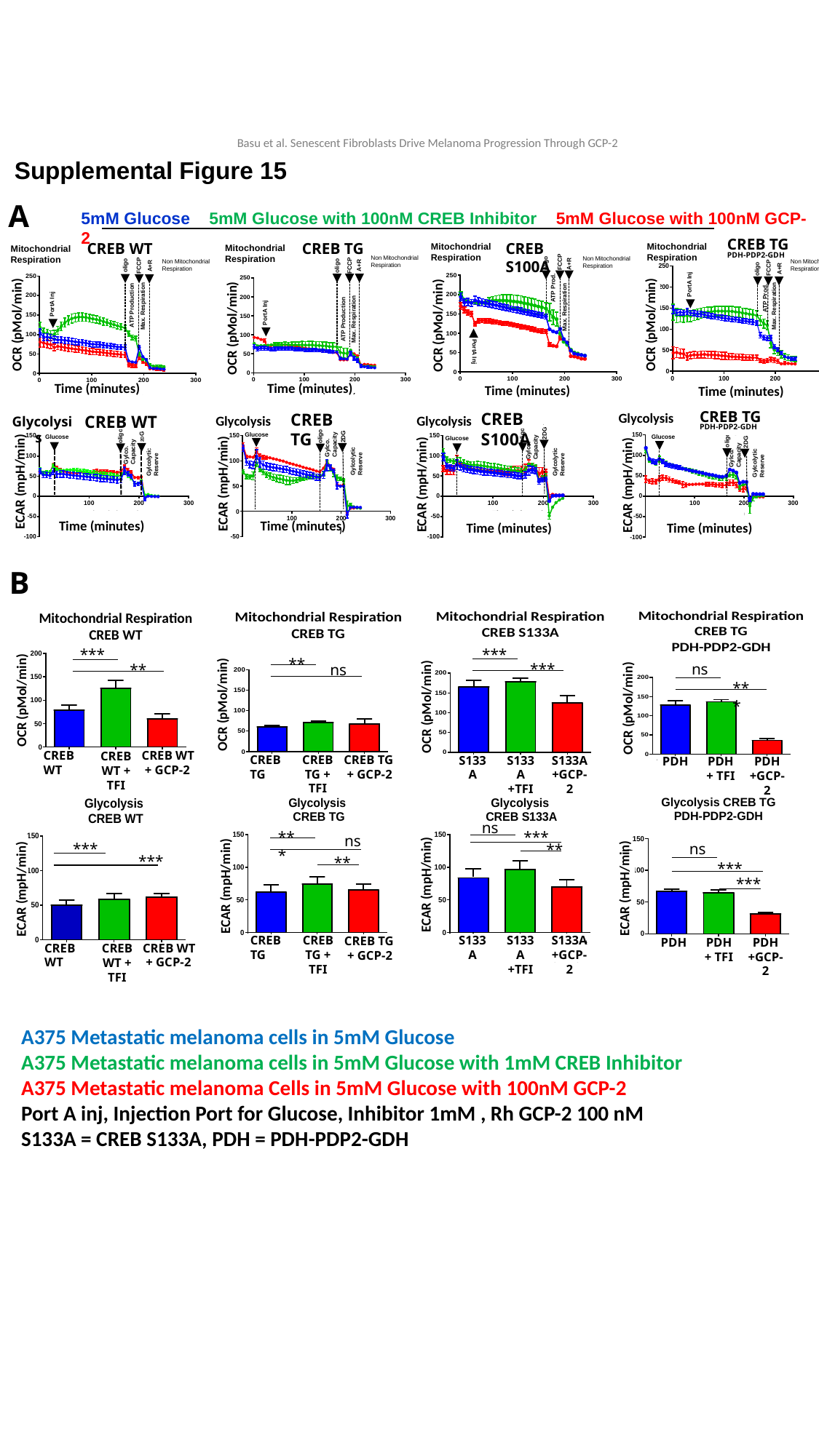

Basu et al. Senescent Fibroblasts Drive Melanoma Progression Through GCP-2
Supplemental Figure 15
A
5mM Glucose 5mM Glucose with 100nM CREB Inhibitor 5mM Glucose with 100nM GCP-2
CREB WT
Mitochondrial
Respiration
FCCP
oligo
A+R
ATP Production
Max. Respiration
PortA Inj
OCR (pMol/min)
Time (minutes)
CREB TG
Mitochondrial
Respiration
Mitochondrial
Respiration
Mitochondrial
Respiration
CREB S100A
Non Mitochondrial
Respiration
oligo
A+R
Max. Respiration
OCR (pMol/min)
Time (minutes)
FCCP
ATP Prod.
PortA Inj
Non Mitochondrial
Respiration
FCCP
A+R
oligo
Max. Respiration
ATP Production
PortA Inj
Time (minutes)
Non Mitochondrial
Respiration
oligo
A+R
Max. Respiration
CREB TG
PDH-PDP2-GDH
Non Mitochondrial
Respiration
FCCP
PortA Inj
ATP Prod.
OCR (pMol/min)
OCR (pMol/min)
Time (minutes)
Glycolysis
CREB WT
Glycolysis
oligo
2DG
Gylco.
Capacity
Glucose
Gylcolytic
Reserve
ECAR (mpH/min)
Time (minutes)
Glycolysis
Glycolysis
CREB S100A
2DG
oligo
Gylco.
Capacity
Glucose
Gylcolytic
Reserve
ECAR (mpH/min)
Time (minutes)
CREB TG
oligo
2DG
Gylco.
Capacity
Gylcolytic
Reserve
oligo
2DG
Gylco.
Capacity
Glucose
Gylcolytic
Reserve
CREB TG
PDH-PDP2-GDH
Glucose
ECAR (mpH/min)
ECAR (mpH/min)
Time (minutes)
Time (minutes)
B
***
***
***
**
**
ns
ns
***
OCR (pMol/min)
OCR (pMol/min)
OCR (pMol/min)
OCR (pMol/min)
CREB WT
CREB WT
 + GCP-2
CREB WT + TFI
CREB TG
CREB TG + TFI
CREB TG
 + GCP-2
S133A
S133A
+TFI
S133A
+GCP-2
PDH
+GCP-2
PDH
PDH
+ TFI
ns
***
***
ECAR (mpH/min)
PDH
+GCP-2
PDH
PDH
+ TFI
ns
***
**
***
ns
**
***
***
ECAR (mpH/min)
ECAR (mpH/min)
ECAR (mpH/min)
S133A
S133A
+TFI
S133A
+GCP-2
CREB TG
CREB TG + TFI
CREB TG
 + GCP-2
CREB WT
CREB WT
 + GCP-2
CREB WT + TFI
A375 Metastatic melanoma cells in 5mM Glucose
A375 Metastatic melanoma cells in 5mM Glucose with 1mM CREB Inhibitor
A375 Metastatic melanoma Cells in 5mM Glucose with 100nM GCP-2
Port A inj, Injection Port for Glucose, Inhibitor 1mM , Rh GCP-2 100 nM
S133A = CREB S133A, PDH = PDH-PDP2-GDH

## Slide 28
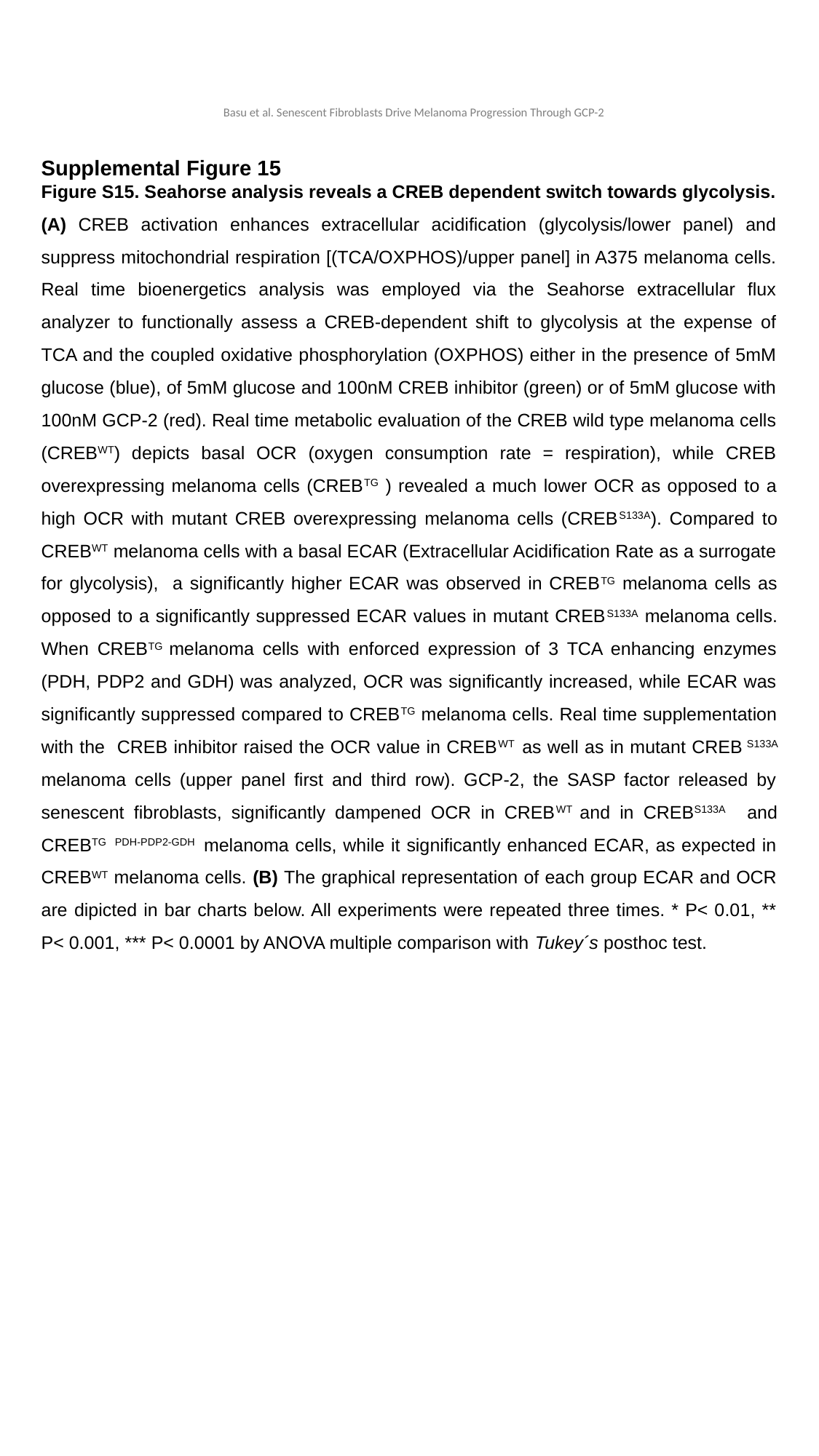

Basu et al. Senescent Fibroblasts Drive Melanoma Progression Through GCP-2
Supplemental Figure 15
Figure S15. Seahorse analysis reveals a CREB dependent switch towards glycolysis.
(A) CREB activation enhances extracellular acidification (glycolysis/lower panel) and suppress mitochondrial respiration [(TCA/OXPHOS)/upper panel] in A375 melanoma cells. Real time bioenergetics analysis was employed via the Seahorse extracellular flux analyzer to functionally assess a CREB-dependent shift to glycolysis at the expense of TCA and the coupled oxidative phosphorylation (OXPHOS) either in the presence of 5mM glucose (blue), of 5mM glucose and 100nM CREB inhibitor (green) or of 5mM glucose with 100nM GCP-2 (red). Real time metabolic evaluation of the CREB wild type melanoma cells (CREBWT) depicts basal OCR (oxygen consumption rate = respiration), while CREB overexpressing melanoma cells (CREBTG ) revealed a much lower OCR as opposed to a high OCR with mutant CREB overexpressing melanoma cells (CREBS133A). Compared to CREBWT melanoma cells with a basal ECAR (Extracellular Acidification Rate as a surrogate for glycolysis), a significantly higher ECAR was observed in CREBTG melanoma cells as opposed to a significantly suppressed ECAR values in mutant CREBS133A melanoma cells. When CREBTG melanoma cells with enforced expression of 3 TCA enhancing enzymes (PDH, PDP2 and GDH) was analyzed, OCR was significantly increased, while ECAR was significantly suppressed compared to CREBTG melanoma cells. Real time supplementation with the CREB inhibitor raised the OCR value in CREBWT as well as in mutant CREB S133A melanoma cells (upper panel first and third row). GCP-2, the SASP factor released by senescent fibroblasts, significantly dampened OCR in CREBWT and in CREBS133A and CREBTG PDH-PDP2-GDH melanoma cells, while it significantly enhanced ECAR, as expected in CREBWT melanoma cells. (B) The graphical representation of each group ECAR and OCR are dipicted in bar charts below. All experiments were repeated three times. * P< 0.01, ** P< 0.001, *** P< 0.0001 by ANOVA multiple comparison with Tukey´s posthoc test.
